# Kenyan sign language word-based pose dataset

**DOI:** 10.1016/j.dib.2025.111502

**Published:** 2025-03-21

**Authors:** Ezekiel Maina, Lilian Wanzare, James Obuhuma, Mildred Ayere, Maurine Kang'ahi, Joel Okutoyi

**Affiliations:** Maseno University, P.O Box Private Bag Maseno, Kenya

**Keywords:** Kenyan sign language, Pose estimation, Stickman representation, Low resource languages

## Abstract

In an era where technology fosters inclusion, sign language remains underrepresented in linguistic datasets, especially for low-resource languages such as Kenyan Sign Language (KSL). This paper presents a novel dataset created using MediaPipe's pose estimation technology, designed to address the scarcity of resources for KSL. The dataset includes 20,000 video recordings of KSL gestures, converted into anonymized stickman representations alongside detailed 3D pose coordinates stored in .npy files. The data collection process focused on preserving participant privacy while ensuring the integrity of gesture data. By utilizing pose estimation, the dataset captures manual and non-manual features of KSL while maintaining the anonymity of signers. Stickman representations abstract human features, mitigating ethical concerns associated with traditional video datasets and aligning with privacy-preserving practices. The dataset spans a diverse range of themes relevant to KSL, including daily interactions, cultural expressions, and educational contexts, providing comprehensive coverage of the KSL lexicon. This dataset is designed for reuse across multiple domains. Researchers can leverage it to train machine learning models for sign language recognition, while educators can utilize it to develop interactive language learning tools. Additionally, it supports the development of virtual sign language interpreters and 3D avatars for accessibility applications. By enabling seamless integration into machine learning frameworks, the dataset facilitates advancements in KSL-related technologies and contributes to bridging communication gaps within the Deaf community.

Specifications Table

**Table:** 

Subject	Computer Vision and Pattern Recognition
Specific subject area	Kenyan Sign Language (KSL) dataset for pose estimation and gesture recognition
Type of data	Figures: Stickman videos representing KSL gestures. Analyzed: 3D pose coordinates stored as .npy files.
Data collection	Videos of KSL gestures were recorded at schools for the Deaf. MediaPipe Holistic was used for pose estimation, and ELAN tool was utilized for segmentation. Stickman animations and pose data were generated from the recordings.
Data source location	Data collected from various Kenyan schools for the Deaf, including Maseno School for the Deaf Primary, ACK Ematundu Boys Secondary and Vocational School for the Deaf, Sikri Technical and Vocational Training Institute for Blind and Deaf, St Angela Girls Mumias Secondary and Vocational School for the Deaf, Fr Ouderaa Secondary School for the Deaf Nyangoma and Ebukuya Primary School for The Deaf Primary.
Data accessibility	Repository Name: Zenodo DOI or Identifier: 10.5281/zenodo.14974973 Direct URL: https://doi.org/10.5281/zenodo.14974973
Related research article	None

## 1 Value of the Data

• Contributes to Underrepresented Sign Language Research: This dataset fills a significant gap in resources for African sign languages, particularly Kenyan Sign Language (KSL). It provides valuable data for training machine learning models tailored to low-resource languages, fostering inclusivity in linguistic and technological research.
• Supports Diverse Applications in Accessibility and Education: The dataset enables the development of applications such as real-time sign language recognition, virtual interpreters, and gesture-based interactive systems for education. These tools can significantly enhance accessibility for Deaf communities and promote inclusive education.
• Ensures Privacy-Preserving Data Collection: By using MediaPipe for pose estimation, the dataset anonymizes participants through stickman animations and 3D pose coordinates, addressing privacy concerns. This approach safeguards the identities of contributors while retaining essential gesture information for research and application.
• Promotes Scalability and Easy Integration: The dataset includes 20,000 videos with corresponding pose data stored in NumPy (.npy) files. This structure allows for seamless integration into machine learning pipelines, facilitating further research in gesture recognition, language learning, and AI-driven accessibility tools.
• Encourages Multimodal Research: The combination of manual (hand gestures) and non-manual (facial expressions, body postures) features captured in the dataset opens doors for advanced multimodal analysis. It supports research in linguistics, machine translation, and synthetic sign language generation.
• Enables Ethical AI Development: The dataset serves as a benchmark for responsible AI in sign language technologies. It demonstrates the importance of balancing data utility with ethical considerations, such as informed consent, anonymity, and inclusivity, in developing tools for underrepresented communities.

## 2 Background

The deaf are a language minority since their mode of communication is used by a very small population. There are 72 million deaf people around the world and it's estimated that more than 80 percent of them live in developing countries [1]. According to [2], Kenya is estimated to have a population of 50 million people with approximately 2.75 million who are deaf. Sign language is an essential medium of communication within the Deaf community. Despite the global recognition of sign languages, underrepresented languages such as Kenyan Sign Language (KSL) face a significant lack of resources, hindering advancements in sign language recognition, educational tools, and accessibility technologies [3]. Kenya's Deaf population benefits from a high degree of standardization in KSL, which is regulated by the Kenya Institute of Curriculum Development (KICD). This ensures consistency in learning and communication, fostering inclusive education and effective interaction across the community. While minor regional variations exist, the core structure of the language remains uniform across institutions.

### 2.1 Manual and non-manual features in sign language

Sign language recognition (SLR) requires the integration of manual and non-manual features to accurately capture the richness of sign language and to convey meaning, emotions, and cultural nuances. Manual features include the core elements of signs such as handshapes, movements, orientations, and locations. These have been widely documented in resources such as the ASL-LEX database [4]. Non-manual features- facial expressions, head tilts and body posture, provide grammatical detail and contextual depth [5]. Together, these elements form a rich and complex linguistic structure. For instance, raised eyebrows in ASL can signal a question, while head tilts in DGS emphasize specific meanings [3,6]. Despite their importance, non-manual features remain underrepresented in most SLR datasets due to the difficulty of consistently capturing these signals [7].

### 2.2 Challenges of dataset availability and privacy

The development of sign language recognition technologies relies heavily on curated datasets. However, existing resources predominantly focus on languages such as American Sign Language (ASL) and German Sign Language (DGS), leaving low-resource languages such as KSL vastly underrepresented [8]. Prominent datasets such as the RWTH-PHOENIX-Weather Corpus [9] and ASL Lexical Dataset [10] have facilitated breakthroughs in gesture recognition and translation for DGS and ASL respectively. Yet, the absence of comprehensive KSL datasets limits the development of inclusive technologies tailored to the Kenyan Deaf community.

Addressing privacy concerns in sign language datasets is crucial, as individuals in smaller communities, such as the Deaf community, may be easily identifiable through video recordings, raising ethical issues around consent and data usage [11]. Privacy-enhancing techniques, such as face or body obfuscation, help balance privacy with data utility [11,12]. A more effective approach is pose estimation, which anonymizes data by focusing on skeletal representations rather than full video footage. Tools such as MediaPipe extract body keypoints while preserving gesture integrity, making them a promising solution [13]. Studies have already leveraged pose data for sign language applications, such as sign language production [14] and gesture-to-text translation [15].

### 2.3 Role of pose estimation in privacy-conscious datasets

Pose estimation technologies, such as MediaPipe and OpenPose, provide an effective method for capturing both manual and non-manual features of sign language while safeguarding signer privacy. These tools generate skeletal representations that abstract away personal details, enabling the creation of datasets such as POSE-ASL, which successfully employs pose data for ASL recognition [16]. By leveraging similar methods, researchers can develop scalable, privacy-conscious datasets that address the underrepresentation of sign languages such as KSL.

Advances in pose estimation technologies enable an improved way of simultaneously capturing manual and non-manual markers, offering a privacy-conscious and efficient way to enhance the comprehensiveness of SLR datasets [17].

### 2.4 Advancing KSL research with a novel dataset

To bridge the gap in KSL research, this work introduces a novel dataset created using MediaPipe's pose estimation capabilities. The dataset consists of anonymized stickman videos representing word-based KSL gestures, along with pose coordinates stored in .npy files. This structure ensures the privacy of signers while providing a resource compatible with machine learning frameworks. By addressing data scarcity and privacy concerns, this dataset fosters advancements in KSL recognition, education, and accessibility, contributing to the inclusion of sign languages in the digital era.

## 3 Dataset Description

The dataset contains a comprehensive collection of KSL gesture videos, which are designed for machine learning applications in sign language recognition or other sign language tasks. Below is an overview of the dataset's key components:

### 3.1 Number of word based videos

The dataset comprises around 30000 videos, each named after a word and capturing distinct KSL gestures, with each clip representing a KSL word or phrase. The word videos are derived from original videos of signed sentences which are segmented with the start and end of each signed word in the sentence and meticulously labeling the segments with the word that is being signed [18]. The vocabulary spans various themes, including daily interactions, educational topics, and broader KSL lexicon. A document detailing the word distribution of the videos is in the dataset repository.

### 3.2 Frame rate and resolution of the stickman videos

The stickman animations generated from pose estimation as featured in Fig. 8 have the same frame rate as the original videos, with the frame rate typically being 25-30 frames per second (fps). The resolution of the stickman videos matches that of the original videos, which were recorded at standard video resolutions such as 1920×1080 pixels. This ensures that the gesture movements are captured in sufficient detail for further processing and analysis.

### 3.3 Format of the pose coordinates

**Fig. 1:**
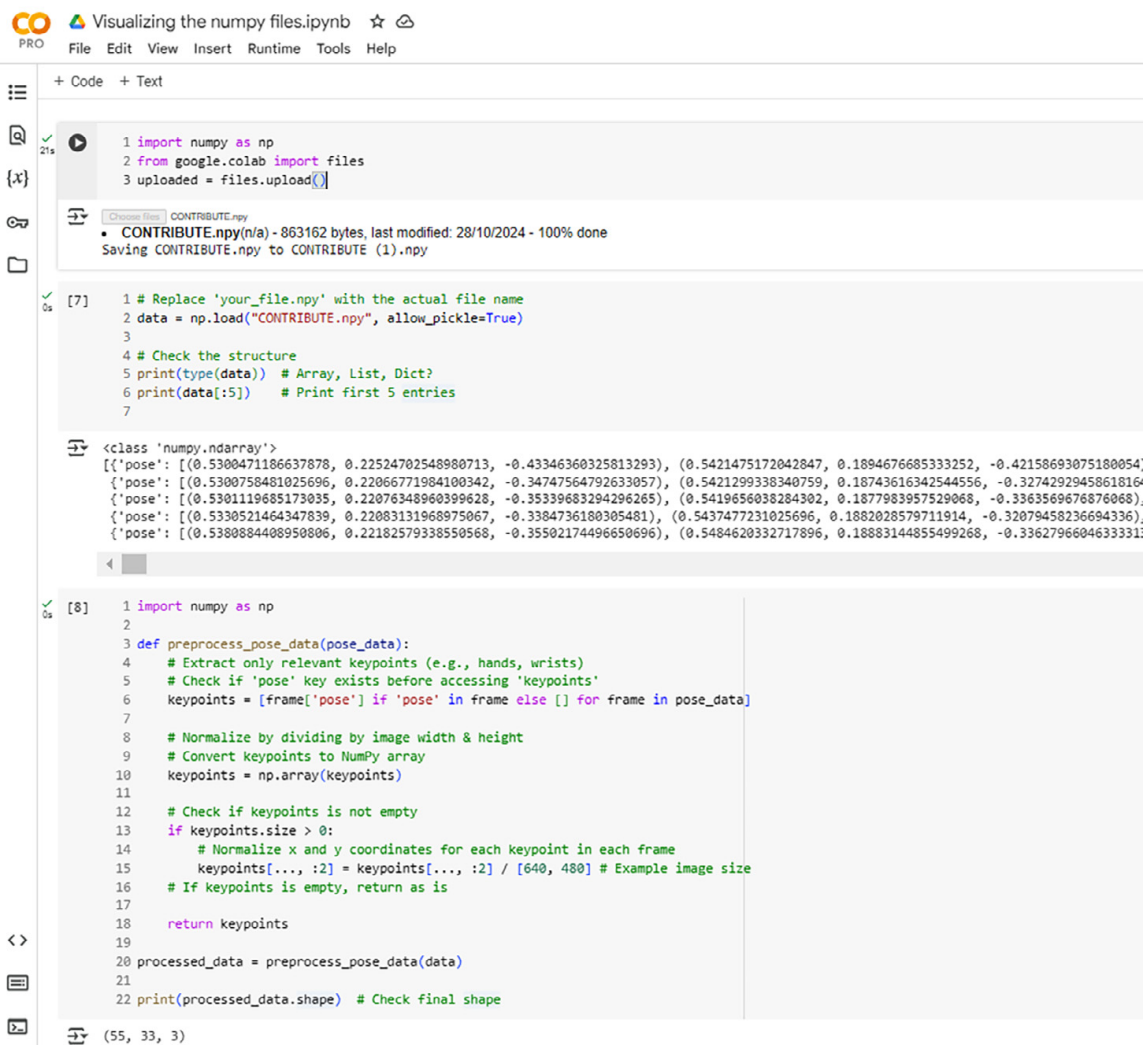
Reading NPY File with landmarks using python.

Each video has an associated NumPy (.npy) file, where the pose coordinates for each frame are stored. The pose coordinates represent 33 keypoints per frame, capturing the critical body landmarks for sign language gestures. Each keypoint includes x, y, and z coordinates, representing the 3D spatial location of the keypoint relative to the video frame. This data structure allows researchers to analyze the movement and positioning of body parts such as the shoulders, elbows, wrists, and hands in a sign language gesture. Fig. 1 provides a sample view of how the .npy file is organized and described for sign language applications. The overall structure of the NumPy files consists of frames representing the number of time steps in the video, keypoints corresponding to the detected body landmarks, and coordinates containing the x, y, and z values for each keypoint.

The dataset is designed for scalability, with plans to expand its size by incorporating additional KSL gesture videos over time, thereby enriching the diversity and comprehensiveness of the collection.

## 4 Experimental Design, Materials and Methods

The Kenyan Sign Language Word-Based Pose Dataset was developed through a systematic and privacy-conscious approach, ensuring accurate data collection and processing while addressing the ethical requirements of working with underrepresented communities. The process involved recording, segmentation, and pose estimation to create an anonymized dataset suitable for gesture recognition and linguistic research.

### 4.1 Data collection

The collection of the KSL video dataset was carried out through a systematic process designed to capture the richness and diversity of KSL across various subject domains in the Kenyan curriculum. Researchers visited multiple schools for the deaf, ensuring the inclusion of a wide range of signs across different class levels and guaranteeing representation of gender variability among the signers.

**Fig. 2:**
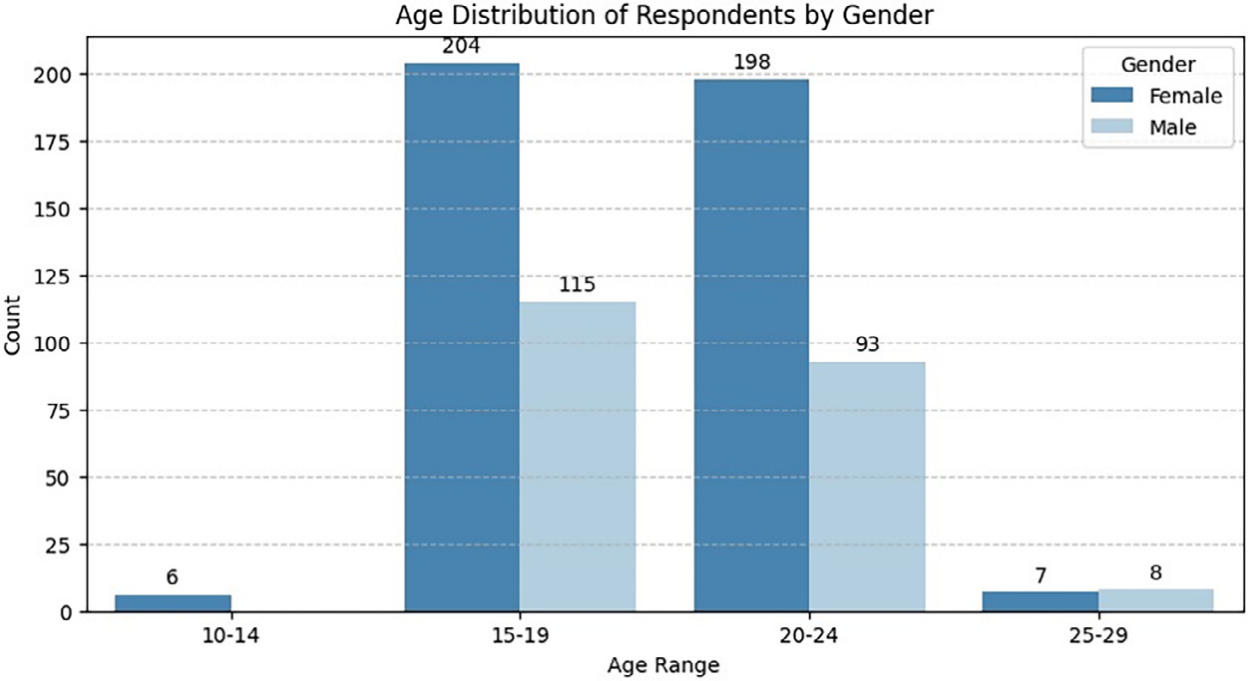
Age distribution of respondents by gender.

Fig. 2 shows the age distribution of respondents across four age groups: 10-14, 15-19, 20-24, and 25-29. The 15-19 age group has the highest count, slightly above 300, followed closely by the 20-24 group with just under 300 respondents. The 10-14 and 25-29 groups have significantly lower numbers, with very few respondents.

This indicates that most participants are teenagers and young adults, with minimal representation from younger and older groups.

**Fig. 3:**
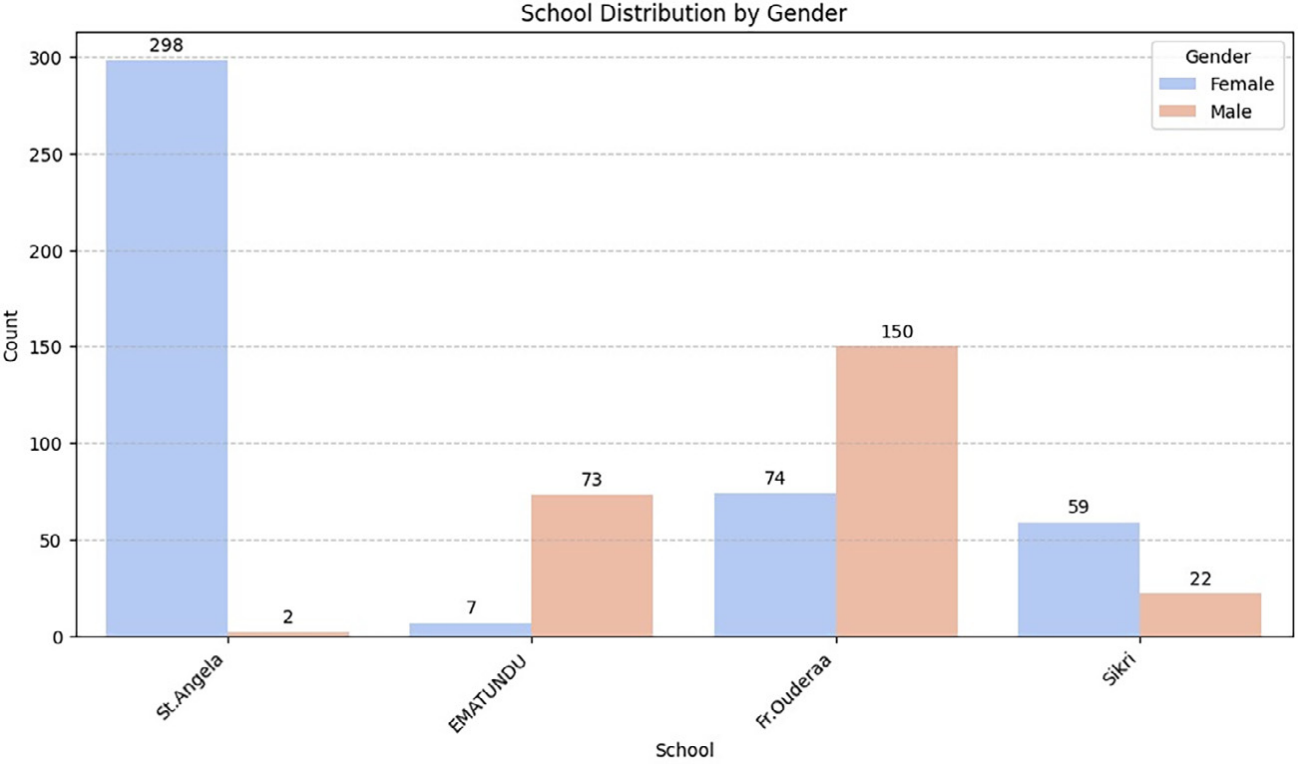
School distribution by gender.

Fig. 3 illustrates the gender distribution of students across various schools. St. Angela is an all-girls school, the males represented are staff. Similarly, EMATUNDU is an all-boys school and the number of females shown belong to the staff. Fr. Ouderaa has a higher number of male students compared to females, whereas Sikri has a slightly larger female population.

**Fig. 4:**
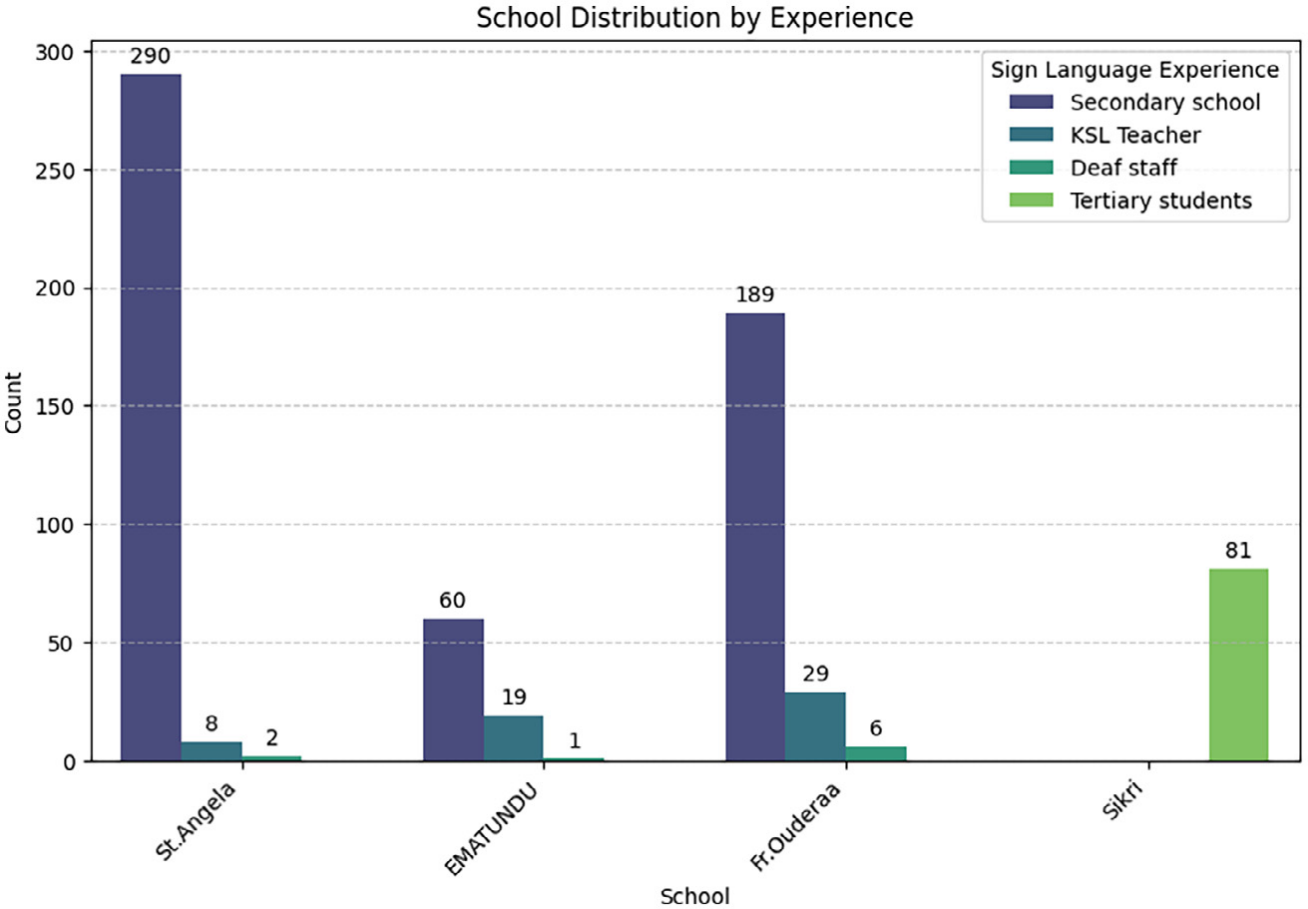
School distribution by experience.

Fig. 4 illustrates the distribution of sign language experience, St. Angela and Fr. Ouderaa have the highest number of students in the ``Secondary school'' category, while Ematundu has a mix of secondary students and KSL teachers. Sikri stands out with the highest number of ``KSL Signers''. ``Deaf staff'' are the least represented across all schools.

Generally, the 685 participants, were all students and staff who were willing to participate and had a good command of KSL. All those who were interested were allowed to participate as most schools have less than 300 students, then the students come from different parts of the country especially the boarding school students.

During this phase, videos were recorded using a front-facing camera to document various KSL signs. To ensure a comprehensive representation of sign variability, at least three signers were recorded for each sentence.

This approach allowed for a better understanding of how different individuals express the same signs, reflecting the diversity inherent in KSL.

Following the video recording phase, a meticulous cleaning and preprocessing stage was implemented. All recorded videos were reviewed and split using the Shortcut video editor to ensure that each video represented a single word or sentence. This was crucial for maintaining the clarity and usability of the dataset.

In the next preprocessing step, the ELAN tool was employed to segment the videos. Researchers marked the start and end of each sign, ensuring accurate representation of the signing actions. Additionally, linguistic tiers were defined within the ELAN tool, establishing three specific tiers: English Sentence, Gloss and Finger Spelling. The inclusion of a finger-spelling tier was essential to distinguish sections that involved fingerspelling from other signs, enhancing the dataset's linguistic richness.

By combining these structured data collection and preprocessing methods, the project aimed to create a robust dataset that accurately captures the nuances of KSL, ultimately contributing to research and educational resources in the field of sign language studies [19].

### 4.2 Video segmentation

The segmentation process for videos based on ELAN (.eaf) file annotations begins by running a dedicated segmentation script. This script first loads each .eaf file from a specified directory and identifies the corresponding video file in .mp4 format. Once both the .eaf and video files are located, the script extracts tier information from the .eaf file, focusing particularly on tiers such as ``English,'' ``Gloss,'' and ``Fingerspelling.'' The English Tier contains the spoken or written translation, often serving as a textual representation in English. The Gloss Tier represents a simplified, word-for-word transcription of the signs using written labels, typically in capital letters (e.g., EAT, HOUSE), rather than a full grammatical translation, making it useful for linguistic analysis. The Fingerspelling Tier captures instances where individual letters are spelled out using the fingerspelling system of KSL, which is crucial for recognizing proper nouns, technical terms, and loanwords.

**Fig. 5:**
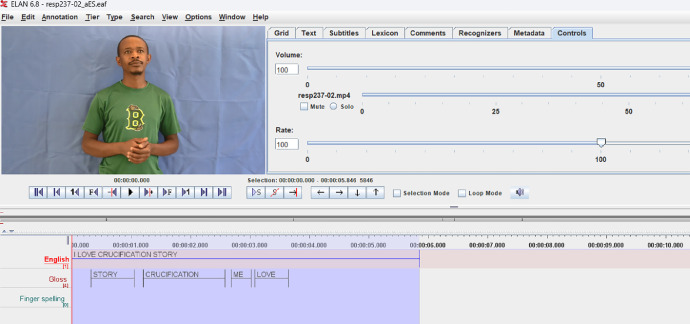
Video segmentation using ELAN tool.

Within each of these tiers, individual annotations are retrieved, including essential details such as the start and end times and any associated text annotations. An instance of the segmentation is shown in Fig. 5, with the signer's consent obtained.

For each annotation within the ``Gloss'' and ``Fingerspelling'' tiers, the script calculates the start and end times in seconds, converting from the default milliseconds. Using these times, it then extracts each annotated segment as a separate video clip, naming each output clip according to the gloss or fingerspelled word associated with the annotation. Each clip is saved in an output directory, making it easy to access the segmented files.

This process is repeated for each .eaf file in the directory, allowing the script to handle multiple files in succession.

Once all files have been processed, the segmentation is complete, resulting in a set of video clips that align with the annotations in the ELAN files. This structured approach enables efficient segmentation of videos based on annotated gestures or words, facilitating further analysis or educational use.

**Fig. 6:**
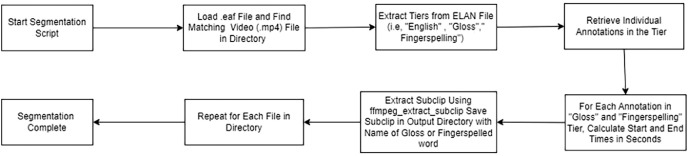
Segmentation process based on the tiers.

This segmentation can be summarised as in Fig. 6.

### 4.3 Pose estimation

Pose estimation was achieved using MediaPipe Holistic, a powerful tool capable of tracking multiple body landmarks, including the pose, hands, and face. The use of pose estimation tools such as MediaPipe ensures that these subtle, yet crucial, elements are accurately represented without compromising privacy. By converting video data into anonymized 3D stickman representations, the dataset allows for detailed gesture analysis while protecting the identity of participants.

To analyze body landmarks, MediaPipe requires the video to be broken down into individual frames. OpenCV facilitates this by extracting frames one at a time, allowing MediaPipe to process each frame independently. Additionally, OpenCV offers pre-processing capabilities, such as resizing, color adjustments, and noise reduction, which enhance video quality and improve the accuracy and reliability of MediaPipe's key point extraction.

An illustration of how the pose estimation was performed and a breakdown of the process is shown in Fig. 9 and described below:

### 4.4 Video processing

The video files were opened with OpenCV's cv2.VideoCapture(), enabling frame extraction. The video properties such as frames per second (FPS), frame dimensions, and the total number of frames are extracted. Each video frame was processed sequentially in a loop. Since OpenCV captures frames in **BGR (Blue, Green, Red)** format by default, the frames were then converted to **RGB (Red, Green, Blue)** format using OpenCV's *cv2.cvtColor()* function. This conversion is necessary because MediaPipe models are designed to work with RGB images. Converting the frames from **BGR to RGB** before passing it into MediaPipe ensures that the model receives the image data in the correct format, leading to accurate results as color channels won't be considered. This is why the conversion step is **necessary**. The output here is video frames in RGB Format.

### 4.5 Landmark extraction

**Fig. 7:**
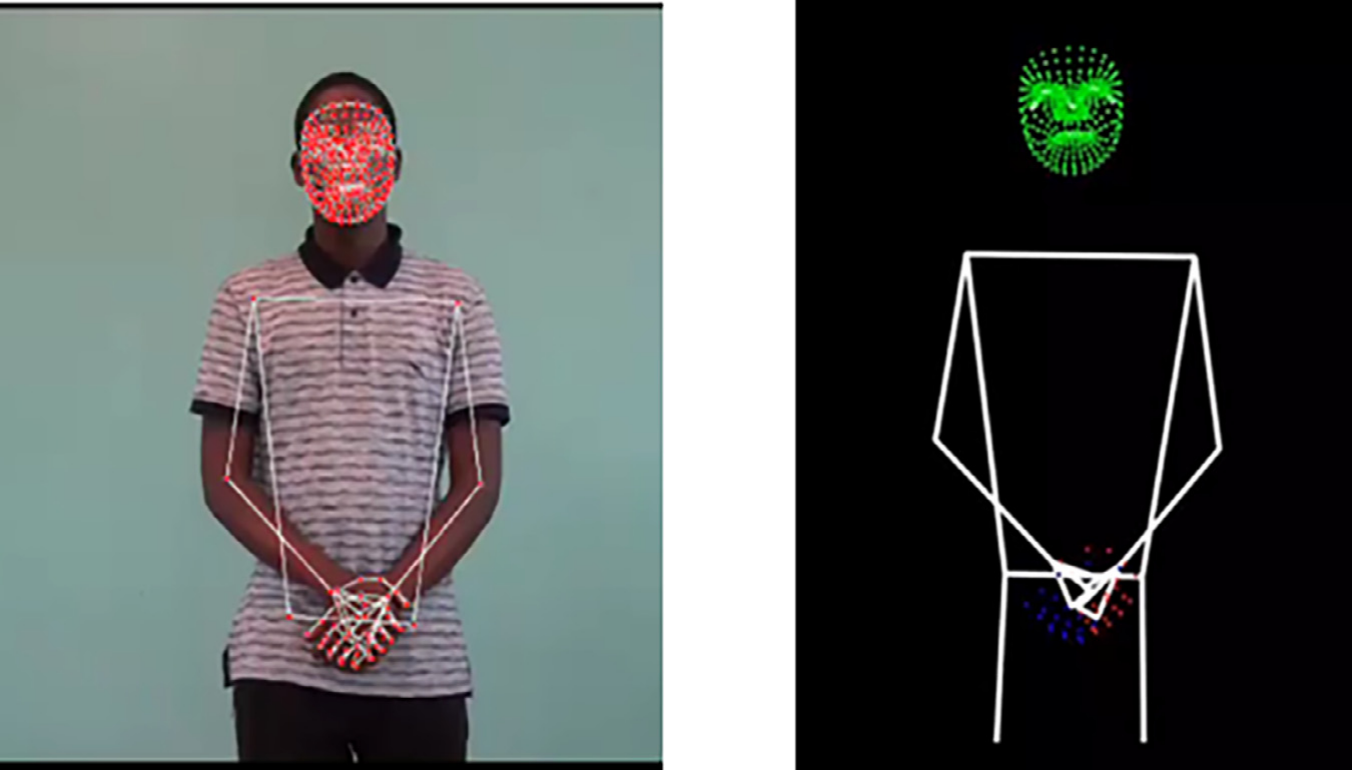
Pose estimation.

The landmark extraction process is conducted using the Mediapipe library, specifically the Holistic model, which detects a variety of human body landmarks, including those for the face, hands, and body pose as shown in Fig. 7. This process uses the video processing output as input. The holistic model is initialized with a minimum detection confidence of 0.5 and a minimum tracking confidence of 0.5, which controls how well the model detects and tracks the landmarks in each frame.

For each frame in the video, the holistic model processes the frame to detect landmarks using the holistic.process() method, which includes:

i. **Pose landmarks:** For pose landmarks, MediaPipe tracks 33 keypoints across the full body, including the arms, shoulders, wrists, fingers, torso, and legs. In this study, our focus is on the upper body, particularly keypoints such as the shoulders, elbows, and wrists, which are crucial for capturing gestures used in sign language as featured in Fig. 5, Fig. 7. Excluding the lower body leaves us with 25 relevant keypoints concentrated on the upper body.
  Although our analysis prioritized these 25 upper-body points, the MediaPipe library was not limited to any landmarks. This comprehensive landmark extraction offers a significant advantage: it provides the flexibility to expand the analysis to include additional body movements if needed in the future. Such adaptability could be particularly valuable for applications beyond gesture recognition, such as full-body motion capture or postural analysis, without requiring further configuration or reprocessing.
ii. **Hand Landmarks**: For hand landmarks, MediaPipe detected 21 points per hand, allowing us to capture detailed hand gestures, a critical component of sign language.
iii. **Face Landmarks**: MediaPipe's 468 facial landmarks captured key features such as the eyes, eyebrows, nose, and mouth, which were essential for interpreting non-manual signals in sign language.

### 4.6 Stickman generation and landmark storage

**Fig. 8:**
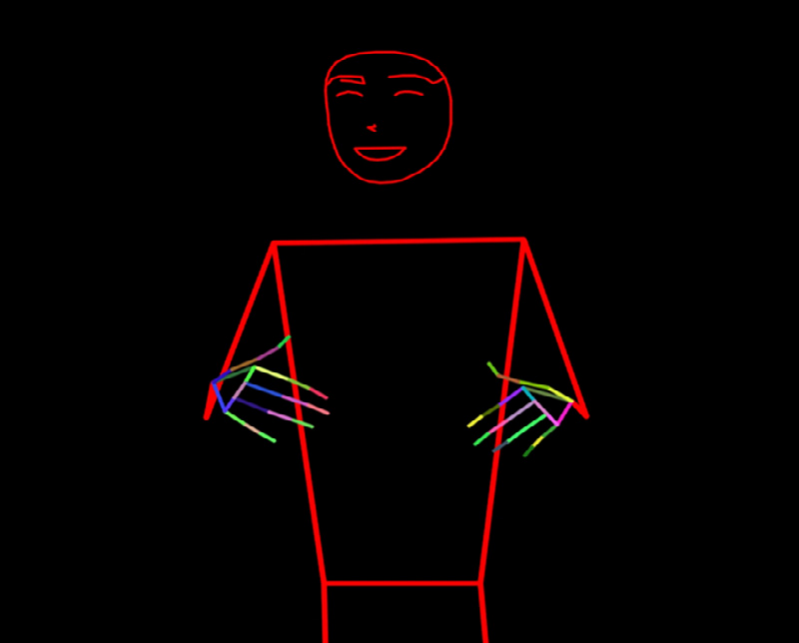
Stickman generation.

Once the pose landmarks were extracted as featured in Fig. 8, we:

**Fig. 9:**
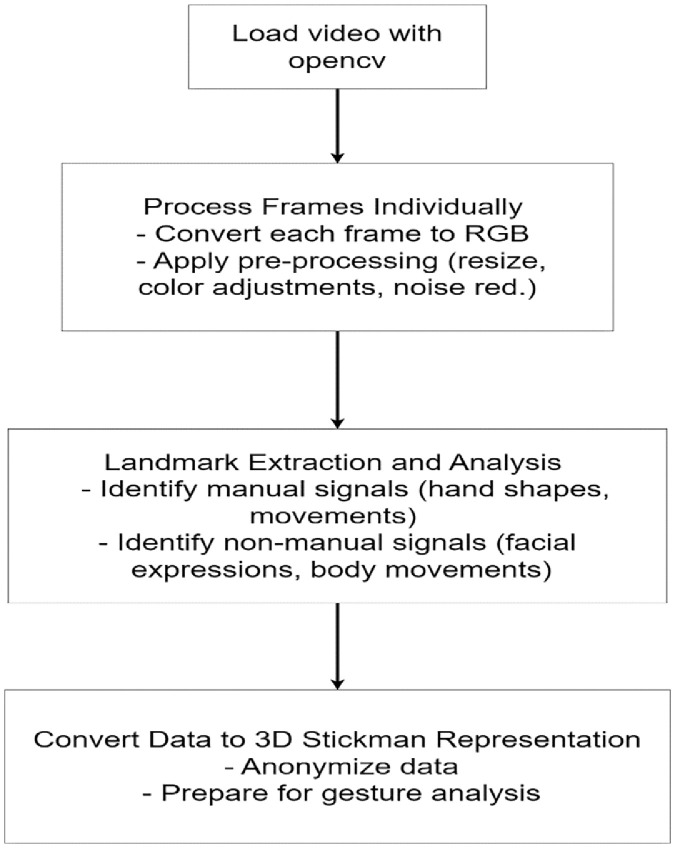
Pose estimation and stickman generation process.

### 4.7 Created stickman animations from the landmarks

The stickman figures represent the human skeleton by connecting the keypoints identified by MediaPipe. For each frame, lines were drawn between relevant pose keypoints (such as shoulders to elbows and elbows to wrists) to visualize the movement of the body during the signing.

To ensure that facial details were captured in the stickman motion, the following connections were considered:

**POSE_CONNECTIONS_UP_TO_ELBOW, FACE_CONNECTIONS, MOUTH_CONNECTIONS, EYE_CONNECTIONS, EYEBROW_CONNECTIONS** and **NOSE_CONNECTIONS**. These connections help capture the essential aspects of facial expressions, which are integral to conveying meaning in KSL. The connections were defined as follows:

**POSE_CONNECTIONS_UP_TO_ELBOW** = [ (11, 12), (11, 23), (12, 24), (23, 24), (23, 25), (24, 26), (25, 27), (26, 28), (27, 29), (28, 30), (29, 31), (30, 32), (11, 13), (12, 14)]

**FACE_CONNECTIONS** = [(10, 338), (338, 297), (297, 332), (332, 284), (284, 251), (251, 389), (389, 356), (356, 454), (454, 323), (323, 361), (361, 288), (288, 397), (397, 365), (365, 379), (379, 378), (378, 400), (400, 377), (377, 152), (152, 148), (148, 176), (176, 149), (149, 150), (150, 136), (136, 172), (172, 58), (58, 132), (132, 93), (93, 234), (234, 127), (127, 162), (162, 21), (21, 54), (54, 103), (103, 67), (67, 109), (109, 10)]

**MOUTH_CONNECTIONS** = [(61, 146), (146, 91), (91, 181), (181, 84), (84, 17), (17, 314), (314, 405), (405, 321), (321, 375), (375, 291), (291, 61)]

**EYE_CONNECTIONS** = [(33, 246), (246, 161), (161, 160), (160, 159), (159, 158), (158, 157), (157, 173), (173, 133), (263, 466), (466, 388), (388, 387), (387, 386), (386, 385), (385, 384), (384, 398), (398, 362)]

**EYEBROW_CONNECTIONS** = [(70, 63), (63, 105), (105, 66), (66, 107), (107, 55), (55, 65), (65, 52), (336, 296), (296, 334), (334, 293), (293, 300), (300, 301), (301, 251)]

**NOSE_CONNECTIONS** = [(94, 2), (2, 97), (97, 164)]

The facial landmarks are connected using predefined sets of face connections, such as FACE_CONNECTIONS, MOUTH_CONNECTIONS, EYE_CONNECTIONS, EYEBROW_CONNECTIONS, and NOSE_CONNECTIONS. These sets define pairs of facial landmark indices, which are then used to draw lines between the detected points, rendering key features of the face on each frame.

For hand landmarks, we used the Mediapipe HAND_CONNECTIONS to connect landmarks of both the left and right hands. These hand landmarks are drawn with specific connection lines, allowing the hands to be visualized with multicolored lines for each finger joint. The hand landmarks are processed and drawn on the frame using mp_drawing.draw_landmarks(), with the drawing style specified (such as color, thickness, and circle radius).

For body pose landmarks, we targeted the pose up to the elbow, using a custom connection set called POSE_CONNECTIONS_UP_TO_ELBOW, which includes connections such as the hips, knees, and feet, as well as the upper body from shoulder to elbow. These connections are drawn by iterating over pairs of landmarks defined in the POSE_CONNECTIONS_UP_TO_ELBOW list, with each frame showing the body structure through connected lines. The pose landmarks are processed and then drawn on the frame, focusing primarily on the body's upper structure.

The stickman representations of the signer are drawn on a blank canvas, using the above connections, for each video frame, simplifying the human figure into a stick figure as illustrated in Fig. 8. These frames are then compiled into a new output video file in MP4 format, providing a visual depiction of the landmarks in a stick figure format for every frame in an abstracted, privacy-respecting form.

These stickman animations are highly useful for gesture recognition and other machine learning applications, while ensuring the anonymity of the participants.

### 4.8 Stored the landmarks

The landmarks for all detected features (face, hands, and body pose) are stored in a dictionary for each frame, and these landmarks are collected into a list (all_landmarks). This list is then saved as a .npy file, allowing the landmarks to be saved for later use. If no landmarks are detected in a particular frame, a message is printed indicating the absence of landmarks.

The npy files and the stickman video properties are discussed in the dataset description section.

The data [20] refers to NumPy and MP4 files containing pose estimation results, which are processed and uploaded. Fig. 1 illustrates an instance of accessing a NumPy file in Python within a Google Colab environment. The processed data's shape is then printed to verify the transformation, ensuring it is ready for further applications such as sign language recognition. The notebook is shared in the dataset space [20].

## Limitations

The Kenyan Sign Language Word-Based Pose Dataset has several limitations related to data collection and curation. It focuses mainly on the signing space, capturing upper-body movements. However, the facial landmarks may lack the precision required to accurately represent subtle non-manual cues. The dataset includes around 30,000 gestures but does not cover the full KSL lexicon, leaving out specialized terms and regional variations. Data collection occurred in controlled environments, potentially limiting natural variability seen in everyday communication. The reliance on MediaPipe for pose estimation introduces minor inaccuracies, especially for rapid or complex gestures. These factors highlight areas for improvement in future data collection.

## Ethics Statement

The creation and dissemination of the Kenyan Sign Language Word-Based Pose Dataset adhered to strict ethical guidelines to ensure the protection and privacy of participants. Ethical approval for this research was granted by the Maseno University Scientific and Ethics Review Committee (MUSERC) under protocol number MSU/DRPI/MUSERC/01187/23, ensuring compliance with established standards for research involving human subjects.

Informed consent was obtained from all participants, including adults and students, involved in the data collection process. Participants were fully briefed on the nature of the research, the intended use of their data, and their right to withdraw at any time without repercussions. Given the sensitive nature of working with a vulnerable community, all video recordings were anonymized using pose estimation technologies. This process transformed the visual data into stickman animations and 3D pose coordinates, effectively removing identifiable features and safeguarding the privacy and confidentiality of participants.

The dataset was designed to exclude personal identifiers and was securely stored and shared in strict adherence to privacy and data protection regulations. Efforts were also made to represent the diversity of the Deaf community, including a wide range of signers to capture variations in regional dialects and personal signing styles, thereby minimizing bias. This ethical framework, overseen by MUSERC, ensures that the dataset contributes responsibly to research and development in sign language recognition, advancing the field while upholding the rights and dignity of all participants.

## CRediT authorship contribution statement

**Ezekiel Maina:** Conceptualization, Methodology, Visualization, Software, Investigation, Writing – original draft, Writing – review & editing. **Lilian Wanzare:** Supervision, Writing – review & editing. **James Obuhuma:** Supervision, Writing – review & editing. **Mildred Ayere:** Data curation, Formal analysis. **Maurine Kang'ahi:** Data curation, Visualization. **Joel Okutoyi:** Data curation, Validation.

## Data Availability

ZenodoKSL WORD BASED POSE DATASET (Original data).

## Appendix Supplementary materials

Image, application 1

Image, application 2

Image, application 3
