# Supplementary material for: Kenyan sign language word-based pose dataset
Source: Data Brief. 2025 Mar 21;60:111502. doi: 10.1016/j.dib.2025.111502 (PMC11999445; doi:10.1016/j.dib.2025.111502)
Supplement: Supplementary file 1 [file mmc1.pdf]

MASENO UNIVERSITY SCIENTIFIC AND ETHICS REVIEW COMMITTEE

Tel: +254 057 351 622 Ext: 3050
Fax: +254 057 351 221

Private Bag – 40105, Maseno, Kenya
Email: muerc-secretariate@maseno.ac.ke

REF: MSU/DRPI/MUSERC/01187/23

Date: 10th February, 2023

TO: Dr. Lilian Diana Awuor Wanzare
Department of Computer Science
School of Computing and Informatics
Maseno University
P. O. Box, Private Bag, Maseno, Kenya

Dear Madam,

RE: AI4KSL: Bridging Language Barrier using Artificial Intelligence for Kenyan Sign Language among Deaf Learners

This is to inform you that **Maseno University Scientific and Ethics Review Committee (MUSERC)** has reviewed and approved your above research proposal. Your application approval number is MUSERC/01187/23**.**The approval period is 10th February, 2023 – 9th February, 2024.

This approval is subject to compliance with the following requirements;

i. Only approved documents including (informed consents, study instruments, MTA) will be used.
ii. All changes including (amendments, deviations, and violations) are submitted for review and approval by Maseno University Scientific and Ethics Review Committee (MUSERC).
iii. Death and life threatening problems and serious adverse events or unexpected adverse events whether related or unrelated to the study must be reported to Maseno University Scientific and Ethics Review Committee (MUSERC) within 24 hours of notification.
iv. Any changes, anticipated or otherwise that may increase the risks or affected safety or welfare of study participants and others or affect the integrity of the research must be reported to Maseno University Scientific and Ethics Review Committee (MUSERC) within 24 hours.
v. Clearance for export of biological specimens must be obtained from relevant institutions.
vi. Submission of a request for renewal of approval at least 60 days prior to expiry of the approval period. Attach a comprehensive progress report to support the renewal.
vii. Submission of an executive summary report within 90 days upon completion of the study to Maseno University Scientific and Ethics Review Committee (MUSERC).

Prior to commencing your study, you will be expected to obtain a research license from National Commission for Science, Technology and Innovation (NACOSTI) https://oris.nacosti.go.ke and also obtain other clearances needed.

Yours sincerely

MASENO UNIVERSITY ETHICS REVIEW 10 FEB 2023 COMMITTEE P.O. BOX PRIVATE BAG MASENO

Prof. Philip O. Owuor, PhD, FAAS, FKNAS
Chairman, MUSERC
