# Supplementary material for: Kenyan sign language word-based pose dataset
Source: Data Brief. 2025 Mar 21;60:111502. doi: 10.1016/j.dib.2025.111502 (PMC11999445; doi:10.1016/j.dib.2025.111502)
Supplement: Supplementary file 3 [file mmc3.pdf]

AI4KSL VIDEO PROCESSING PHASE I

Total Video Count: 21482
The tables below show the respective words and their frequency

**DUPLICATE COUNTS-** Duplicate Video Files/Words		
**ROW 1**	**ROW 2**	**ROW 3**
File: .mp4, Count: 76	File: LIE, Count: 8	File: GOVERNMENT, Count: 19
File: ALL, Count: 104	File: LIST, Count: 4	File: IMPORTANT, Count: 31
File: ALLOWANCE, Count: 4	File: LITERATURE, Count: 4	File: INCREASE, Count: 7
File: ALWAYS, Count: 159	File: METHOD, Count: 7	File: MANAGER, Count: 16
File: ASSOCIATION, Count: 7	File: NEXT, Count: 58	File: NINE, Count: 3
File: ATTEND, Count: 11	File: NOT, Count: 11	File: ORGANISATION, Count: 4
File: BANK, Count: 19	File: PROBLEM, Count: 20	File: PAY, Count: 18
File: BEFORE, Count: 66	File: QUESTION, Count: 12	File: RECEIVE, Count: 17
File: BOSS, Count: 23	File: RAIN, Count: 26	File: RIGHT, Count: 8
File: CARD, Count: 4	File: SIGNATURE, Count: 3	File: SALARY, Count: 6
File: CHEF, Count: 14	File: SOLUTION, Count: 6	File: SECTION, Count: 4
File: CHILD, Count: 36	File: SOME, Count: 50	File: STAFF, Count: 3
File: COW, Count: 17	File: SUBJECT, Count: 6	File: STRIKE, Count: 15
File: DEAF, Count: 13	File: THESE, Count: 20	File: UNIVERSITY, Count: 13
File: DIFFERENT, Count: 10	File: TOMORROW, Count: 103	File: WELL, Count: 59
File: DO, Count: 28	File: TOPIC, Count: 9	File: AWAY, Count: 16
File: FINISH, Count: 135	File: WE, Count: 91	File: BACK, Count: 22
File: FORM, Count: 8	File: WRONG, Count: 10	File: BLOCK, Count: 11
File: GIVE, Count: 75	File: AGRICULTURE, Count: 5	File: CLASS, Count: 45
File: GO, Count: 109	File: EVENING, Count: 12	File: CLIENT, Count: 7
File: GROUP, Count: 9	File: FIND, Count: 13	File: DEFEND, Count: 14
File: HOTEL, Count: 7	File: HIGH, Count: 21	File: DESK, Count: 13
File: IDENTIFICATION, Count: 2	File: LANGUAGE, Count: 17	File: ENTRANCE, Count: 11

File: JOB, Count: 10	File: LECTURE, Count: 8	File: GIFT, Count: 19
File: KEEP, Count: 22	File: AREA, Count: 7	File: GUARD, Count: 22
File: LATELY, Count: 4	File: CARE, Count: 20	File: HIS, Count: 91
File: LETTER, Count: 18	File: CLOUDS, Count: 3	File: HOLY, Count: 23
File: MANY, Count: 142	File: COLOUR, Count: 19	File: LAWYER, Count: 12
File: MEETING, Count: 44	File: DESTROY, Count: 9	File: MILITARY, Count: 4
File: MINE, Count: 289	File: DRESS, Count: 17	File: MOVE, Count: 19
File: MONEY, Count: 36	File: DRY, Count: 16	File: OPPONENT, Count: 12
File: MUST, Count: 132	File: EARTHQUAKE, Count: 5	File: PRESIDENT, Count: 29
File: OFFICE, Count: 35	File: ENVIRONMENT, Count: 12	File: PROMISE, Count: 17
File: OFFICIAL, Count: 19	File: GRASS, Count: 7	File: PROTECT, Count: 18
File: OUR, Count: 166	File: LIGHT, Count: 36	File: PULL, Count: 7
File: PAST, Count: 264	File: LIGHTENING, Count: 5	File: RACE, Count: 18
File: PEOPLE, Count: 134	File: MONTH, Count: 42	File: ROAD, Count: 33
File: PROFIT, Count: 8	File: MOUNTAIN, Count: 8	File: ROPE, Count: 5
File: REPORT, Count: 10	File: PROPERTY, Count: 19	File: SOLDIER, Count: 12
File: ROBBER, Count: 12	File: RAINBOW, Count: 4	File: SPIRIT, Count: 11
File: SAME, Count: 94	File: RIVER, Count: 24	File: TEAM, Count: 28
File: SCHOOL, Count: 92	File: SKY, Count: 9	File: TELL, Count: 22
File: TEACHER, Count: 88	File: TODAY, Count: 82	File: THEIR, Count: 25
File: THAT, Count: 296	File: WINTER, Count: 4	File: VOW, Count: 7
File: THIEF, Count: 27	File: YOUR, Count: 172	File: ACCOUNT, Count: 8
File: TIME, Count: 180	File: AGAIN, Count: 11	File: COME, Count: 48
File: WORK, Count: 161	File: AMERICA, Count: 20	File: HOME, Count: 30
File: WORKER, Count: 24	File: CHILDREN, Count: 53	File: LEAD, Count: 7
File: WRITE, Count: 27	File: CLOSE, Count: 23	File: RULE, Count: 6
File: YESTERDAY, Count: 69	File: EXAMS, Count: 3	File: SOCIETY, Count: 8
File: BAG, Count: 19	File: FUTURE, Count: 139	File: VISIT, Count: 26

File: BOOK, Count: 68	File: GET, Count: 32	File: ASSIGNMENT, Count: 9
File: BOY, Count: 52	File: GOOD, Count: 78	File: BASKET, Count: 12
File: CLASSROOM, Count: 8	File: HAVE, Count: 255	File: CAREFUL, Count: 10
File: CONTENT, Count: 4	File: HOSPITAL, Count: 10	File: FIX, Count: 11
File: ENGLISH, Count: 5	File: LUNCH, Count: 8	File: HUNTER, Count: 9
File: GEOGRAPHY, Count: 6	File: MONDAY, Count: 14	File: LEAK, Count: 9
File: GRADUATE, Count: 9	File: MORNING, Count: 28	File: PREPARE, Count: 10
File: GRANDMOTHER, Count: 20	File: NEW, Count: 90	File: ROOF, Count: 13
File: HE, Count: 134	File: NIGHT, Count: 21	File: SHOOT, Count: 18
File: HEADTEACHER, Count: 17	File: NOON, Count: 8	File: TARGET, Count: 17
File: ME, Count: 323	File: PLAY, Count: 31	File: TURN OFF, Count: 2
File: PEN, Count: 23	File: RECENTLY, Count: 6	File: ANTHEM, Count: 9
File: PROGRAMME, Count: 2	File: SHE, Count: 127	File: COLLAPSE, Count: 10
File: RUBBER, Count: 6	File: START, Count: 35	File: COMMITTEE, Count: 6
File: SENTENCE, Count: 11	File: TUESDAY, Count: 3	File: CONFERENCE, Count: 3
File: SPORTS, Count: 4	File: WALK, Count: 14	File: CONSTITUTION, Count: 4
File: STORY, Count: 37	File: WEDNESDAY, Count: 6	File: DISCUSS, Count: 4
File: STUDENT, Count: 63	File: BEAUTIFUL, Count: 47	File: ELECTION, Count: 7
File: TABLE, Count: 51	File: BORING, Count: 7	File: FLAG, Count: 3
File: THIS, Count: 184	File: BRING, Count: 34	File: HONEST, Count: 4
File: TWO, Count: 48	File: BUY, Count: 51	File: JUDGE, Count: 12
File: WISE, Count: 30	File: CLEAN, Count: 48	File: LEADER, Count: 16
File: YEAR, Count: 57	File: CLOCK, Count: 5	File: LOAN, Count: 7
File: YOU, Count: 171	File: DAWN, Count: 4	File: MEET, Count: 14
File: ABOUT, Count: 8	File: DAY, Count: 15	File: PASS, Count: 25
File: BLIND, Count: 6	File: EARLY, Count: 12	File: PRACTICE, Count: 7
File: CHAPTER, Count: 4	File: FEBRUARY, Count: 4	File: QUEUE, Count: 10
File: CULTURE, Count: 6	File: FRIDAY, Count: 11	File: RAISE, Count: 4

File: EACH, Count: 19	File: HOUSE, Count: 70	File: REFUGE, Count: 3
File: FIVE, Count: 9	File: IMMEDIATELY, Count: 4	File: SUFFER, Count: 13
File: LEARN, Count: 28	File: JANUARY, Count: 4	File: VOTE, Count: 8
File: PARENT, Count: 16	File: LION, Count: 15	File: WAR, Count: 6
File: THEM, Count: 16	File: MIDNIGHT, Count: 4	File: SOCKET, Count: 7
File: BREATH, Count: 6	File: MINUTE, Count: 2	File: UTILIZE, Count: 3
File: DISABLE, Count: 3	File: NOW, Count: 234	File: CUT, Count: 10
File: GRANDFATHER, Count: 14	File: OPEN, Count: 21	File: DUST, Count: 4
File: HEALTH, Count: 14	File: PLEASE, Count: 126	File: FEAR, Count: 12
File: MEDICINE, Count: 11	File: SEE, Count: 37	File: FLOWER, Count: 11
File: OPERATE, Count: 6	File: SEVEN, Count: 3	File: FOREST, Count: 5
File: PILL, Count: 4	File: SLEEP, Count: 35	File: GROW, Count: 11
File: SKIN, Count: 7	File: VISITORS, Count: 2	File: HOT, Count: 22
File: TAKE, Count: 27	File: WAKE, Count: 7	File: PLANT, Count: 20
File: AGO, Count: 6	File: WATCH, Count: 11	File: ROCK, Count: 5
File: AMBULANCE, Count: 4	File: AFTER, Count: 36	File: SOIL, Count: 7
File: BHANG, Count: 5	File: ANGRY, Count: 10	File: TEA, Count: 26
File: BODY, Count: 20	File: BALL, Count: 15	File: THUNDER, Count: 5
File: BROTHER, Count: 22	File: BECOME, Count: 28	File: TREE, Count: 26
File: COVER, Count: 7	File: BOX, Count: 8	File: AIR, Count: 5
File: DIE, Count: 7	File: BRIDGE, Count: 15	File: CAVE, Count: 5
File: LEG, Count: 10	File: CALL, Count: 16	File: COLD, Count: 38
File: MOUTH, Count: 5	File: CHARGER, Count: 2	File: EAST, Count: 9
File: SMOKE, Count: 11	File: COMPANY, Count: 23	File: ENTER, Count: 14
File: SNEEZE, Count: 5	File: CONNECT, Count: 10	File: HOLE, Count: 9
File: TOBACCO, Count: 5	File: DECIDE, Count: 9	File: INSIDE, Count: 8
File: AXE, Count: 8	File: DISCONNECT, Count: 9	File: LARGE, Count: 13
File: BUSH, Count: 4	File: ELECTRICITY, Count: 9	File: OCEAN, Count: 5

File: CELEBRATION, Count: 11	File: ELEVATOR, Count: 7	File: RISE, Count: 10
File: FORK, Count: 8	File: FAMILY, Count: 45	File: SNAKE, Count: 16
File: ROOM, Count: 17	File: FEEL, Count: 19	File: STAR, Count: 3
File: THIRTY, Count: 2	File: FLOOR, Count: 31	File: SUN, Count: 14
File: ACHE, Count: 3	File: FOOD, Count: 44	File: WEATHER, Count: 9
File: CHOLERA, Count: 5	File: FRIEND, Count: 32	File: ARRIVE, Count: 9
File: CORPSE, Count: 4	File: HELP, Count: 33	File: COMPETITION, Count: 11
File: FAIL, Count: 5	File: HER, Count: 91	File: EAT, Count: 43
File: FEVER, Count: 5	File: HERS, Count: 19	File: END, Count: 23
File: KIDNEY, Count: 2	File: KICK, Count: 8	File: HAND, Count: 19
File: MUMPS, Count: 2	File: KINDLY, Count: 13	File: HOUR, Count: 4
File: NORMAL, Count: 4	File: LAPTOP, Count: 9	File: LATE, Count: 14
File: OXYGEN, Count: 3	File: LEFTOVER, Count: 5	File: LATER, Count: 6
File: PERSPIRE, Count: 3	File: LIFT, Count: 7	File: MARKET, Count: 13
File: RINGWORM, Count: 3	File: MACHINE, Count: 22	File: SATURDAY, Count: 8
File: SERIOUS, Count: 14	File: NEED, Count: 61	File: SECOND, Count: 3
File: STOOL, Count: 3	File: PEEP, Count: 6	File: SUNDAY, Count: 3
File: AIRCRAFT, Count: 3	File: PHONE, Count: 22	File: SUNRISE, Count: 2
File: AIRPORT, Count: 8	File: PLAN, Count: 19	File: TEN, Count: 11
File: BUS, Count: 11	File: PRODUCT, Count: 11	File: THURSDAY, Count: 2
File: CORNER, Count: 6	File: RELEASE, Count: 9	File: TOILET, Count: 8
File: GARAGE, Count: 3	File: RELIEF, Count: 6	File: VISITOR, Count: 16
File: JOURNEY, Count: 7	File: SOCIAL MEDIA, Count: 2	File: WASH, Count: 12
File: LICENSE, Count: 6	File: SOMEONE, Count: 5	File: YET, Count: 5
File: PASSENGER, Count: 3	File: TOP, Count: 9	File: ADMIRE, Count: 2
File: PATH, Count: 5	File: TOWN, Count: 29	File: CAR, Count: 32
File: SPEED, Count: 5	File: TRASHCAN, Count: 3	File: COURAGE, Count: 5
File: TICKET, Count: 6	File: US, Count: 150	File: EXPERT, Count: 5

File: TRANSPORT, Count: 5	File: USE, Count: 74	File: FAST, Count: 16
File: WAY, Count: 8	File: WANT, Count: 74	File: GIRL, Count: 30
File: ACCIDENT, Count: 17	File: WORRY, Count: 11	File: GRADE, Count: 9
File: BICYCLE, Count: 3	File: ANIMAL, Count: 17	File: INTEREST, Count: 9
File: CART, Count: 2	File: BIG, Count: 56	File: LADY, Count: 17
File: CONDUCTOR, Count: 3	File: COMPUTER, Count: 12	File: PERFECT, Count: 8
File: FARE, Count: 2	File: DICTIONARY, Count: 6	File: PLACE, Count: 29
File: PASSPORT, Count: 5	File: EASY, Count: 14	File: SIGN, Count: 15
File: PEDAL, Count: 3	File: FIERCE, Count: 2	File: SIMPLE, Count: 13
File: SHIP, Count: 5	File: FULL, Count: 30	File: SLOWLY, Count: 3
File: TRAFFIC, Count: 3	File: HILL, Count: 4	File: WEAK, Count: 8
File: VAN, Count: 3	File: LESSO, Count: 4	File: WET, Count: 3
File: BOTH, Count: 4	File: LORRY, Count: 11	File: YOURS, Count: 33
File: CONFUSE, Count: 3	File: MAKE, Count: 54	File: BASKETBALL, Count: 9
File: DOOR, Count: 13	File: MOTORCYCLE, Count: 4	File: OFF, Count: 16
File: IDEA, Count: 11	File: NAIROBI, Count: 20	File: TURN, Count: 10
File: LENGTH, Count: 3	File: NICE, Count: 76	File: EXPECT, Count: 6
File: PRICE, Count: 4	File: PLATE, Count: 13	File: LIGHTS, Count: 4
File: REDUCE, Count: 2	File: THERE, Count: 156	File: DANCE, Count: 15
File: SUGAR, Count: 18	File: TRAIN, Count: 15	File: POLICEMAN, Count: 9
File: WIDE, Count: 5	File: WATER, Count: 45	File: NOTHING, Count: 126
File: ALARM, Count: 3	File: WHO, Count: 44	File: ANIMALS, Count: 2
File: COURT, Count: 4	File: WORD, Count: 19	File: ANTELOPE, Count: 3
File: DOUBT, Count: 3	File: BEANS, Count: 11	File: CAT, Count: 15
File: FREE, Count: 5	File: BIBLE, Count: 16	File: CLIMB, Count: 5
File: IMPOSSIBLE, Count: 12	File: BLACK, Count: 19	File: COCK, Count: 2
File: JAMES, Count: 4	File: BREAD, Count: 15	File: CROW, Count: 2
File: KNOWLEDGE, Count: 2	File: BUILDING, Count: 30	File: DANGEROUS, Count: 10

File: MEAN, Count: 3	File: BUTTER, Count: 11	File: DELICIOUS, Count: 2
File: MIND, Count: 5	File: COUNTRY, Count: 41	File: FEED, Count: 6
File: MISS, Count: 7	File: DOCTOR, Count: 40	File: GIRAFFE, Count: 3
File: NARROW, Count: 3	File: FIELD, Count: 13	File: GOAT, Count: 6
File: NEVER, Count: 3	File: FRUIT, Count: 16	File: HEN, Count: 8
File: READ, Count: 27	File: POLICE, Count: 52	File: HUMAN, Count: 2
File: SOLVE, Count: 6	File: RICE, Count: 11	File: HYENA, Count: 6
File: SOMETHING, Count: 2	File: SHEEP, Count: 18	File: LIKE, Count: 43
File: SUCCEED, Count: 2	File: SISTER, Count: 24	File: LIVE, Count: 16
File: THING, Count: 8	File: SOUP, Count: 18	File: MEAT, Count: 18
File: TO, Count: 3	File: SWEET, Count: 31	File: MONKEY, Count: 4
File: WHY, Count: 37	File: TELEPHONE, Count: 13	File: OTHER, Count: 22
File: ALLOW, Count: 5	File: UGANDA, Count: 12	File: TALL, Count: 14
File: ANNUAL, Count: 2	File: ZERO, Count: 78	File: TWENTY, Count: 4
File: COMPLETE, Count: 4	File: CHURCH, Count: 35	File: WILD, Count: 5
File: CONSTRUCTION, Count: 3	File: JESUS, Count: 32	File: ASCEND, Count: 2
File: CONTINUE, Count: 8	File: KENYA, Count: 30	File: BELIEVE, Count: 4
File: DEFY, Count: 3	File: LAMB, Count: 2	File: CHRIST, Count: 11
File: DINNER, Count: 4	File: LOVE, Count: 85	File: GLORY, Count: 4
File: EXAGGARATE, Count: 2	File: MOSQUE, Count: 15	File: KILL, Count: 14
File: HAPPEN, Count: 13	File: MOTHER, Count: 41	File: ONLY, Count: 18
File: INTRODUCE, Count: 4	File: MUSLIM, Count: 9	File: PERFORM, Count: 2
File: INVITE, Count: 5	File: NAME, Count: 39	File: PERSON, Count: 27
File: LACE, Count: 3	File: NATIONAL, Count: 17	File: WED, Count: 8
File: ORDER, Count: 3	File: NEIGHBOUR, Count: 2	File: ARREST, Count: 13
File: PROGRESS, Count: 2	File: OFFERING, Count: 3	File: ASSEMBLY, Count: 4
File: RESEARCH, Count: 6	File: PASTOR, Count: 10	File: AWARD, Count: 4
File: ROLE, Count: 4	File: POWER, Count: 10	File: BEST, Count: 23

File: SHOE, Count: 9	File: PRAYER, Count: 5	File: BREAK, Count: 28
File: TASK, Count: 5	File: PRIEST, Count: 12	File: CAN, Count: 16
File: THINK, Count: 10	File: SACRIFICE, Count: 3	File: CATCH, Count: 9
File: INSTRUCT, Count: 3	File: SICK, Count: 37	File: CLOTH, Count: 17
File: MOST, Count: 2	File: ABRAHAM, Count: 7	File: CUP, Count: 21
File: NEAR, Count: 13	File: CHRISTIAN, Count: 11	File: EGG, Count: 19
File: PARTY, Count: 15	File: DAVID, Count: 11	File: EVERY, Count: 7
File: SHIRT, Count: 11	File: DISCIPLE, Count: 11	File: GLUE, Count: 7
File: TROUSER, Count: 6	File: EASTER, Count: 6	File: HANG, Count: 10
File: AWAKE, Count: 4	File: FAITH, Count: 6	File: HOLD, Count: 6
File: BAKE, Count: 2	File: FATHER, Count: 69	File: IF, Count: 21
File: BEHAVIOUR, Count: 6	File: GOD, Count: 23	File: MARCH, Count: 4
File: CLOTHE, Count: 3	File: GOLIATH, Count: 6	File: MATCH, Count: 8
File: GIRLS, Count: 3	File: HEAVEN, Count: 7	File: ON, Count: 24
File: HEADACHE, Count: 3	File: HOLLY, Count: 2	File: PAPER, Count: 25
File: LEARNER, Count: 11	File: IDOL, Count: 10	File: PICK, Count: 9
File: LEAVE, Count: 12	File: MIRACLE, Count: 6	File: POINT, Count: 4
File: PALE, Count: 2	File: MOSES, Count: 4	File: POSSIBLE, Count: 35
File: PREGNANT, Count: 5	File: ONE, Count: 31	File: PUT, Count: 8
File: TIRED, Count: 7	File: SOON, Count: 22	File: SCOUT, Count: 6
File: ABSTRACT, Count: 2	File: TRUE, Count: 81	File: SHAPE, Count: 6
File: ACCEPT, Count: 19	File: TWELVE, Count: 9	File: SMALL, Count: 16
File: ACROSS, Count: 4	File: WORSHIP, Count: 10	File: SON, Count: 18
File: ADVOCATE, Count: 3	File: BISHOP, Count: 6	File: STICK, Count: 9
File: AIRPLANE, Count: 2	File: ELDER, Count: 4	File: STUDENTS, Count: 5
File: ALCOHOL, Count: 2	File: FASTING, Count: 5	File: SUNSHINE, Count: 8
File: ANGEL, Count: 2	File: OBEY, Count: 14	File: TIE, Count: 14
File: APPEAR, Count: 4	File: PRAY, Count: 26	File: TRY, Count: 9

File: APPLE, Count: 3	File: PSALMS, Count: 6	File: VASE, Count: 3
File: APPLY, Count: 3	File: RELIGION, Count: 2	File: WAIT, Count: 11
File: AROUND, Count: 3	File: SATAN, Count: 3	File: CAUSE, Count: 11
File: ATHLETE, Count: 4	File: SIN, Count: 6	File: DECAY, Count: 3
File: BACKGROUND, Count: 3	File: TRUST, Count: 5	File: EMPTY, Count: 2
File: BACKWARD, Count: 3	File: WEEK, Count: 24	File: HEAL, Count: 6
File: BAPTIZE, Count: 4	File: WORLD, Count: 8	File: HIM, Count: 32
File: BASIC, Count: 3	File: HUMBLE, Count: 16	File: MALARIA, Count: 6
File: BASIN, Count: 2	File: MAN, Count: 96	File: MEASURE, Count: 2
File: BED, Count: 7	File: REFUGEE, Count: 11	File: NET, Count: 2
File: BEE, Count: 6	File: BABY, Count: 67	File: PATIENT, Count: 9
File: BEHIND, Count: 7	File: BLANKET, Count: 11	File: STOMACH, Count: 5
File: BELOW, Count: 2	File: CHAIR, Count: 33	File: TEETH, Count: 7
File: BENCH, Count: 2	File: CHART, Count: 7	File: THROW, Count: 2
File: BIRD, Count: 2	File: COMFORTABLE, Count: 10	File: TOOTHPASTE, Count: 3
File: BITE, Count: 5	File: DIRTY, Count: 26	File: TREAT, Count: 6
File: BLADE, Count: 2	File: FOOTBALL, Count: 13	File: VOMIT, Count: 5
File: BLOOD, Count: 6	File: FRIDGE, Count: 7	File: WOUND, Count: 2
File: BLUE, Count: 13	File: HAPPY, Count: 13	File: ALOT, Count: 35
File: BOIL, Count: 4	File: HEARING AID, Count: 7	File: DEBTS, Count: 2
File: BOOK BOOK, Count: 3	File: HEAVY, Count: 22	File: DOING, Count: 2
File: BRANCH, Count: 2	File: JAIL, Count: 10	File: EMPOWERMENT, Count: 2
File: BUILT, Count: 4	File: JIKO, Count: 8	File: RECIEVE, Count: 3
File: CAKE, Count: 10	File: LOST, Count: 31	File: ADVICE, Count: 4
File: CEREMONY, Count: 3	File: PENCIL, Count: 18	File: BUILD, Count: 8
File: CHARGE, Count: 2	File: STEAL, Count: 27	File: CHIEF, Count: 7
File: CHIPS, Count: 5	File: THOSE, Count: 28	File: COMPETE, Count: 3
File: COACH, Count: 3	File: THREE, Count: 27	File: COVID, Count: 3

File: COAT, Count: 3<br><br>File: COMMANDMENT, Count: 3<br><br>File: COMMUNICATION, Count: 10<br><br>File: COMMUNITY, Count: 4<br><br>File: COMPOUND, Count: 5<br><br>File: CONCEPT, Count: 2<br><br>File: CONGRATULATE, Count: 2<br><br>File: COW COW, Count: 2<br><br>File: DAUGHTER, Count: 9<br><br>File: DEATH, Count: 5<br><br>File: DEEP, Count: 7<br><br>File: DELETE, Count: 2<br><br>File: DESERT, Count: 2<br><br>File: DIARRHOEA, Count: 2<br><br>File: DIP, Count: 2<br><br>File: DISAPPEAR, Count: 4<br><br>File: DISCARD, Count: 3<br><br>File: DISH, Count: 2<br><br>File: DOMESTIC, Count: 2<br><br>File: DRAINAGE, Count: 2<br><br>File: DRINK, Count: 8<br><br>File: DURING, Count: 9<br><br>File: DUSTBIN, Count: 2<br><br>File: EARPHONE, Count: 2<br><br>File: ELEPHANT, Count: 6<br><br>File: EVERYTHING, Count: 4<br><br>File: EVIL, Count: 2<br><br>File: EXERCISE, Count: 11	File: UMBRELLA, Count: 7<br><br>File: THERMOMETER, Count: 8<br><br>File: WHICH, Count: 14<br><br>File: A LOT, Count: 64<br><br>File: AGENDA, Count: 5<br><br>File: ANYTHING, Count: 6<br><br>File: BARGAIN, Count: 8<br><br>File: CURRENTLY, Count: 3<br><br>File: CUSTOMER, Count: 4<br><br>File: DEBT, Count: 5<br><br>File: ECONOMY, Count: 13<br><br>File: EMPLOYMENT, Count: 4<br><br>File: FUNDS, Count: 3<br><br>File: CHARCOAL, Count: 3<br><br>File: ACTRESS, Count: 2<br><br>File: BLESS, Count: 2<br><br>File: FUTUTE, Count: 2<br><br>File: LORD, Count: 2<br><br>File: TEMPLE, Count: 2<br><br>File: PROCESS, Count: 4<br><br>File: DELIVER, Count: 2<br><br>File: STAIR, Count: 2<br><br>File: OWN, Count: 2<br><br>File: NOT YET, Count: 5<br><br>File: CITIZEN, Count: 4<br><br>File: DECISION, Count: 5<br><br>File: EQUALITY, Count: 2<br><br>File: PARDON, Count: 2	File: DEPARTMENT, Count: 3<br><br>File: DIFFICULT, Count: 20<br><br>File: DISMISS, Count: 3<br><br>File: ENGINEER, Count: 3<br><br>File: IGNORE, Count: 5<br><br>File: INDUSTRY, Count: 5<br><br>File: INSTITUTION, Count: 7<br><br>File: LUGGAGE, Count: 7<br><br>File: MANAGE, Count: 7<br><br>File: RESIGN, Count: 4<br><br>File: RESPONSIBILITY, Count: 11<br><br>File: SPREAD, Count: 10<br><br>File: STRUCTURE, Count: 3<br><br>File: SUGGESTION, Count: 3<br><br>File: SUPERVISOR, Count: 5<br><br>File: SUPPORT, Count: 18<br><br>File: TEACH, Count: 20<br><br>File: AGAINST, Count: 4<br><br>File: K-A-M-A-U, Count: 2<br><br>File: APPOINT, Count: 5<br><br>File: CONTROL, Count: 10<br><br>File: COUNSEL, Count: 2<br><br>File: FACTORY, Count: 6<br><br>File: HAS, Count: 24<br><br>File: HOW, Count: 10<br><br>File: NEEDS, Count: 2<br><br>File: SELF, Count: 13<br><br>File: SUPERVISE, Count: 2

File: EXPLAIN, Count: 8	File: RIGHTS, Count: 2	File: UNDER, Count: 16
File: EYE, Count: 2	File: VOTER, Count: 2	File: WOMAN, Count: 24
File: FAVOURITE, Count: 2	File: CAMPAIN, Count: 2	File: BRAILLE, Count: 5
File: FIRST, Count: 6	File: FARMING, Count: 2	File: EXAM, Count: 22
File: FLOOD, Count: 4	File: KANU, Count: 2	File: EXAMINATION, Count: 4
File: FLY, Count: 3	File: CONCEAL, Count: 2	File: EXPERIENCE, Count: 9
File: FORWARD, Count: 2	File: GREEDY, Count: 3	File: HARD, Count: 48
File: FROG, Count: 3	File: IGNORANT, Count: 3	File: INTELLIGENT, Count: 2
File: FRONT, Count: 4	File: SELF-CONTROL, Count: 2	File: NOUN, Count: 8
File: FUEL, Count: 3	File: ARMY, Count: 3	File: OUT, Count: 8
File: GAME, Count: 9	File: BILL, Count: 2	File: RESULTS, Count: 7
File: GLAD, Count: 4	File: EUROPEAN, Count: 2	File: SUCCESS, Count: 11
File: GOLD, Count: 3	File: EXCELLENT, Count: 2	File: SYLLABUS, Count: 5
File: GRAZE, Count: 2	File: PALIAMENT, Count: 3	File: TECHNOLOGY, Count: 7
File: GUILT, Count: 2	File: PROPAGANDA, Count: 2	File: TEMPERATURE, Count: 4
File: HAMMER, Count: 2	File: REPRESENT, Count: 3	File: TERM, Count: 10
File: HARE, Count: 6	File: SCRAMBLE, Count: 3	File: UNDERSTAND, Count: 15
File: HARSH, Count: 6	File: EMBASSY, Count: 2	File: AFTERNOON, Count: 7
File: HARVEST, Count: 5	File: LEGAL, Count: 5	File: STARS, Count: 2
File: HERE, Count: 19	File: MAUMAU, Count: 4	File: APRIL, Count: 2
File: HIT, Count: 6	File: SENATE, Count: 5	File: AUGUST, Count: 2
File: HORIZONTAL, Count: 2	File: SERVE, Count: 7	File: BORN, Count: 5
File: INJURE, Count: 4	File: DORMITORY, Count: 2	File: CALENDER, Count: 4
File: INVOLVE, Count: 4	File: OPTIONAL, Count: 4	File: CELEBRATE, Count: 8
File: IRON, Count: 2	File: DREAM, Count: 3	File: DECEMBER, Count: 3
File: IT, Count: 7	File: FORGET, Count: 3	File: JULY, Count: 2
File: J-O-H-N, Count: 4	File: BUNGOMA, Count: 2	File: M-A-R-Y, Count: 7
File: JOIN, Count: 11	File: BUSIA, Count: 3	File: MAY, Count: 3

File: JUMP, Count: 3	File: GILGIL, Count: 2	File: MUSIC, Count: 11
File: KEY, Count: 6	File: KAMBUI, Count: 2	File: OCTOBER, Count: 2
File: KITCHEN, Count: 6	File: KERICHO, Count: 2	File: PLOUGH, Count: 2
File: KNEE, Count: 2	File: KUJA, Count: 2	File: T-O-M, Count: 4
File: KNEEL, Count: 6	File: LAMU, Count: 2	File: TRAVEL, Count: 29
File: KNIFE, Count: 6	File: MALINDI, Count: 2	File: ADULT, Count: 11
File: LADDER, Count: 2	File: MOLO, Count: 2	File: BEARD, Count: 4
File: LEATHER, Count: 2	File: POTATO, Count: 4	File: BRUSH, Count: 6
File: LINE, Count: 4	File: THIKA, Count: 2	File: DOG, Count: 14
File: LIPSTICK, Count: 2	File: TUMUTUMU, Count: 2	File: EARTH, Count: 5
File: LOCAL, Count: 2	File: VEHICLE, Count: 2	File: IN, Count: 38
File: LOG, Count: 2	File: COCONUT, Count: 3	File: LAND, Count: 17
File: M-A-R-C-H, Count: 2	File: LONELY, Count: 3	File: LITTLE, Count: 6
File: MARY, Count: 4	File: BEE BEE, Count: 2	File: LONG, Count: 25
File: MORE, Count: 10	File: MILLET, Count: 2	File: MOON, Count: 7
File: MULTIPLY, Count: 2	File: PAWPAW, Count: 2	File: PAINT, Count: 9
File: MY, Count: 6	File: POPCORN, Count: 2	File: ROUND, Count: 6
File: NAIL, Count: 3	File: WORKER WORKER, Count: 3	File: SHOES, Count: 13
File: NORTHING, Count: 2	File: TIE (CLOTH), Count: 2	File: SMELL, Count: 21
File: OBJECTIVE, Count: 4	File: PETER, Count: 4	File: SOCKS, Count: 11
File: OIL, Count: 9	File: 5, Count: 2	File: TOOTHBRUSH, Count: 7
File: OPPOSITE, Count: 2	File: BLOUSE, Count: 3	File: WALL, Count: 19
File: ORANGE, Count: 7	File: BURRY, Count: 3	File: YOUTH, Count: 9
File: OUTSIDE, Count: 6	File: LABORATORY, Count: 2	File: CHEAP, Count: 4
File: OVERTAKE, Count: 2	File: M-O-D-E-L, Count: 2	File: COLLEGE, Count: 3
File: PANGA, Count: 4	File: MICROSCOPE, Count: 2	File: FEE, Count: 3
File: PARALLEL, Count: 2	File: PHOTOGRAPH, Count: 2	File: FINE, Count: 5
File: PAUL, Count: 2	File: UNDERGROUND, Count: 2	File: LIFE, Count: 7

File: PERFUME, Count: 2	File: MAYBE, Count: 2	File: REPAIR, Count: 5
File: PERSUADE, Count: 4	File: TONIGHT, Count: 4	File: RICH, Count: 12
File: PICTURE, Count: 11	File: BULL, Count: 2	File: SECONDARY, Count: 2
File: PIECE, Count: 4	File: LITRE, Count: 2	File: SUM, Count: 9
File: POLLUTE, Count: 2	File: CORRECT, Count: 7	File: BLEED, Count: 5
File: POOR, Count: 19	File: EXCITE, Count: 3	File: COOK, Count: 17
File: POSITION, Count: 5	File: TOOTH, Count: 2	File: DRUM, Count: 6
File: PRIMARY, Count: 5	File: CORRUPT, Count: 2	File: ELDORET, Count: 6
File: PRINCIPAL, Count: 9	File: DEAFBLIND, Count: 3	File: FISH, Count: 10
File: PRINCIPLE, Count: 2	File: EVERYDAY, Count: 3	File: HEAD, Count: 12
File: PRODUCE, Count: 6	File: ONLINE, Count: 4	File: KING, Count: 12
File: RAT, Count: 7	File: FINAL, Count: 2	File: LIZARD, Count: 6
File: RECTANGULAR, Count: 4	File: ADAM, Count: 3	File: MAIZE, Count: 8
File: RED, Count: 6	File: IRON BOX, Count: 3	File: MUSLIMS, Count: 2
File: REFEREE, Count: 3	File: PAIL, Count: 2	File: TASTE, Count: 3
File: RELATIVE, Count: 5	File: RULER, Count: 3	File: ACHIEVE, Count: 7
File: RELAX, Count: 7	File: GAVE, Count: 2	File: EDUCATION, Count: 6
File: REMAIN, Count: 3	File: SALT, Count: 2	File: HISTORY, Count: 3
File: RESTORE, Count: 2	File: ELAGANT, Count: 3	File: INFORMATION, Count: 9
File: RESULT, Count: 8	File: CRAZY, Count: 3	File: INTERESTING, Count: 15
File: REVENGE, Count: 2	File: CRUTCHES, Count: 2	File: SCIENCE, Count: 3
File: RUN, Count: 7	File: DETORIORATE, Count: 2	File: SKILL, Count: 8
File: S-H-A-R-P, Count: 2	File: MENSTRATION, Count: 2	File: ART, Count: 2
File: SAFE, Count: 5	File: PNEUMONIA, Count: 3	File: CAREER, Count: 3
File: SAND, Count: 2	File: CHAIN, Count: 2	File: COURSE, Count: 7
File: SCISSORS, Count: 3	File: COUNTY, Count: 3	File: DEVELOP, Count: 8
File: SELECT, Count: 3	File: GYM, Count: 2	File: EXPENSIVE, Count: 11
File: SERVANT, Count: 2	File: CORRUPTION, Count: 2	File: FROM, Count: 2

File: SHAKE, Count: 8	File: 1963, Count: 6	File: HANDSOME, Count: 6
File: SHARE, Count: 13	File: PRINCESS, Count: 3	File: JOY, Count: 7
File: SHARP, Count: 4	File: BEER, Count: 3	File: LAW, Count: 6
File: SHARPEN, Count: 5	File: TANZANIA, Count: 2	File: PRACTICAL, Count: 2
File: SIDE, Count: 2	File: ECONOMIC, Count: 2	File: SING, Count: 15
File: SIGNPOST, Count: 2	File: DONKEY, Count: 4	File: ANY, Count: 7
File: SKILLS, Count: 3	File: HIPPOPOTAMUS, Count: 3	File: ASK, Count: 9
File: SLAUGHTER, Count: 4	File: HORSE, Count: 5	File: BAD, Count: 27
File: SLOW, Count: 5	File: TORTOISE, Count: 2	File: CARRY, Count: 22
File: SOAP, Count: 9	File: ZEBRA, Count: 3	File: CHOOSE, Count: 3
File: SORRY, Count: 8	File: SEEK, Count: 2	File: CLAP, Count: 5
File: SPOON, Count: 10	File: SHOPKEEPER, Count: 2	File: FACE, Count: 9
File: STADIUM, Count: 2	File: BUFFALO, Count: 2	File: HIDE, Count: 10
File: STOP, Count: 18	File: CHEETER, Count: 2	File: K-E-V-I-N, Count: 2
File: STRING, Count: 4	File: QUEEN, Count: 3	File: SEND, Count: 8
File: STROLL, Count: 2	File: MANY MANY, Count: 3	File: SHOULDER, Count: 6
File: STRUGGLE, Count: 4	File: SEAT SEAT, Count: 2	File: WAKE UP, Count: 4
File: SURRENDER, Count: 2	File: COMMA, Count: 2	File: WEDDING, Count: 10
File: SURVIVE, Count: 2	File: ITEM, Count: 2	File: BUT, Count: 4
File: SWALLOW, Count: 3	File: SEMICOLON, Count: 2	File: EGGS, Count: 2
File: SWEEP, Count: 2	File: CAMP, Count: 2	File: GREAT, Count: 7
File: SWIM, Count: 7	File: EXPIRE, Count: 2	File: LOOK, Count: 15
File: SWING, Count: 2	File: BATHE, Count: 3	File: NO, Count: 5
File: THEY, Count: 21	File: CHAPATI, Count: 2	File: SACK, Count: 2
File: THREAD, Count: 2	File: CHICKEN, Count: 2	File: PAINTING, Count: 8
File: THROUGH, Count: 4	File: COFFEE, Count: 3	File: WHEN, Count: 13
File: TOMATO, Count: 2	File: CHICKENPOX, Count: 2	File: DIRT, Count: 4
File: TOWARDS, Count: 2	File: MEDICAL, Count: 2	File: MILLITARY, Count: 4

File: TRADITION, Count: 2	File: CONDOM, Count: 2	File: SOLIDIER, Count: 4
File: TROUBLE, Count: 4	File: DIABETES, Count: 2	File: BASKET BALL, Count: 2
File: TUNNEL, Count: 2	File: EPIDEMIC, Count: 2	File: CAREFULL, Count: 2
File: TWIST, Count: 3	File: HERB, Count: 2	File: KAMAU, Count: 2
File: UGLY, Count: 4	File: IMPOTENT, Count: 2	File: KIND, Count: 7
File: UNIFORM, Count: 3	File: NOSEBLEED, Count: 2	File: SOCIAL, Count: 7
File: UNITY, Count: 4	File: TRADITIONAL, Count: 2	File: TRASH, Count: 2
File: UP, Count: 15	File: URINATE, Count: 2	File: AFRICA, Count: 10
File: VERSE, Count: 5	File: X-RAY, Count: 2	File: ARE, Count: 9
File: VILLAGE, Count: 13	File: BEHAVE, Count: 2	File: CLEVER, Count: 12
File: WEAR, Count: 12	File: RIDE, Count: 3	File: KISWAHILI, Count: 4
File: WHAT, Count: 27	File: TRAFFIC LIGHT, Count: 2	File: LUO, Count: 3
File: WHERE, Count: 18	File: JACKET, Count: 2	File: WIFE, Count: 6
File: WHISTLE, Count: 3	File: KNIT, Count: 2	File: BUSINESS, Count: 23
File: WINDOW, Count: 5	File: MATATU, Count: 3	File: CHANGE, Count: 16
File: WOOD, Count: 7	File: PANTY, Count: 2	File: EXPLANATION, Count: 3
File: YOUNG, Count: 4	File: SEW, Count: 2	File: MARK, Count: 4
File: ACTION, Count: 5	File: TAILORING, Count: 2	File: PROGRAM, Count: 5
File: BE, Count: 9	File: TAXI, Count: 3	File: FULLSTOP, Count: 3
File: BOTTLE, Count: 4	File: ZIP, Count: 2	File: GUESS, Count: 3
File: BREAST, Count: 2	File: DEODORANT, Count: 2	File: LESSON, Count: 6
File: HATE, Count: 31	File: EARRING, Count: 2	File: NEWSPAPER, Count: 6
File: LOUD, Count: 7	File: FLU, Count: 2	File: OLD, Count: 30
File: RIGID, Count: 2	File: HEADSCARF, Count: 2	File: TEXT, Count: 3
File: SPEAK, Count: 8	File: SKIRT, Count: 2	File: TEXTBOOK, Count: 3
File: STAGE, Count: 2	File: TROUSERS, Count: 4	File: WIN, Count: 12
File: BETTER, Count: 6	File: BOWTIE, Count: 2	File: ADJECTIVE, Count: 5
File: BLAZER, Count: 2	File: BUIBUI, Count: 2	File: AUDIOLOGY, Count: 2

File: BOYS, Count: 2	File: MITUMBA, Count: 2	File: CURRICULUM, Count: 2
File: SMART, Count: 8	File: PAJAMA, Count: 2	File: DRAMA, Count: 2
File: STRONG, Count: 13	File: POCKET, Count: 2	File: EIGHT, Count: 7
File: WHITE, Count: 10	File: SUPERMARKET, Count: 2	File: FESTIVAL, Count: 2
File: WISH, Count: 5	File: WALLET, Count: 2	File: FIFTY, Count: 2
File: TYPE, Count: 10	File: EVERLASTING, Count: 2	File: GRAMMAR, Count: 2
File: CHINESE, Count: 2	File: WEEP, Count: 2	File: IDIOT, Count: 5
File: CROSS, Count: 5	File: CURIOUS, Count: 2	File: POEM, Count: 6
File: DATE, Count: 11	File: DANGER, Count: 2	File: PROJECT, Count: 9
File: FIANCEE, Count: 2	File: TRUTH, Count: 3	File: PROPOSAL, Count: 4
File: PARTNER, Count: 3	File: CARELESS, Count: 2	File: STUDY, Count: 8
File: RELATE, Count: 2	File: 12, Count: 2	File: WORDS, Count: 2
File: GREET, Count: 3	File: UNTIL, Count: 2	File: BUTCHER, Count: 5
File: HOBBY, Count: 3	File: CARELESSNESS, Count: 2	File: DONOR, Count: 5
File: INTREST, Count: 2	File: WINE, Count: 2	File: FILL, Count: 2
File: POST, Count: 2	File: VEST, Count: 2	File: FIRE, Count: 8
File: REGISTER, Count: 8	File: RADIO, Count: 2	File: GOVERNOR, Count: 4
File: RING, Count: 12	File: WHEELCHAIR, Count: 2	File: INSTRUCTION, Count: 2
File: SECRET, Count: 3	File: COUSIN, Count: 5	File: MATHEMATICS, Count: 6
File: VIDEO, Count: 3	File: MARRY, Count: 6	File: RECEIPT, Count: 6
File: WHISPER, Count: 2	File: PAIN, Count: 6	File: SHOP, Count: 9
File: AFFECT, Count: 4	File: RESPECT, Count: 12	File: STATION, Count: 2
File: ANNOUNCE, Count: 2	File: SILENT, Count: 2	File: BATH, Count: 2
File: BRIEF, Count: 2	File: COOKING, Count: 2	File: EMOTIONAL, Count: 4
File: FRIENDS, Count: 4	File: GAS, Count: 4	File: PREFER, Count: 3
File: INTERNET, Count: 3	File: VOLUME, Count: 2	File: REJOICE, Count: 4
File: JOSEPH, Count: 2	File: ICE, Count: 3	File: REST, Count: 6
File: PARK, Count: 6	File: ACTIVE, Count: 2	File: SULK, Count: 4

File: VOICE, Count: 2	File: CAPTAIN, Count: 2	File: UGALI, Count: 19
File: AUNT, Count: 9	File: DRIVE, Count: 16	File: BOTHER, Count: 4
File: BIRTH, Count: 10	File: TOGETHER, Count: 9	File: CHARACTER, Count: 3
File: BRIDE, Count: 5	File: VIEW, Count: 2	File: FANTASTIC, Count: 4
File: INTERPRET, Count: 2	File: DOTS, Count: 2	File: FUNNY, Count: 4
File: MAINTAIN, Count: 4	File: DRAW, Count: 9	File: JOKE, Count: 4
File: MASENO, Count: 3	File: EXTEND, Count: 2	File: NERVOUS, Count: 3
File: ROTATE, Count: 2	File: FOREHEAD, Count: 2	File: NYAMACHOMA, Count: 2
File: SALUTE, Count: 6	File: NECK, Count: 2	File: OMENA, Count: 2
File: WRIST, Count: 2	File: THUMB, Count: 2	File: SAUSAGE, Count: 3
File: HOMABAY, Count: 2	File: ZIGZAG, Count: 2	File: SUGARCANE, Count: 2
File: TWIN, Count: 4	File: BLESSING, Count: 2	File: SWEAT, Count: 4
File: CASSAVA, Count: 4	File: COMPARE, Count: 3	File: TEAR, Count: 2
File: EXPLODE, Count: 2	File: CONTRAST, Count: 2	File: APPOINTMENT, Count: 3
File: FARM, Count: 11	File: FOCUS, Count: 3	File: AVOID, Count: 5
File: MEMORY, Count: 3	File: HEALTHY, Count: 4	File: CANCEL, Count: 3
File: BOREHOLE, Count: 2	File: CANDLE, Count: 4	File: DISTURB, Count: 3
File: EQUAL, Count: 4	File: CARPET, Count: 3	File: FALL, Count: 8
File: ONION, Count: 3	File: CURTAIN, Count: 2	File: MATHS, Count: 3
File: PROVIDE, Count: 2	File: FENCE, Count: 3	File: MISTAKE, Count: 5
File: ROLL, Count: 3	File: GATE, Count: 6	File: NURSE, Count: 7
File: SERVICE, Count: 10	File: IRON SHEET, Count: 2	File: REFUSE, Count: 5
File: SIBLING, Count: 2	File: FOLLOW, Count: 7	File: SHOUT, Count: 5
File: THOUSAND, Count: 2	File: INCLUDE, Count: 3	File: STUPID, Count: 4
File: TISSUE, Count: 2	File: SHORT, Count: 10	File: WARN, Count: 8
File: BAPTISE, Count: 2	File: SICKNESS, Count: 2	File: WASTE, Count: 2
File: BIRTHDAY, Count: 5	File: SPRAY, Count: 3	File: HANDS, Count: 2
File: BRAVE, Count: 2	File: STAY, Count: 7	File: INTERVIEW, Count: 6

File: ENJOY, Count: 4	File: SUPRISE, Count: 2	File: IS, Count: 4
File: GIVING, Count: 2	File: TAIL, Count: 4	File: MARVELOUS, Count: 2
File: GUEST, Count: 4	File: BUCKET, Count: 2	File: NUMBER, Count: 4
File: HALL, Count: 7	File: COLLECT, Count: 7	File: OF, Count: 7
File: PRIZE, Count: 4	File: MIRROR, Count: 2	File: PART, Count: 3
File: ANSWER, Count: 7	File: PIPE, Count: 4	File: PUNCH, Count: 3
File: BEHAVIOR, Count: 7	File: NYANG'OMA, Count: 2	File: SAY, Count: 6
File: BORROW, Count: 7	File: REMEMBER, Count: 12	File: BROAD, Count: 2
File: EXCUSE, Count: 3	File: CRY, Count: 6	File: DESIRE, Count: 6
File: GOODBYE, Count: 2	File: HEAR, Count: 4	File: FAR, Count: 8
File: ADDRESS, Count: 4	File: HINDU, Count: 2	File: OPRESS, Count: 4
File: BUSY, Count: 7	File: INFRONT, Count: 3	File: SMILE, Count: 5
File: EMAIL, Count: 4	File: ME , Count: 2	File: STIFF, Count: 2
File: MOVIE, Count: 2	File: ENOUGH, Count: 13	File: WHEEL, Count: 2
File: PUPIL, Count: 2	File: PROVERB, Count: 2	File: CATTLE, Count: 2
File: BITTER, Count: 8	File: FAINT, Count: 2	File: CHEW, Count: 4
File: HAIR, Count: 3	File: FAKE, Count: 2	File: CROP, Count: 3
File: S-T-I-C-K, Count: 2	File: HELPFUL, Count: 2	File: ISOLATE, Count: 2
File: SEASON, Count: 2	File: DRUG, Count: 5	File: LEND, Count: 5
File: SHAVE, Count: 4	File: GLASS, Count: 6	File: PUNISH, Count: 7
File: SHOW, Count: 3	File: SODA, Count: 2	File: FAT, Count: 11
File: CLEAR, Count: 7	File: STRAIGHT, Count: 2	File: FOUR, Count: 5
File: DISAPPOINT, Count: 5	File: ENCOURAGE, Count: 2	File: LEFT, Count: 2
File: FRIENDSHIP, Count: 3	File: STRESS, Count: 3	File: WHEELBARROW, Count: 3
File: POT, Count: 3	File: VOLUNTEER, Count: 3	File: BASEMENT, Count: 2
File: PRESENT, Count: 5	File: WITH, Count: 3	
File: SILENCE, Count: 4	File: WITHOUT, Count: 3	File: ALMOST, Count: 3
File: 66, Count: 2	File: MANGO, Count: 6	File: CAP, Count: 3

File: BRIBE, Count: 4	File: ADJUST, Count: 3	File: DESCRIBE, Count: 4
File: CLOUD, Count: 3	File: BREAKFAST, Count: 2	File: GREEN, Count: 6
File: CRUCIFY, Count: 3	File: FRENCH, Count: 5	File: LAZY, Count: 8
File: FALSE, Count: 3	File: KNOW, Count: 19	File: ROBE, Count: 4
File: BUDGET, Count: 6	File: LEAF, Count: 3	File: SHAWL, Count: 2
File: EMPLOYER, Count: 3	File: MATCHBOX, Count: 2	File: SNOW, Count: 3
File: FIGHT, Count: 7	File: MESSAGE, Count: 2	File: WRAP, Count: 4
File: MEMBER, Count: 15	File: NEITHER, Count: 3	File: A-C-C-O-U-N-T, Count: 2
File: PREACH, Count: 10	File: READY, Count: 12	File: A-R-R-E-S-T, Count: 2
File: WORKSHOP, Count: 3	File: SEARCH, Count: 6	File: ACCOUNTANT, Count: 5
File: A-T-T-E-M-P-T, Count: 2	File: SEAT BELT, Count: 3	File: BETWEEN, Count: 2
File: ATTITUDE, Count: 2	File: UNLESS, Count: 2	File: EDITOR, Count: 5
File: B-R-I-A-N, Count: 2	File: WEALTH, Count: 4	File: HEADMASTER, Count: 4
File: BOARD, Count: 12	File: ACTIVITY, Count: 6	File: HEADQUARTER, Count: 2
File: DIRECT, Count: 4	File: BANANA, Count: 4	File: LABOUR, Count: 8
File: DUTY, Count: 2	File: BRIGHT, Count: 8	File: R-O-B-B-E-R, Count: 2
File: O-M-O-L-L-O, Count: 2	File: DIRECTOR, Count: 2	File: ACCUSE, Count: 2
File: P-R-O-P-O-S-A-L, Count: 2	File: INNOCENT, Count: 5	File: ACT, Count: 2
File: R-E-S-I-G-N, Count: 3	File: SELL, Count: 5	File: ACTING, Count: 2
File: RESTAURANT, Count: 3	File: SHINE, Count: 2	File: ALONE, Count: 2
File: WRITER, Count: 4	File: SPLIT, Count: 3	File: BETRAY, Count: 4
File: ADVISE, Count: 5	File: CAMERA, Count: 5	File: DOWN, Count: 2
File: GRACE, Count: 2	File: DISEASE, Count: 5	File: FORGIVE, Count: 4
File: SITUATION, Count: 4	File: VOLLEYBALL, Count: 2	File: J-A-M-E-S, Count: 3
File: ZIRO, Count: 18	File: CONFUSED, Count: 2	File: J-U-D-A-S, Count: 2
File: CHAIRMAN, Count: 3	File: BOTTOM, Count: 2	File: LANDLORD, Count: 3
File: KNIGHT, Count: 3	File: BOY THAT, Count: 2	File: LAST, Count: 24
File: PEACE, Count: 4	File: VERY, Count: 3	File: MODEL, Count: 2

File: SONG, Count: 4	File: COLOR, Count: 2	File: MURDER, Count: 6
File: VISION, Count: 2	File: EAR, Count: 2	File: S-I-T-E, Count: 2
File: ABSTAIN, Count: 2	File: UNITE, Count: 2	File: STONE, Count: 5
File: CURE, Count: 5	File: MINISTER, Count: 3	File: DIVE, Count: 3
File: HONOUR, Count: 3	File: DEAD, Count: 6	File: GRAB, Count: 3
File: INJECTION, Count: 2	File: FREEDOM, Count: 2	File: HIMSELF, Count: 2
File: METAL, Count: 2	File: MYSELF, Count: 2	File: INVADE, Count: 6
File: DRIVER, Count: 6	File: FRY, Count: 2	File: JUICE, Count: 5
File: KEEN, Count: 2	File: TOMMORROW, Count: 2	File: KISUMU, Count: 7
File: POOL, Count: 2	File: CONTRIBUTE, Count: 2	File: OFFICIATE, Count: 2
File: STRICT, Count: 2	File: FUNERAL, Count: 2	File: SEA, Count: 3
File: ANNOINT, Count: 2	File: FLAT, Count: 3	File: SPEECH, Count: 4
File: BADO, Count: 3	File: SOFT, Count: 4	File: SQUEEZE, Count: 3
File: CHALLENGE, Count: 2	File: ARRANGE, Count: 7	File: TOUR, Count: 4
File: MIDDLE, Count: 2	File: LICK, Count: 3	File: VACATE, Count: 2
File: POWERFUL, Count: 3	File: TAX, Count: 4	File: CURSE, Count: 2
File: FEW, Count: 3	File: DELAY, Count: 4	File: NAKED, Count: 2
File: LOCK, Count: 2	File: MANAGEMENT, Count: 4	File: VALUABLE, Count: 3
File: BEE HIVE, Count: 2	File: PRINT, Count: 3	File: CIGARETTE, Count: 4
File: CHASE, Count: 2	File: SECRETARY, Count: 4	File: COUGH, Count: 5
File: HONEY, Count: 7	File: CARPENTER, Count: 4	File: FINGER, Count: 2
File: INTERRUPT, Count: 4	File: FARMER, Count: 7	File: MILK, Count: 12
File: M-E-L-L-Y, Count: 2	File: SLAVERY, Count: 4	File: SLIM, Count: 2
File: POLITICIAN, Count: 9	File: LIBRARY, Count: 3	File: THIN, Count: 5
File: TITLE, Count: 2	File: THERMOMETRE, Count: 2	File: BURIAL, Count: 5
File: CHOKE, Count: 2	File: ADULTS, Count: 2	File: FUND, Count: 4
File: FILE, Count: 4	File: ADVISOR, Count: 3	File: IMPROVE, Count: 5
File: HOLIDAY, Count: 6	File: APPROVE, Count: 4	File: POLITICS, Count: 5

File: HUSBAND, Count: 3	File: RULES, Count: 2	File: PROPHET, Count: 3
File: MINISTRY, Count: 2	File: PRACTISE, Count: 2	File: ADD, Count: 4
File: BOY BOY, Count: 4	File: STORM, Count: 4	File: OVER, Count: 4
File: DRIVING, Count: 2	File: AEROPLANE, Count: 2	File: COUNT, Count: 6
File: KITALE, Count: 2	File: BECAME, Count: 2	File: STRAW, Count: 3
File: LICENCE, Count: 4	File: BROWN, Count: 4	File: UNDERLINE, Count: 6
File: GOAL, Count: 4	File: MANDAZI, Count: 5	File: WITHDRAW, Count: 2
File: TALK, Count: 8	File: BELL, Count: 5	File: CONFIDENCE, Count: 4
File: TRACTOR, Count: 2	File: CLARIFY, Count: 2	File: KAPSABET, Count: 2
File: CALM, Count: 3	File: CONCLUDE, Count: 3	File: PROFESSOR, Count: 3
File: MAD, Count: 2	File: DICTATE, Count: 3	File: TORN, Count: 3
File: NEPHEW, Count: 2	File: OPPRESS, Count: 3	File: UNCLE, Count: 7
File: TOUT, Count: 3	File: AIM, Count: 2	File: WEBUYE, Count: 2
File: OFFER, Count: 2	File: PLAYER, Count: 2	File: BASE, Count: 2
File: WORD WORD, Count: 4	File: BISCUIT, Count: 2	File: LEMON, Count: 4
File: DARK, Count: 5	File: COCOA, Count: 2	File: SENT, Count: 7
File: HANDBAG, Count: 2	File: FINSH, Count: 2	File: BUILDER, Count: 2
File: OFFICER, Count: 2	File: CHRISTIAN , Count: 2	File: PAINTER, Count: 2
File: T-SHIRT, Count: 2	File: CHRISTMAS, Count: 4	File: EMPLOY, Count: 9
File: PEPPER, Count: 3	File: TEMPT, Count: 2	File: HALF, Count: 3
File: LADY LADY, Count: 2	File: EMPLOYEE, Count: 3	File: HOUSEHELP, Count: 2
File: VISITOR VISITOR, Count: 2	File: SIXTY, Count: 3	File: LOWER, Count: 2
File: INTRESTING, Count: 3	File: ELECT, Count: 2	File: PORRIDGE, Count: 4
File: LISTEN, Count: 3	File: INDEPENDENCE, Count: 6	File: SIX, Count: 2
File: LAKE, Count: 2	File: PARLIAMENT, Count: 2	File: WOMEN, Count: 3
File: CHOCOLATE, Count: 2	File: VARIETY, Count: 2	File: ESCORT, Count: 4
File: EVENT, Count: 2	File: BULLET, Count: 3	File: HANDKERCHIEF, Count: 4
File: MAGAZINE, Count: 4	File: BOOKS, Count: 5	File: LECTURER, Count: 3

File: CONTRIBUTION, Count: 4	File: JANE, Count: 2	File: LOSE, Count: 3
File: CUPBOARD, Count: 2	File: SUBTRACT, Count: 2	File: PUMP, Count: 3
File: FORCUS, Count: 2	File: ENEMY, Count: 2	File: RETURN, Count: 2
File: TIRESOME, Count: 2	File: FAIR, Count: 2	File: SWEATER, Count: 3
File: MUCH, Count: 10	File: FASHION, Count: 3	File: WONDERFUL, Count: 2
File: MUDDY, Count: 3	File: OURS, Count: 5	File: DISCOVER, Count: 2
File: SELFISH, Count: 3	File: PREFECT, Count: 2	File: EMERGE, Count: 2
File: CENTER, Count: 2	File: RUDE, Count: 4	File: REVEAL, Count: 2
File: CHILD CHILD, Count: 2	File: BECAUSE, Count: 3	File: SCRATCH, Count: 2
File: PAINFUL, Count: 3	File: SEAT, Count: 2	File: TEST, Count: 3
File: YOURSELF, Count: 4	File: SIT, Count: 4	File: CABINET, Count: 4
File: H-I-T, Count: 2	File: CLUB, Count: 2	File: CEILING, Count: 2
File: STUDENT STUDENT, Count: 7	File: PINEAPPLE, Count: 2	File: STATEMENT, Count: 3
File: EXPLAINATION, Count: 2	File: PUPPET, Count: 2	File: SUSPECT, Count: 4
File: SHY, Count: 2	File: RECOMMEND, Count: 2	File: THEIRS, Count: 7
File: ARM, Count: 2	File: WHOLE, Count: 2	File: THEN, Count: 2
File: PROMOTE, Count: 2	File: EXAMPLE, Count: 2	File: COMMON, Count: 2
File: PUBLIC, Count: 4	File: FOR, Count: 2	File: COPY, Count: 3
	File: RETIRE, Count: 9	File: ESCAPE, Count: 3
		File: FORCE, Count: 6

UNIQUE VIDEO FILES/WORDS		
**ROW 1**	**ROW 2**	**ROW 3**
File: ROUP	File: THINKING	File: COUNSLE
File: VLLAGE	File: TIMBER	File: KISS
File: EXCERCISE	File: TOM0RROW	File: OBSERVE
File: TEACHING	File: TRADE	File: SABBATH
File: FOUND	File: TRAIN(TEACH)	File: SIR
File: INTERUPT	File: UNDERNEATH	File: VOLUNTARY
File: SHRUBS	File: WATCHMAN	File: DOUBLE
File: CALENDAR	File: WAVE (SEA)	File: SHAPE CURVE
File: CELEBRATIONS	File: EARBUD	File: SLIGHTLY
File: DISCONNECTED	File: ERECT	File: ANTI-CLOCKWISE
File: CRISTMASS	File: FGM	File: N-E-U-T-R-A-L
File: HOLY SPIRIT	File: GRAPE	File: SIBLING (BROTHER,SISTER)
File: PREACHING	File: BERRET	File: BUSHY
File: COMFORTAABLE	File: FELIX	File: EGG EGG
File: ARRESTED	File: LAB	File: KREST
File: SIDE YOUR	File: MAURINE	File: SATISFIED
File: 2010	File: TSHIRT	File: E-R-A-S-E
File: HONESTY	File: LEGUMES	File: S-T-R-I-K-E
File: ISSUE	File: AGE	File: SLOWLY SLOWLY
File: KENYANS	File: ANCESTORS	File: EXCUSE EXCUSE
File: INGREDIENT	File: JOSHUA	File: GEORGIOUS
File: ADULT	File: MEMORISE	File: HI
File: HUNT	File: MIRIAM	File: MINERAL MINERAL
File: PROMISED	File: STAN	File: SERIAL

File: CAMEL	File: VISTOR	File: B-O-L-T
File: LAUGH	File: LAY	File: C-A-S-E
File: CAPACITY	File: SHOWN	File: COLOURBLIND
File: DISEASES	File: TOM	File: DINE
File: RAPTURE	File: ALSO	File: HARASS
File: SOME ONE	File: BYE	File: KING KING
File: VIRUS	File: EARDRUM	File: MAN MAN
File: WAITINGROOM	File: JEFF	File: S-T-I-F-F
File: 9 AM	File: REMARKS	File: STEERING
File: CHILD CHILD	File: TERRY	File: SIMILAR
File: DOCTRO	File: DIED	File: W-E-A-L-T-H
File: EACH OTHER	File: GROOM	File: BANK
File: INSTITUTION	File: JOVIAL	File: MEMBERS
File: SWORKER	File: WINNIE	File: TAP
File: OPPONENTS	File: TANA	File: 0
File: PROBLEM	File: FLOWERS	File: 10
File: PESIDENT	File: FUL	File: 24
File: SHOULD	File: NEIVEROL	File: 4
File: EASIER	File: ROT	File: BY
File: GERMANY	File: BULB	File: CRATE
File: JUNE	File: GREETING	File: DIVIDE
File: SEPTEMBER	File: LIKELY	File: RECORD
File: E-X-P-E-N-S-I-V-E	File: NEUTRAL	File: SOLD
File: INDIANS	File: OPPORTUNE	File: CANE
File: M-I-A-M-I	File: COMBINE	File: FERTILE
File: P-E-D-A-G-O-G-Y	File: NYANGOMA	File: AHEAD
File: TUITION	File: SPARE	File: SPACE
File: BIOLOGY	File: VOCABULARY	File: SUPER

File: BOOST	File: FRUITS	File: VERTICAL
File: CHEMISTRY	File: ONIONS	File: MINDS
File: CONTIMUE	File: ROLLS	File: OPPOSIT
File: GEOGRAPHICAL	File: SATISFY	File: SURFACE
File: HISTORY	File: SWAMP	File: VALUE
File: NATURAL	File: TOMATOE	File: ANTONIO
File: PHYSICS	File: ARREAS	File: ATIENO
File: RELIGIOUS	File: DISSAPEAR	File: BRIDEGROOM
File: RELIGIUOS	File: EXECUSE	File: HERIDETARY
File: TERMS	File: QUESTIONMARK	File: MALE
File: ARTEFACTS	File: SPECIAL	File: MY
File: MUSCIAN	File: BOLT	File: NIECE
File: OPEN	File: CASECASS	File: SWEETHEART
File: C-L-E-V-E-R	File: WILL	File: WRINKLES
File: CHOSE	File: DRILL	File: IMAGINE
File: J-O-S-E-P-H	File: FLUSH	File: LUCK
File: OTHER OTHER	File: LIGHTS	File: MARRIED
File: TILL	File: PROP	File: SURPRISE
File: ABIT	File: QUICK	File: CHAMPION
File: DIFFERENCE	File: SORT	File: EXTREMELY
File: K-I-M-A-N-I	File: STURBORN	File: PATIENCE
File: HOLD ON	File: GUN	File: UPSET
File: KIMANI	File: PUSH	File: ERASE
File: LEFT OVER	File: R-A-I-D	File: ANOTHER
File: ARABS	File: BOTLE	File: NETBALL
File: BANTU	File: CRAYONS	File: BENEATH
File: GIRIAMA	File: DIARY	File: DIDE
File: INDIA	File: INSTRUCTIONS	File: SOUTH

File: JEWS	File: JUG	File: SUDAN
File: MERU	File: L-I--N-D-A	File: COCKROACH
File: SAUDI ARABIA	File: MAIN	File: EYES
File: TRIBE	File: MINOR	File: NOSTRIL
File: ATHLETICS	File: OPERATION	File: BRAIN
File: COMPOSITION	File: PASSAGE	File: COMPILE
File: PAGE	File: B-A-N	File: SURE
File: CLAUSE	File: B-R-I-D-G-E	File: NOTES
File: EXCERCISE BOOKS	File: BARK	File: SACRAMENT
File: ITEMS	File: CROTCHET	File: SERIOIUS
File: STANZA	File: D-I-S-P-L-A-Y	File: SERMON
File: DELEGATE	File: DIG	File: CHAPLAIN
File: BABIES	File: H-I-N-G-E	File: DEPUTY
File: FOEST	File: JEMBE	File: MUSHROOM
File: TDAY	File: L-I-T-T-L-E	File: FAILURE
File: EXCITED	File: MICROPHONE	File: INDISCPLINE
File: CHICKEN POX	File: N-A-I-L	File: LACK
File: PENCILS	File: NEEDLE	File: MISUSED
File: CELLPHONE	File: P-O-L-I-S-H	File: PERFOM
File: HARBOR	File: P-R-I-C-K	File: PERFOMANCE
File: OACEN	File: PROJECTOR	File: PRAISE
File: PUNCTURE	File: R-U-B-B-I-S-H	File: SENSE
File: YESRTERDAY	File: RAKE	File: CASHEWNUTS
File: ESTIMATE	File: S-T-E-E-L	File: COOL
File: NOTYET	File: SCREW	File: ROAST
File: POLE	File: W-H-O-L-E N-I-G-H-T	File: CHOIR
File: AGAINT	File: YELLOW	File: DIAGRAM
File: APPROACH	File: B-U-T-U-L-A	File: FLIGHT

File: BEND	File: BONGOMA	File: JOHN
File: CAREFULLY	File: C-H-O-N-Y	File: OTIENO
File: ABSOLUTE	File: C-O-M-P-R-O-M-I-S-E	File: WARNING
File: ANGLE	File: C-O-N-O-L-E-T	File: NICE TWIST
File: DEFENCE	File: D-I-C-H-O-T-O-M-O-U-S	File: CITIZENS
File: IGNORANCE	File: ECLIPSE	File: CONGREGATION
File: ILLITERATE	File: S-T-Y-L-E	File: EUROPEENS
File: INTELLIGENCE	File: S-U-B-V-E-R-T	File: IMPOSE
File: OPPORTUNITIES	File: STUFF	File: SPRINT
File: SKEPTICAL	File: C-H-A-R-L-E-S	File: DIRECTORS
File: VARY	File: CROWD	File: SKILLED
File: ACCOMPLISHMENT	File: A-R-T-I-C-L-E	File: ACCRUE
File: PROPER	File: EFFECTIVE	File: SOLDER
File: WONDER	File: GARDEN	File: TASKS
File: FOUL	File: I-N-T-E-R-J-E-C-T-I-O-N	File: WORKERS
File: LLOW	File: PRONOUN	File: CHRISTIANS
File: MADMAN	File: SPRINKLE	File: TASTES
File: MENSTRUATION	File: SYRINGE	File: NITHING
File: MOOD	File: VERB	File: STRIKING
File: MORTUARY	File: WEED	File: EVIRONMENT
File: ORGAN	File: WHISTLE	File: COUNSELLOR
File: REMARKABLE	File: WINNOWING	File: KEEP
File: WOUMB	File: CABLE	File: 2010
File: 18	File: FACEBOOK	File: ISSUES
File: 64	File: FEATURE	File: LEADERS
File: A-V-O-C-A-D-O	File: PHYSICAL	File: NATIONAL
File: ADDICT	File: PIG	File: YEARS
File: ADDITION	File: T-E-X-T	File: LANGUAGE

File: ARRIVED	File: T-R-E-N-D	File: WEEDING
File: ATTACK	File: CELLOTAPE	File: VOLINTER
File: B-E-T	File: CONTAINER	File: GOOD
File: BATHROOM	File: EVERYWHERE	File: 2PM
File: BATTLE	File: HOLDER	File: BIAS
File: BELL RING	File: S-L-A-B	File: EXERCISE
File: BELLOW	File: STAND	File: OLYMPIC
File: BLOW	File: THERMOS FLASK	File: SCORE
File: C-O-M-P-L-E-X	File: DIZZY	File: SKIP
File: C-O-M-P-L-I-C-A-T-E-D	File: RELIEVE	File: DINING HALL
File: C-O-N-V-E-N-T	File: STINGY	File: FETCH
File: C-R-U-S-E	File: BROKEN	File: MENU
File: C0RNER	File: C-A-V-E	File: PORK
File: CAPTURE	File: YERSTERDAY	File: WARM
File: CARVE	File: CULT	File: DEVIL
File: CATHOLIC	File: EXCECUTIVE	File: DISCIPLES
File: CHAMELEON	File: STRANGLE	File: PASTORS
File: CLAN	File: FLUSH DISK	File: WRITTER
File: COMPETITIVE	File: I-N-H-E-R-I-T	File: 1964
File: CONFLICT	File: KINGSHIP	File: LAWS
File: CONTACT	File: LATRINE	File: MAYOR
File: COOPERATION	File: P-R-I-E-S-T	File: BARREN
File: CREATION	File: PULL OUT	File: KILLL
File: CREATIVE	File: RAPE	File: MIRROW
File: DISOBEY	File: LITER	File: MEDIA
File: E-R-O-D-E	File: SEEDLING	File: RESPONSIBILITIES
File: E-X-I-S-T	File: THRRE	File: POUR
File: EXPORTS	File: A-R-R-I-V-E	File: ADORE

File: F-R-O-N-T	File: A-T-T-E-N-D	File: FORESEE
File: FANTASY	File: BEUTIFUL	File: OATH
File: FAST (SPEED)	File: CATS	File: ORDAIN
File: FOX	File: CELEBRATION	File: SAD
File: GOAL KEEPER	File: DOGS	File: LORY
File: GOSSIP	File: GRADUTION	File: RINGING
File: H-A-L-L	File: INFORMATION	File: WISEMAN
File: H-U-R-T	File: LION	File: DISPLAY
File: HOW MUCH	File: OURSELVES	File: DRAG
File: IDOL IDOL	File: PHOTOGRAGH	File: GUMMY
File: IMMIGRATION	File: VISITRO	File: PRODUSE
File: IMPORT	File: A-P-O-L-O-G-I-Z-E	File: SUITCASE
File: INSECT	File: ELDERS	File: CHECK
File: INTERNATIONAL	File: ENUOGH	File: CRIPPLE
File: JUNIOUR	File: KNOCK	File: DESCRIBED
File: K-E-R-U-G-O-Y-A	File: P-A-R-D-O-N	File: DISABILITY
File: L-U-C-Y	File: POLITELY	File: HEARING
File: LOW	File: THANK	File: OFTENLY
File: M-O-S-Q-U-E	File: THANKFUL	File: OPINION
File: MANSION	File: WAVE	File: PHILOSOPHY
File: MOMBASA	File: ACCESS	File: PITCH
File: MULTIPLICATION	File: DOWNLOAD	File: PRIORITY
File: NEGLECT	File: M-O-D-E-R-N	File: RAMP
File: ORPHAN	File: SMARTPHONE	File: WHEEL CHAIR
File: OVAL	File: STUDIES	File: MOTHER
File: OWL	File: A-I-D-S	File: SECOND (POSITION)
File: P-A-R-A-D-I-S-E	File: GAMES	File: C0NTROL
File: P-E-R-F-O-R-M	File: H-I-V	File: HONOR

File: P-R-O-G-R-E-S-S	File: INFECT	File: RESPONSBILITY
File: P-R-O-V-E-R-B	File: PREVENTION	File: STRENGTHEN
File: PACKED	File: SALIVA	File: TAX TAX
File: PARTICIPATE	File: SILIVA	File: THAR
File: PETROLIUM	File: TOTHBRUSH	File: BROKE
File: PLAYER PLAYER	File: W--E-A-L-T-H	File: RESIGNATION
File: POUCHING	File: ABOVE	File: ACCUATION
File: PREFECTS	File: COST	File: CORNFIRM
File: PRETEND	File: NOTHUNG	File: GOVERMENT
File: REASON	File: REACH	File: NATIONAL ATHEM
File: REPENT	File: SHADE	File: ADMINSTRATION
File: RESOLUTION	File: CONSUMER	File: CONFIRMED
File: RESURRECTION	File: EMLOYER	File: INSTITUTE
File: RING (WEDDING)	File: PROSTITUTES	File: PRINCIESS
File: S-H-A-R-O-N	File: UNION	File: REGISTERED
File: S-H-I-R-T	File: B-E-G-I-N	File: FATHER(GOD)
File: S-O-L-D-I-E-R	File: D-I-V-E	File: STORIES
File: S-P-L-I-T	File: F-A-R-M	File: MODIFY
File: SAILAS	File: G-R-A-B	File: CONVENE
File: SAVED	File: HARDWORK	File: DELGATE
File: SAVIOUR	File: I- N-V-A-D-E	File: KAJIADO
File: SHELTER	File: K-E-N	File: KERUGOYA
File: SHORT (HEIGHT)	File: N-O-T-E	File: KISII
File: SPECTATOR	File: REQUIRES	File: MUMIAS
File: SPORT SPORT	File: SUFFERING	File: NAKURU
File: STIR	File: SYSTEM	File: VOI
File: STRIDE	File: T-E-M-P-O-R-A-R-Y	File: SOLDIER SOLDIER
File: SWORD	File: T-O-Y	File: BUTERE

File: T-I-E	File: LAUNCH	File: EMBU
File: T-U-R-N	File: P-R-A-C-T-I-C-A-L-S	File: KABARNET
File: TAXED	File: P-R-A-C-T-I-C-A-L	File: KILIFI
File: THE	File: S-K-I-R-T	File: KITUI
File: THEORY	File: S-K-Y	File: LIMURU
File: SMOKER	File: VEGETABLE	File: NAIVASHA
File: CONSCIOUS	File: D-O-N-O-RS	File: NYANDARUA
	File: 6	File: NYANZA
File: WORM	File: CATWALK	File: PROVINCE PROVINCE
	File: CONFIDENT	File: TRAIN (EDUCATION)
File: TRAFFIC	File: CONSTRUTION	File: CHARACTOR
	File: CRIMINAL GANG	File: GREEN GRAMS
File: SHORT SLEEVE	File: MKE	File: DOCTOR DOCTOR
File: BRACELETS	File: POTHOLE	File: VISITOR VISTOR
File: COSMETIC	File: CANDIDATES	File: BENEFIT
File: NEAT NEAT	File: FANS	File: FOCUSS
File: POLISH	File: INTO	File: AT
File: TONGUE	File: SWARM	File: HAND HOLD
File: BEAUTFUL	File: DAMP	File: SHE SHE
File: PROFITABLE	File: EXGGERATE	File: BELONG
File: SHOES	File: MODE	File: COMING
File: CREAT	File: NECKLASE	File: DIVORCE
File: STRANGE	File: A-L-L-E-R-G-Y	File: KINGDOM
File: SMOOTH	File: BONE	File: PREACHER
File: FACTS	File: DISCOLOUR	File: SINS
File: HOPEFUL	File: FIGURE	File: SOLOMON
File: PREPARED	File: GAIN	File: UNTILL
File: RELEAS	File: I-A-N	File: JUNIOR PRIMARY

File: 8	File: ILLEGAL	File: C-R-A-Y-O-N
File: RIGHT(DIRECTION)	File: LOSS	File: M-A-T-C-H
File: WORKHOME	File: PREVENT	File: C-E-M-E-T-R-Y
File: EXAGGERATE	File: SNEEEZE	File: CLAY
File: HANDOVER	File: THIN	File: COTTON
File: SELLS	File: WEIGHT	File: DAM
File: ROBBER	File: ANOINT	File: ERASER
File: INDIPENDENCE	File: DEPEND	File: GOALKEEPER
File: SHRUB	File: MISSIONARY	File: MADE
File: TEACHER TEACHER	File: PRETENT	File: TORCH
File: FRIEND FRIEND	File: SINGING	File: GUITER
File: SHOT	File: WELCOME	File: HANDLE
File: COOKER	File: ACTVITY	File: PINEAPPLES
File: DOMITORY	File: IMPLEMENT	File: SCISSORS
File: 1	File: LEARN	File: BEYOND
File: CHINISE	File: MATERIAL	File: MET
File: FULL STOP	File: POLOCY	File: RASHES
File: TRAINING	File: RESERCH	File: MUNITE
File: CHANGAA	File: SEPARATE	File: HORIZON
File: SANDWICH	File: A-M-E-R-I-C-A	File: LINES
File: LAGGAGE	File: BRACKET	File: HIND
File: STRIP	File: DISCIPLINE	File: SOLDIERS
File: TOTOISE	File: MANDATORY	File: NORTH
File: APPETITE	File: O-T-H-E-R-S	File: OR
File: DETERIORATE	File: SCHOLARSHIP	File: SOUTH AFRICA
File: PRIVATE	File: SHAPES	File: TWINS
File: KIDNEY KIDNEY	File: SPELL	File: CURVE
File: MUMP	File: C-O-M-P-L-E-T-I-O-N	File: MISTAKES

File: STOMACHACHE	File: E-N--V-E-L-O-P-E	File: ERODE
File: UNCONCIOUS	File: E-N-V-E-L-O-P-E	File: GOSSIPING
File: NARROW NARROW	File: FAIT	File: TREMBLE
File: DRESS DRESS	File: MINUTES	File: F-E-T-C-H
File: MOTORBIKE	File: NEWS	File: HOURS
File: SHORT SLEEVED	File: PRESENTATION	File: MOTHER-IN-LAW
File: BRACELET	File: S-E-A-L	File: RELATIVES
File: COSMET	File: MOTHER TONGUE	File: SEED
File: CREATE	File: PRE-SCHOOL	File: I-N-T-E-R-R-U-P-T
File: STRANGER	File: WIND	File: L-A-P-T-O-P
File: CLOSS	File: HAT	File: PARENTS
File: FRIUT	File: INDUSTRIES	File: T-A-B-L-E-T-S
File: ATTENTION	File: KANGAROO	File: TOO FAT
File: FACT	File: M-O-L-O	File: BAPTISM
File: INTENTION	File: P-R-O-F-E-S-S-O-R	File: GRADUATION
File: OPTIMISTIC	File: SHOEPOLISH	File: OURSELF
File: WAKE	File: CONDUCT	File: SURPRISED
File: AWATD	File: DOMINION	File: BEAD
File: SCOUT SCOUT	File: FATHER INLAW	File: CALF
File: HORK	File: INFRASTRUCTURE	File: DUCK
File: HANDING	File: L-O-G-I-S-T-I-C	File: LOAD
File: IMPEDE	File: MUNICIPALITY	File: MODERN
File: EASYS	File: SOLDRIES	File: OBEDIENT
File: PEDAGOGY	File: THINGS	File: RECENT
File: TIGHT	File: TROUPE	File: T-O-N-N-Y
File: TUTION	File: D-O-M-I-C-I-L-E	File: J-A-N-E
File: BACKWARDS	File: DRIVEWAY	File: SINCE
File: DISAPPEARED	File: ELDRES	File: W-A-R-D

File: BELCH	File: FURNITURE	File: B-I-L-L
File: DINING	File: HOUSE KEEPER	File: MAGSTRATE
File: FAMOUS	File: INTERIOR	File: RUBBISH
File: FIGHTERS	File: JOBLESS	File: STORE
File: HEROES	File: NURSING	File: INCORRECT
File: HEROINE	File: PEOPLE	File: UNIT
File: ITSELF	File: PET	File: CERTIFICATE
File: RESPECTED	File: S-U-M-M-O-N	File: GRADUATION
File: TRAP	File: CHILD	File: EXPERIMENT
File: WAITER	File: CHILD LABOUR	File: NOMBER
File: CAMPING	File: E-S-T-E-E-M	File: RELIRION
File: EENTERTAIN	File: G-U-M-B-O-O-T	File: TREACHER
File: FISHING	File: M-E-R-C-Y	File: HARD WORK
File: FOREVER	File: O'CLOCK	File: ATHLETIC
File: JOG	File: STEP	File: SCHEDULE
File: LEISURE	File: T-E-X-T-I-L-E	File: BISHOPS
File: BEGGAR	File: UTENSILS	File: DISASTER
File: COURAGEOUS	File: HIS	File: FORERSEE
File: HAPPINESS	File: HURRY	File: HEART
File: HAPPINNESS	File: KIDNAP	File: JEALOUS
File: HARMONY	File: LEAN	File: MERCY
File: MATTER	File: LESSON	File: NEIGHBOR
File: QUARRELLING	File: REJECT	File: PROPHECY
File: PLOUGHING	File: REVISE	File: PROPHESY
File: SHOPPING	File: RUNNER	File: PURE
File: PERSONAL	File: BEAR	File: WILLING
File: COMB	File: SUBMERGE	File: CRIME
File: REPEAT	File: TALENT	File: DOCTRINE

File: RING(WEDDING RING)	File: WINNER	File: ENVY
File: ABUSE	File: C-U-R-I-O-U-S	File: EVE
File: DEMONSTRATE	File: HYPOTHESIS	File: FORBID
File: EXPAND	File: IT''S	File: GOSPEL
File: D0CTOR	File: IT'S	File: POLYGAMY
File: FIRST AID	File: MENTAL DISORDER	File: TEMPTATION
File: HURT	File: PHYLOSOPHY	File: TESTAMENT
File: ILLINESS	File: SILLY	File: HURTS
File: INJURY	File: MUM	File: JESUS CHRIST
File: OBJECT	File: ORGANIZE	File: POEM
File: PLACE PLACE	File: R-E-S-O-R-T	File: ROMAN
File: PLACE PLACE	File: SO	File: S-I-L-A
File: POISONOUS	File: U-N-I-Q-U-E	File: SAVIOR
File: STING	File: ENGAGE	File: CANDIDATE
File: COCKROACHES	File: EXPELL	File: KNIVE
File: CRACK	File: PURSUADE	File: TOOTH BRUSH
File: P-A-L-E	File: ROOT ROOT	File: CLOUD CLOUD
File: ALTER	File: STRIVE	File: PE0POLE
File: ANNOINTING	File: INSTINCT	File: VEGETATION
File: DEACON	File: BEDROOM	File: ASSOCIATIONS
File: ROBE(STEAL	File: EVIDENCE	File: DEPT
File: ROYAL	File: EXPENDITURE	File: SCHHOL
File: WISH (REGRET)	File: SEX	File: C-A-T-W-A-L-K
File: LOGISTIC	File: BOX BOX	File: CONFIDENTLY
File: NEGATIVE	File: CEILLING BOARD	File: CRIMINAL
File: C-O-R-P-S-E	File: BIRD BIRD	File: NOISE
File: CHAIRPERSON	File: DROVE	File: P-O-T-H-O-L-E
File: CUSTOMER CUSTOMER	File: F-I-N-G-E-R-S-P-E-L-L	File: P-R-E-M-I-S-E

File: D-I-S-T-R-A-C-T	File: F-R-I-D-G-E	File: R-U-N
File: PEOPLE A LOT	File: FACULTY	File: S-T-R-U-T-T
File: POPE	File: FRY FRY	File: V-A-C-A-T-E
File: SHEET	File: L-A-S-T	File: KWALE
File: NOTICE	File: L-E-C-T-U-R-E-R	File: SOLDIER SOLDIER
File: PROVE	File: PETAL	File: TAITA
File: RESCUE	File: PLANT PLANT	File: TANA RIVER
File: SWEATER	File: R-E-V-E-R-S-E	File: TRAVELLING
File: WEAVE	File: R-O-M-E	File: VEHECLE
File: GIRL GIRL	File: BRITISH	File: CONCRETE
File: LOOSE	File: C-O-N-T-A-I-N	File: FANTA
File: 3	File: COLONIZE	File: FESTIVE
File: COOK (PERSON)	File: DECOMPOSE	File: SPICES
File: EXPORT	File: P-O-S-T-M-O-T-E-R-M	File: STEEL
File: GRANDPARENT	File: P-R-E-A-C-H-E-R	File: EQUALLY
File: TUSKER	File: P-R-O-C-E-D-U-R-E	File: I-T-E-M
File: YOGHUT	File: P-R-O-C-U-R-E	File: J-A-R
File: 2	File: POST(POLE)	File: ROUGH
File: LEARNER	File: PROCREATE	File: ASKED
File: ROCKET	File: SPIN	File: BEGIN
File: BATTERY	File: SUCCESSFUL	File: D-E-L-V-E-R
File: BONNET	File: THIING THING	File: FAN
File: HIRED	File: CANCER	File: K-E-N
File: LANE	File: CHEEK	File: MONTHLY
File: MECHANIC	File: CHEST	File: NOTE
File: STREET	File: JUNGLE	File: S-A-M
File: TRAILOR	File: K-A-M-B-E-D-T-H-I	File: S-P-E-E-C-H
File: BANDIT	File: KAKAMEGA	File: SWIMMER

File: EARN	File: WI-FI	File: TOY
File: EEVERYBODY	File: B-A-M-B-U-R-I	File: PUBERTY
File: PEANUT(GROUNDNUT)	File: CALENDER	File: CHIKENPOX
File: PILLOW CASE	File: H-E-E-L	File: SWELL
File: SLEEVE	File: MAJOR	File: SIGNBOARD
File: SLIPPERS	File: NOTEBOOK	File: STRETCH
File: SUCK	File: DEBTOR	File: ANNOUNCEMENTS
File: BEDSHEET	File: OPPORYUNITY	File: FARMERS
File: GUIDANCE	File: R-O-Y-A-L	File: GRAND
File: PAVEMENT	File: SAMOSA	File: RUB
File: BEAN	File: T-Y-C-O-O-N	File: MUSTACHE
File: BLEND	File: THANK YOU	File: RESPONSIBLE
File: PILAU	File: WHOLE NIGHT	File: DRUMS
File: SALTY	File: AWARE	File: DOCUMENT
File: THOUGHTFUL	File: CIRCUMCISION	File: DONORS
File: UMBILICAL CORD		File: HEADQUARTERS
File: BOTTLE BOTTLE	File: SECRETAY	File: BICYCLEMAN
File: RUGBY	File: INERESTING	File: CONSTRUCTER
File: TROPHY	File: LIAR	File: PROFESSER
File: TUG	File: SHRUB SHRUB	File: KEEPER
File: CHAIR CHAIR	File: SWEATR	File: COMMITEE
File: GUITAR	File: INDIAN	File: EXEMPLARY
File: LEAKING	File: DEVELOPED	File: ALLEGATION
File: COACH(GAMES)	File: MEMBER	File: CAMPAIGN
File: CROSS (MSALABA)	File: CCOKER	File: CONFIRM
File: CROSS(MSALABA)	File: NOTHIG	File: NATION
File: ENEMITY	File: SOLUTION	File: TOPICS
File: K-E-E-N	File: CHINEES	File: CONFIRM.FINISH

File: MELON	File: SCKNESS	File: ELECTIONS
File: NOWDAYS	File: UDERLINE	File: COMITTEE
File: RAILWAY	File: SHE	File: INTRESTED
File: TOUGH	File: STEAL	File: AIRPOT
File: CROCODILE	File: 20	File: COUNTRIES
File: D-I-S-M-A-L-L-Y	File: ENEMIES	File: TOMMOROW
File: E-L-A-B-O-R-A-T-E	File: HER	File: BUFFALLO
File: IDLE	File: DISAPPOINTMENT	File: DEER
File: CEMENT	File: HOPE	File: TESTIMONY
File: CHALKBOARD	File: SANDWHICH	File: BEATIFUL
File: FRAME	File: REMOVE	File: SIGN POST
File: FATHER-IN-LAW	File: CRUTCH	File: REGRET
File: HOUSE-HELP	File: AIDS	File: SPORT
File: L-A-W-Y-E-R	File: S-W-E-L-L	File: STORY STORY
File: STIRRE	File: VOMITING	File: IPORTANT
File: TELEVISION	File: DANGAROUS	File: THIRST
File: C-A-R-P-E-T	File: TAX	File: CYCLIST
File: EROD	File: RETIREMENT	File: HERDSMAN
File: FIREWOOD	File: TYCOON	File: DANCER
File: FOLD	File: ACCUSATION	File: STRENTHEN
File: HEADGIRL	File: PRESDENT	File: HOSPITAL
File: M-A-T-A-T-U	File: FIERC	File: SWAHILI
File: P-A-R-A-D-E	File: PLATE PLATE	File: BROUGHT
File: SLIDE	File: SUIT	File: JELOUSY
File: TRACE	File: T -SHIRT	File: W-I-D-E
File: UNFOLD	File: AVOCADO	File: WORLDWIDE
File: CIRCUMCISE	File: BEAN BEAN	File: BEATRICE
File: E-R-E-C-T	File: GROUNDNUTS	File: BRIDEGOOM

File: ENTICE<br>File: F-G-M<br>File: PEG PEG<br>File: BUTTON BUTTON<br>File: F-E-L-I-X<br>File: GLOVE GLOVE<br>File: LABCOAT<br>File: M-A-U-R-I-N-E<br>File: SCARF<br>File: J-O-S-H-U-A<br>File: M-I-R-R-I-A-M<br>File: TOURIST<br>File: UGANDA<br>File: P-O-S-T<br>File: SLOW SLOW<br>File: SPELLING	File: MANGO MANGO<br>File: MEN<br>File: MONKEY MONKEY<br>File: ONION ONION<br>File: ORANGE ORANGE<br>File: PROTEIN<br>File: A-L-L-A-N<br>File: ANCESTOR ANCESTOR<br>File: C-H-I-N-E-S-E<br>File: COMPANION<br>File: DEMOCRATIC<br>File: FEMALE<br>File: GENERATION<br>File: J-E-F-F<br>File: T-E-L-E-G-R-A-M<br>File: T-E-R-R-Y<br>File: VIBRATION	File: BROTHER-IN-LAW<br>File: FAITHFUL<br>File: J-O-V-I-A-L<br>File: WIDOW<br>File: FRESH<br>File: T-A-N-A<br>File: VICTORIA<br>File: HERS HER<br>File: MIGORI<br>File: COLORFUL<br>File: NUETRAL<br>File: R-O-T<br>File: BROOM<br>File: COLUMN<br>File: B-R-I-E-F<br>File: E-A-R-D-R-U-M

AI4KSL VIDEO PROCESSING PHASE II

Total Video Count- 7155

So far, the collected data in phase 2 has prepared batches 1 and 2 for processing. The description is as follows per batch.

BATCH 1 - ***Total Video Count: 3252***

Duplicate video files

File: A LOT, Count: 14<br>File: ALL, Count: 11	File: NOISE, Count: 2<br>File: PHOTO, Count: 2	File: PICK, Count: 3<br>File: SERMON, Count: 2

File: AT, Count: 2<br><br>File: BEFORE, Count: 2<br><br>File: BURNING, Count: 2<br><br>File: BUY, Count: 14<br><br>File: CALL, Count: 6<br><br>File: CARRY, Count: 6<br><br>File: CHILDREN, Count: 8<br><br>File: COMPANY, Count: 7<br><br>File: DEAD, Count: 2<br><br>File: DISPLAY, Count: 2<br><br>File: DRY, Count: 3<br><br>File: EARLY, Count: 4<br><br>File: ENJOY, Count: 4<br><br>File: EXCITE, Count: 2<br><br>File: FAMILY, Count: 8<br><br>File: FESTIVAL, Count: 5<br><br>File: FINISH, Count: 39<br><br>File: FIREWOOD, Count: 2<br><br>File: FIRM, Count: 2<br><br>File: FIRST, Count: 2<br><br>File: FISH, Count: 2<br><br>File: FIST, Count: 2<br><br>File: GIRL, Count: 10<br><br>File: GIVE, Count: 11<br><br>File: HAVE, Count: 41<br><br>File: HE, Count: 18<br><br>File: HIS, Count: 15<br><br>File: IMPORTANT, Count: 6	File: RECORD, Count: 2<br><br>File: SHE, Count: 30<br><br>File: TEACHER, Count: 9<br><br>File: THEY, Count: 12<br><br>File: WATER, Count: 9<br><br>File: BOOK, Count: 5<br><br>File: COUNTRY, Count: 7<br><br>File: DECORATE, Count: 3<br><br>File: DOG, Count: 2<br><br>File: FAR, Count: 2<br><br>File: GATHER, Count: 2<br><br>File: HOUSE, Count: 13<br><br>File: KNOW, Count: 9<br><br>File: LAKE, Count: 2<br><br>File: LEAVE, Count: 3<br><br>File: LIGHT, Count: 2<br><br>File: NEAR, Count: 2<br><br>File: NOTHING, Count: 28<br><br>File: REPORT, Count: 2<br><br>File: SIT, Count: 2<br><br>File: SLEEP, Count: 2<br><br>File: THERE, Count: 21<br><br>File: TRUE, Count: 5<br><br>File: .mp4, Count: 20<br><br>File: BRIGHT, Count: 4<br><br>File: DESIGN, Count: 2<br><br>File: DESK, Count: 2<br><br>File: DOCUMENT, Count: 2	File: SOME, Count: 9<br><br>File: THIS, Count: 27<br><br>File: WE, Count: 22<br><br>File: WHERE, Count: 11<br><br>File: WOOD, Count: 2<br><br>File: YOU, Count: 28<br><br>File: ZERO, Count: 16<br><br>File: A, Count: 3<br><br>File: AFRICA, Count: 2<br><br>File: EMPLOYEE, Count: 2<br><br>File: FATHER, Count: 11<br><br>File: GO, Count: 11<br><br>File: GOD, Count: 4<br><br>File: HERE, Count: 2<br><br>File: IN, Count: 6<br><br>File: MAIN, Count: 2<br><br>File: MAKER, Count: 3<br><br>File: P, Count: 2<br><br>File: PHONE, Count: 2<br><br>File: PLEASE, Count: 18<br><br>File: RECEIVE, Count: 3<br><br>File: STUDENT, Count: 6<br><br>File: THEIR, Count: 5<br><br>File: UNIFORM, Count: 3<br><br>File: WANT, Count: 15<br><br>File: WHO, Count: 11<br><br>File: WOMAN, Count: 3<br><br>File: WORSHIP, Count: 3

File: LEARN, Count: 4	File: FILE, Count: 2	File: YOUR, Count: 26
File: LINE, Count: 4	File: FLY, Count: 2	File: BEST, Count: 3
File: MAKE, Count: 8	File: FULL, Count: 2	File: BREAK, Count: 4
File: MORNING, Count: 2	File: GOVERNMENT, Count: 6	File: C, Count: 2
File: NEED, Count: 26	File: HIGH, Count: 3	File: COOK, Count: 2
File: PLACE, Count: 8	File: INTERNATIONAL, Count: 2	File: E, Count: 3
File: PROBLEM, Count: 3	File: LEG, Count: 2	File: KEEP, Count: 6
File: REQUIRE, Count: 3	File: MAN, Count: 14	File: LOVE, Count: 22
File: SAME, Count: 16	File: MANY, Count: 27	File: MANAGE, Count: 4
File: SEE, Count: 5	File: MATCH, Count: 3	File: PEOPLE, Count: 16
File: SERIOUS, Count: 3	File: NEW, Count: 17	File: S, Count: 2
File: SERVICE, Count: 2	File: ONE, Count: 10	File: SMELL, Count: 3
File: SHOW, Count: 3	File: PERSON, Count: 3	File: TASTE, Count: 2
File: SKY, Count: 2	File: SMALL, Count: 5	File: VISIT, Count: 4
File: THAT, Count: 44	File: SUPPORT, Count: 2	File: WHITE, Count: 5
File: TICKET, Count: 2	File: TEAM, Count: 2	File: BAD, Count: 3
File: TIME, Count: 21	File: WIN, Count: 5	File: FRIDAY, Count: 2
File: USE, Count: 20	File: ZOO, Count: 2	File: HEALTHY, Count: 3
File: WAY, Count: 4	File: BIG, Count: 10	File: JOIN, Count: 3
File: WORK, Count: 18	File: BRING, Count: 3	File: MANIPULATION, Count: 2
File: AIR, Count: 2	File: DOOR, Count: 6	File: MEETING, Count: 4
File: ALWAYS, Count: 9	File: EXPENSIVE, Count: 4	File: MONEY, Count: 4
File: ATTEND, Count: 2	File: GOOD, Count: 12	File: N, Count: 3
File: BEAUTIFUL, Count: 11	File: HARD, Count: 2	File: NEXT, Count: 6
File: BOY, Count: 8	File: HOME, Count: 11	File: PRESIDENT, Count: 4
File: BUSINESS, Count: 8	File: LABORATORY, Count: 2	File: SCHOOL, Count: 11
File: CAR, Count: 16	File: ME, Count: 50	File: SECRETARY, Count: 2
File: CHANGE, Count: 4	File: MINE, Count: 44	File: STEAL, Count: 2

File: COMMUNICATE, Count: 2	File: MOST, Count: 2	File: TELL, Count: 5
File: CRUCIAL, Count: 2	File: OUR, Count: 22	File: TREE, Count: 2
File: FIND, Count: 4	File: FUTURE, Count: 4	File: YEAR, Count: 8
File: FINGERPRINT, Count: 2	File: J-A-N-E, Count: 5	File: ANY, Count: 3
File: FRIEND, Count: 5	File: OTHER, Count: 5	File: CROP, Count: 3
File: HELP, Count: 12	File: PEN, Count: 2	File: DIFFERENT, Count: 2
File: IMPROVE, Count: 4	File: SIN, Count: 3	File: DRAW, Count: 4
File: INVESTIGATE, Count: 2	File: SOIL, Count: 3	File: FARMER, Count: 4
File: LOAN, Count: 2	File: STOP, Count: 5	File: GEOGRAPHY, Count: 2
File: NICE, Count: 6	File: US, Count: 14	File: MUST, Count: 16
File: COW, Count: 3	File: AIM, Count: 2	File: THOSE, Count: 3
File: DESTROY, Count: 4	File: DATE, Count: 2	File: COLOUR, Count: 4
File: DISEASE, Count: 2	File: JOB, Count: 6	File: DRESS, Count: 3
File: FALL, Count: 4	File: KILL, Count: 3	File: EASY, Count: 2
File: FAST, Count: 7	File: WALL, Count: 2	File: LADY, Count: 2
File: GOAT, Count: 2	File: YELLOW, Count: 2	File: LITTLE, Count: 3
File: IMPOSSIBLE, Count: 3	File: ACCIDENT, Count: 2	File: MARK, Count: 2
File: LOOK, Count: 3	File: COMPUTER, Count: 2	File: MARRIAGE, Count: 2
File: POSSIBLE, Count: 9	File: EDUCATION, Count: 3	File: MARRY, Count: 2
File: UNDER, Count: 2	File: PROJECT, Count: 4	File: MASENO, Count: 2
File: ARM, Count: 3	File: SPEECH, Count: 2	File: OLD, Count: 7
File: AROUND, Count: 3	File: WEALTH, Count: 2	File: PAST, Count: 14
File: BECOME, Count: 5	File: WISDOM, Count: 2	File: POINT, Count: 2
File: BENEFIT, Count: 3	File: ACT, Count: 3	File: RED, Count: 2
File: BETTER, Count: 5	File: BIRD, Count: 3	File: WEAR, Count: 4
File: BLUE, Count: 2	File: DAVID, Count: 2	File: WEDDING, Count: 3
File: CURL, Count: 2	File: FROM, Count: 4	File: CULTURE, Count: 2
File: EUROPEAN, Count: 2	File: GREAT, Count: 2	File: DOCTOR, Count: 5

File: FACE, Count: 5	File: GROW, Count: 4	File: FEAR, Count: 6
File: FOREIGN, Count: 2	File: INVITE, Count: 2	File: J, Count: 2
File: HAIR, Count: 2	File: MEAN, Count: 3	File: KIND, Count: 3
File: LIVE, Count: 3	File: TOMORROW, Count: 9	File: LIKE, Count: 8
File: MACHINE, Count: 2	File: TRAVEL, Count: 6	File: MATATU, Count: 2
File: SOON, Count: 5	File: TREAT, Count: 4	File: MOTHER, Count: 8
File: STRONG, Count: 5	File: WITH, Count: 3	File: TWO, Count: 5
File: WEAK, Count: 2	File: ASK, Count: 2	File: VERY, Count: 11
File: AFFECT, Count: 3	File: FEEL, Count: 4	File: WILL, Count: 10
File: CROSS, Count: 3	File: KISS, Count: 2	File: YESTERDAY, Count: 7
File: PARENT, Count: 4	File: WAR, Count: 3	File: CONTINUE, Count: 2
File: POVERTY, Count: 2	File: ALLOW, Count: 2	File: FOOTBALL, Count: 2
File: RIVER, Count: 5	File: BROTHER, Count: 3	File: HOW, Count: 7
File: ROAD, Count: 2	File: CAT, Count: 2	File: ON, Count: 3
File: SELL, Count: 4	File: CLASS, Count: 2	File: PAY, Count: 4
File: SHOE, Count: 2	File: FIGHT, Count: 3	File: PLAY, Count: 2
File: THESE, Count: 2	File: K-I-M, Count: 2	File: THREE, Count: 3
File: CUSTOMER, Count: 2	File: KITTEN, Count: 2	File: ALOT, Count: 3
File: DEEP, Count: 2	File: SNAIL, Count: 2	File: CAN, Count: 10
File: HUSBAND, Count: 2	File: TOUCH, Count: 2	File: CRY, Count: 3
File: MARKET, Count: 2	File: UNITY, Count: 2	File: DIRTY, Count: 6
File: RIGHT, Count: 2	File: UNIVERSITY, Count: 2	File: DO, Count: 4
File: SKILL, Count: 4	File: WEEK, Count: 5	File: HEAR, Count: 2
File: SOLVE, Count: 2	File: WEEKEND, Count: 2	File: HER, Count: 12
File: WELL, Count: 6	File: AFTER, Count: 2	File: LOUD, Count: 2
File: AMERICA, Count: 2	File: BELOW, Count: 2	File: NOW, Count: 12
File: APPEAR, Count: 2	File: EAT, Count: 4	File: PLANT, Count: 3
File: BABY, Count: 8	File: HOLE, Count: 3	File: TORN, Count: 2

File: EXPERIENCE, Count: 2	File: NOSE, Count: 2	File: WOMEN, Count: 2
File: HEALTH, Count: 2	File: PUNCH, Count: 2	File: COLD, Count: 3
File: MILK, Count: 2	File: STONE, Count: 2	File: COME, Count: 9
File: NIGHT, Count: 2	File: KENYA, Count: 3	File: DAY, Count: 5
File: WALK, Count: 2	File: KUJA, Count: 2	File: FOOD, Count: 7
File: CLEVER, Count: 2	File: PASS, Count: 3	File: NAME, Count: 2
File: DANGER, Count: 2	File: SISTER, Count: 2	File: SICK, Count: 4
File: DARK, Count: 2	File: SPECIAL, Count: 3	File: TODAY, Count: 10
File: DOWN, Count: 3	File: SUBJECT, Count: 2	File: AGAIN, Count: 2
File: EYE, Count: 2	File: TAKE, Count: 3	File: ANIMAL, Count: 3
File: HAS, Count: 3	File: LABOUR, Count: 2	File: DIE, Count: 4
File: HATE, Count: 4	File: LONG, Count: 2	File: IF, Count: 2
File: ROOM, Count: 2	File: TOWN, Count: 3	File: JESUS, Count: 2
File: BLOOD, Count: 2	File: CLIMB, Count: 2	File: KING, Count: 3
File: CHALLENGE, Count: 2	File: ENOUGH, Count: 2	File: ME , Count: 6
File: DRUG, Count: 4	File: FARM, Count: 3	File: MONTH, Count: 3
File: EMPLOY, Count: 2	File: FLOWER, Count: 2	File: STORY, Count: 2
File: ENCOURAGE, Count: 3	File: LAMP, Count: 2	File: TEACH, Count: 10
File: HOSPITAL, Count: 2	File: LEAN, Count: 2	File: BANANA, Count: 2
File: CHILD, Count: 3	File: MEET, Count: 4	File: CLEAN, Count: 4
File: CHURCH, Count: 3	File: SEAT, Count: 3	File: CUP, Count: 3
File: DOWNLOAD, Count: 2	File: SHOES, Count: 2	File: DANGEROUS, Count: 4
File: PICTURE, Count: 2	File: TALL, Count: 2	File: DEAF, Count: 2
File: POOR, Count: 3	File: DRIVE, Count: 2	File: GET, Count: 3
File: REPAIR, Count: 5	File: LARVA, Count: 2	File: GROUP, Count: 3
File: ROPE, Count: 2	File: MOVE, Count: 2	File: HOT, Count: 2
File: SHORT, Count: 2	File: OFF, Count: 2	File: LAST, Count: 6
File: WATCH, Count: 2	File: ATTACK, Count: 2	File: MORE, Count: 6

File: FOLLOW, Count: 2	File: BUILD, Count: 4	File: POUR, Count: 2
File: PAINT, Count: 3	File: CORRUPT, Count: 2	File: PRACTICE, Count: 5
File: FAT, Count: 2	File: LAW, Count: 3	File: WHAT, Count: 6
File: MATURE, Count: 3	File: LAWYER, Count: 2	File: WHY, Count: 12
File: TOPIC, Count: 2	File: POLICE, Count: 2	File: PRIVATE, Count: 2
File: ADVANCE, Count: 2	File: ACCUSE, Count: 2	File: DISCUSS, Count: 2
File: ADVANTAGE, Count: 2	File: CORRECT, Count: 2	File: HIM, Count: 3
File: ADVICE, Count: 3	File: FALSE, Count: 2	File: MISTAKE, Count: 2
File: EXPLAIN, Count: 2	File: ACTION, Count: 2	File: RELATION, Count: 2
File: FREE, Count: 2	File: DREAM, Count: 2	File: BADO, Count: 2
File: SALARY, Count: 2	File: GOAL, Count: 2	File: WHEN, Count: 2
File: AFFAIR, Count: 2	File: MOVIE, Count: 2	File: NUMBER, Count: 2
File: FINANCIAL, Count: 3	File: DAMAGE, Count: 2	File: BODY, Count: 2
File: HUMAN, Count: 2	File: OFFICE, Count: 3	File: FAVOUR, Count: 2
File: ADDRESS, Count: 2	File: SON, Count: 3	File: TOGETHER, Count: 2
File: ADMINSTRATOR, Count: 2	File: SYSTEM, Count: 3	File: DELAY, Count: 2
File: DECISION, Count: 2	File: ADDICT, Count: 2	File: FEATURE, Count: 2
File: FINAL, Count: 2	File: AWARD, Count: 2	File: MEDAL, Count: 2
	File: MEDICAL, Count: 2	File: CREATE, Count: 2
	File: MATURITY, Count: 2	File: OBEY, Count: 2

UNIQUE VIDEO FILES

File: AID	File: CUBICLE	File: POLITICIAN
File: AMBULANCE	File: CUCUMBER	File: POWER
File: BOAT	File: IDENTIFY	File: SEX
File: CELEBRATE	File: ILLNESS	File: SUGAR
File: CHOP	File: PAINFUL	File: TRADITION
File: EMERGENCY	File: PROCEDURE	File: B-I-G-G-I-E
File: ESTABLISH	File: SALAD	File: CHOOSE
File: FIREWORK	File: SCAN	File: COCONUT
File: FIREWORKS	File: SHIRT	File: ENERGY
File: FOCUS	File: UNDERGO	File: FEW
File: FUN	File: D-A-V-I-D	File: KILIFI

File: GIRL THAT	File: DAUGHTER	File: KINDNESS
File: LEGAL	File: DAWN	File: KINETIC
File: LIGHT UP	File: IN LAW	File: NO
File: NURSE	File: OPEN	File: OIL
File: PRESENT	File: TUESDAY	File: RESPONSIBLE
File: READY	File: WAKE	File: RUNNING
File: SPECIALIZE	File: WISE	File: SPILL
File: VOICE	File: APPLE	File: SUCCESSFUL
File: FIANCEE	File: CAGE	File: THANK
File: HOLIDAY	File: CRUCIFICATION	File: THANK
File: PROPOSE	File: CRUCIFICIFICATION	File: THEORY
File: ALPHABET	File: CRUCIFY	File: THOUSAND
File: ANNALYSIS	File: CRUEL	File: WIND
File: BUDGET	File: CRUNCH	File: WIND
File: CLEAR	File: CRUNCHED	File: BRAVE
File: COLLEGE	File: CRUTCHED	File: COMPOSE
File: DEMONSTRATE	File: CRUTCHES	File: FOREVER
File: DEPARTMENT	File: DEATH	File: GOLIATH
File: DETECTIVE	File: DENY	File: ISRAEL
File: DIRECTION	File: ENDURE	File: ISRAELITE
File: FILM	File: INNOCENT	File: J-O-H-O-N
File: FILTER	File: LATE	File: KINGDOM
File: FINANCE	File: MESSENGER	File: KINGSHIP
File: FINGER SPELL	File: MOTHER INLAW	File: KISII
File: FINGER SPELLING	File: SNOW	File: PLENTY
File: FINGERPRNT	File: THIEF	File: R-A-I-L-A
File: FINGERSPELL	File: UNTIL	File: REIGN
File: GLASS	File: USE	File: SECOND
File: HANDLE	File: CULT	File: SERVIVOR
File: IMPRESS	File: CULTIVATE	File: SLAUGHTER
File: IMPURITY	File: CULTIVATION	File: SONG
File: INDEPEDENT	File: DIVERSE	File: VICTOR
File: INTERPRETER	File: HEARING	File: BEAUFUL
File: MANAGEMENT	File: MAIZE	File: E-D-I
File: PERSONAL	File: OWN	File: K-I-T-H
File: PRACTISE	File: PROUD	File: KITALE
File: PREFER	File: SEASON	File: KITUI
File: QUALITY	File: SHIFTING	File: LARGE
File: REDUCE	File: TEA	File: NANE
File: REMOVE	File: ARRANGE	File: O-T-I
File: SEMINAR	File: BITTER	File: PROVINCE
File: STUDY	File: CHEW	File: WEDNESDAY
File: TAP	File: CUD	File: WESTERN
File: VISION	File: CUPBOARD	File: WITHCRAFT
File: ARMCHAIR	File: CURE	File: ALTER
File: BLANKET	File: DISH	File: COURSE
File: CAPITAL	File: HOW MANY	File: FARMING
File: CHARGE	File: SPIT	File: GRANDFATHER

File: FAMOUS<br>File: FINLAND<br>File: FIRERSIDE<br>File: FIRESIDE<br>File: GLOW<br>File: HEAT<br>File: HELSINKI<br>File: RELAX<br>File: STACK<br>File: SUSPECT<br>File: ANKLE<br>File: ATTRACT<br>File: BITE<br>File: BUILDING<br>File: CEREMONY<br>File: COLOR<br>File: CONFERENE<br>File: EDGE<br>File: END<br>File: FEET<br>File: FLAG<br>File: FLAMINGO<br>File: FLASH DISK<br>File: FLEA<br>File: FLOCK<br>File: GRACE<br>File: HABITAT<br>File: ITCH<br>File: LAPTOP<br>File: NEAT<br>File: OBOVE<br>File: ORGANIZE<br>File: PERFORM<br>File: PINK<br>File: PORTABLE<br>File: RAISE<br>File: SAVE<br>File: SLAM<br>File: STAND<br>File: TRANSFER<br>File: WAVE<br>File: COURT<br>File: FOREST<br>File: HEAVY<br>File: LUO<br>File: MAGIC<br>File: MAGICIAN<br>File: MAGISTRATE	File: SPOON<br>File: BRITISH<br>File: CURIOUS<br>File: CURRENCY<br>File: CURRICULUM<br>File: FRENCH<br>File: INTRODUCE<br>File: MINISTRY<br>File: PLAN<br>File: SCHOOL<br>File: SMOKE<br>File: SNAKE<br>File: STABLE<br>File: UPSTAIRS<br>File: CURSE<br>File: CURTAIN<br>File: CURVE<br>File: CUSHITE<br>File: CUSHITES<br>File: CUSTODY<br>File: EAST<br>File: INVOLVE<br>File: MOUNTAIN<br>File: PEACE<br>File: BOY<br>File: CARE<br>File: COMPETITION<br>File: CULTLERY<br>File: CUT<br>File: CYCLIST<br>File: EXCELLENT<br>File: FOREHEAD<br>File: GIFT<br>File: HIT<br>File: ISSUE<br>File: KNIFE<br>File: PART<br>File: PERSONNEL<br>File: PINEAPPLE<br>File: SERVE<br>File: STATION<br>File: YOURSELF<br>File: CLOTHE<br>File: CLOTHES<br>File: CYCLONE<br>File: DAGORETTI<br>File: DAGORRETI<br>File: DAM	File: HEAD<br>File: J-O-S-E<br>File: KNEE<br>File: KNEEL<br>File: KNIT<br>File: KNOCK<br>File: PAIN<br>File: R-O-S-E<br>File: RIBS<br>File: ROCK<br>File: SKIRT<br>File: SNAILS<br>File: THING<br>File: THROW<br>File: WHTE<br>File: DEVOTE<br>File: DRINK<br>File: EXAM<br>File: EXTENSIVE<br>File: IMPORT<br>File: K-C-S-E<br>File: K-S-L<br>File: KNOWLEDGE<br>File: KOREA<br>File: KREST<br>File: LEARNER<br>File: PASSAGE<br>File: SURPRISE<br>File: WITHOUT<br>File: BIRTH<br>File: COAT<br>File: D-A-N-I-E-L<br>File: EVENING<br>File: GOVERNOR<br>File: INTERVIEW<br>File: J-A-M-E-S<br>File: KURIA<br>File: KWALE<br>File: LABEL<br>File: LABORATOFY<br>File: M-R<br>File: P-A-U-L<br>File: PHOTOGRAPH<br>File: W-A-N-J-A-L-A<br>File: AFRICAN<br>File: AGAINST<br>File: BED<br>File: CELEBRATION

File: MAGNET<br>File: MAHOGANY<br>File: MAID<br>File: OBJECT<br>File: BEHAVIOR<br>File: DISCIPLINE<br>File: MAID<br>File: MAIL<br>File: MAINTAIN<br>File: MAJOR<br>File: MALAWI<br>File: WORD<br>File: CHEF<br>File: FRIDGE<br>File: I<br>File: KISUMU<br>File: MALE<br>File: MALINDI<br>File: MALL<br>File: MALTOSE<br>File: MAMMARY GLAND<br>File: MUSLIM<br>File: ORGAN<br>File: PROTESTANT<br>File: CABINET<br>File: CHAIRMAN<br>File: COMMON<br>File: FORM ONE<br>File: FRUIT<br>File: M<br>File: MANDATE<br>File: MANGO<br>File: MANIPULATION<br>File: MESSAGE<br>File: NOMINATE<br>File: ORDER<br>File: PARENTS<br>File: PRINCIPAL<br>File: REPLY<br>File: RESCHEDULE<br>File: STRATEGY<br>File: BILLIONAIRE<br>File: MANSION<br>File: MANURE<br>File: MAP<br>File: WORLD<br>File: CHEAT<br>File: COUNT	File: DUMP<br>File: OVER FLOW<br>File: PROPERTY<br>File: RAIN<br>File: SLUM<br>File: STRANGE<br>File: SWEEP AWAY<br>File: CAUSE<br>File: DANCE<br>File: DANCER<br>File: ELECTRICITY<br>File: FIRE<br>File: HIDE<br>File: M-A-R-Y<br>File: MALARIA<br>File: MOSQUITO<br>File: POSTER<br>File: S-A-M-B-A<br>File: SAME<br>File: UP<br>File: ABENTSIM<br>File: ABILITY<br>File: ABORT<br>File: ABORT<br>File: ABORTION<br>File: ABSENT<br>File: ABSENTISM<br>File: ABSORB<br>File: ADMIT<br>File: INK<br>File: J-O-H-N<br>File: NOTE<br>File: ROOT<br>File: STREAN<br>File: AGONY<br>File: AGREE<br>File: AGRICULTURE<br>File: AIRCRAFT<br>File: AIRCRAFT<br>File: AIRFORCE<br>File: BUILT<br>File: CRASH<br>File: LONG AGO<br>File: LONG TIME AGO<br>File: TO<br>File: ABSTAIN<br>File: ABSTRACT<br>File: ABUSE	File: COVER<br>File: FLOWERED<br>File: FORCE<br>File: GOWN<br>File: GRADUATE<br>File: GREEN<br>File: L-A-K-I<br>File: LABOURER<br>File: LACE<br>File: LADDER<br>File: PREPARE<br>File: SEW<br>File: TIGHT<br>File: A LITTLE<br>File: CAMP<br>File: CHICK<br>File: INDIAN<br>File: INSIDE<br>File: LAGGUAGE<br>File: LAMU<br>File: LANDLORD<br>File: LANE<br>File: LAP<br>File: LIQUID<br>File: LOST<br>File: OUTSIDE<br>File: OVER<br>File: PARK<br>File: PUT<br>File: STICK<br>File: STRICT<br>File: TABLE<br>File: 2024<br>File: ACTIVITY<br>File: AUNCH<br>File: COLLAPSE<br>File: DESCISION<br>File: ENEMY<br>File: LATRINE<br>File: LAUGH<br>File: LAUNCH<br>File: LUAGH<br>File: MINISTER<br>File: NUTRITIOUS<br>File: OFFICIAL<br>File: PAPER<br>File: RETIRE<br>File: SUN

File: GERMANY	File: ACCELERATE	File: ACCOMPANY
File: MARINE	File: ACCENTUATE	File: ACCOUNTANT
File: MAROON	File: ACCESS	File: ACCUSATION
File: MISS	File: ADVISE	File: MURDER
File: OFFICER	File: AVOID	File: WRONG
File: SPOT	File: BEAUTY	File: ACHIEVE
File: TERRITORY	File: CLOTH	File: ACQIURE
File: THEME	File: DISCUSSION	File: ACQUIRE
File: TRICKY	File: DRIVER	File: ACQUIRE
File: CHEAP	File: IDEA	File: ACROSS
File: FARE	File: MANY MANY	File: AGROVERT
File: H	File: MEAL	File: BALL
File: MAASAI	File: MEDICINE	File: DELETE
File: MASK	File: OK	File: FUNNY
File: MASON	File: POLICY	File: LANGUAGE
File: MASTER	File: QUICK	File: OTHER
File: O	File: ADDICTION	File: PARTY
File: AGEMATE	File: ADJECTIVE	File: SHOP
File: COTTON	File: ADJOURN	File: BLOCK
File: MATE	File: ADMINISTRATION	File: BOX
File: MATERIAL	File: ADMINSTRATION	File: CANCER
File: MATERNITY	File: CHAANGE	File: CELL
File: MATHARE	File: DISCRIBE	File: CORRUPTION
File: SUCCESS	File: EXAMPLE	File: DEVELOP
File: C-T	File: MEETTING	File: DUCT
File: CALF	File: BE	File: GLAND
File: GROUND	File: BELONG	File: GLOBAL
File: LEADERER	File: DOMINION	File: GLOBE
File: LESS	File: DONKEY	File: GLOBLE
File: PILLOW	File: DONOR	File: GLOVE
File: SOFT	File: DORMITORY	File: GLUE
File: A-P-P	File: GRASS	File: GOALKEEPER
File: ABOUT	File: MAT	File: HEAL
File: FEDERATION	File: THOUGHT	File: PATIENT
File: FRIENDLY	File: DAD	File: PROSTRATE
File: HIGHLIGHT	File: DIVISION	File: RUN
File: IDEAL	File: DIVORCE	File: SALIVA
File: MEMBER	File: DIZZY	File: SENTENCE
File: ORGANISE	File: DOCTRINE	File: WHICH
File: PROMOTE	File: DOLL	File: 4
File: SAFETY	File: HEIGHT	File: ACTOR
File: TRIBE	File: NEVER	File: ADAM
File: ATHLETE	File: NOON	File: ADAPT
File: BAN	File: PASTOR	File: ADDICT
File: BOND	File: RESEARCH	File: ARREST
File: BONE	File: SALVATION	File: CONTENT
File: CONNECT	File: ANGEL	File: ADOLECENT
File: DIAGNOS	File: BEDROOM	File: ADOLESCE

File: ELEPHANT<br>File: EQUAL<br>File: EVERYDAY<br>File: EXCEL<br>File: FELLOWSHIP<br>File: FEMALE GENITAL MUTILATION<br>File: FEMALE<br>File: FEMUR<br>File: FORM<br>File: FRACTURE<br>File: GENITAL MUTILATION<br>File: GOLD<br>File: HAPPY<br>File: HIP<br>File: K-N-E-E<br>File: MILLION<br>File: OPPORTUNITY<br>File: PHYSICAL<br>File: PROVIDE<br>File: RECOVER<br>File: SEVERE<br>File: THERAPY<br>File: WELCOME<br>File: X-RAY<br>File: APPLY<br>File: BACKYARD<br>File: BOARD<br>File: BROKEN<br>File: CHEMICAL<br>File: ENVIRONMENT<br>File: EVERY<br>File: FENCE<br>File: FERRY<br>File: FERTERLISER<br>File: FERTERLIZER<br>File: FERTILE<br>File: FERTILISER<br>File: FOG<br>File: GARDEN<br>File: HAPPEN<br>File: HOUR<br>File: IMAGINATION<br>File: ISLAND<br>File: LAND<br>File: MUSIC<br>File: P-A-R-K<br>File: PASSANGER	File: BLACK<br>File: BOTH<br>File: BURN<br>File: CALM<br>File: DOT<br>File: DOVE<br>File: GRANDMOTHER<br>File: IMAGE<br>File: MIND<br>File: SCREEN<br>File: ADD<br>File: ALL DAY<br>File: BEHAVIOUR<br>File: COACH<br>File: DITCH<br>File: DOZE<br>File: DRAINAGE<br>File: DRAMA<br>File: DRAWER<br>File: ECONOMY<br>File: FLOW<br>File: INTERNATE<br>File: PIPE<br>File: PULL<br>File: AWAY<br>File: BALL<br>File: BIRDS<br>File: CATTLE<br>File: CONDITION<br>File: DRILL<br>File: DRIZZLE<br>File: DROP<br>File: KICK<br>File: MUDDY<br>File: SHEEP<br>File: TIRED<br>File: DROUGHT<br>File: DRUM<br>File: DRUMMER<br>File: DUCK<br>File: DULL<br>File: EGG<br>File: UNCLE<br>File: ADMINSTRATOR<br>File: RESPECT<br>File: RETURN<br>File: AWARE<br>File: COLLECT	File: ADOLESCENCE<br>File: ADOLESCENT<br>File: ADOPT<br>File: ADORE<br>File: ADULT<br>File: DESIRE<br>File: LORD<br>File: PERIOD<br>File: STAGE<br>File: WAIT<br>File: ADVERTISE<br>File: ADVERTISEMENT<br>File: LIKE<br>File: LISTEN<br>File: ONLINE<br>File: PRODUCT<br>File: TECHNOLOGY<br>File: WEBSITE<br>File: ADVISOR<br>File: ADVOCATE<br>File: AERIAL<br>File: AEROPLANE<br>File: BOSS<br>File: EXPERT<br>File: FIX<br>File: INTEREST<br>File: INTERESTING<br>File: POLITICAL<br>File: RADIO<br>File: AFFILIATE<br>File: AFFILIATION<br>File: AFFORD<br>File: AGE<br>File: AGENDA<br>File: AGENT<br>File: ANGRER<br>File: BRAIN<br>File: IMMPOSSIBLE<br>File: LOOSE<br>File: WEATHER<br>File: CHOICE<br>File: DECIDE<br>File: DECLARE<br>File: DICIDE<br>File: NEIGHBOUR<br>File: AMOUNT<br>File: DCERATION<br>File: DECORATION

File: PRIVACY<br>File: PRODUCE<br>File: SEPARATE<br>File: SUMMER<br>File: CALCULATOR<br>File: CHAIR<br>File: COMFORTABLE<br>File: COMPULSARY<br>File: HARSH<br>File: MATHS<br>File: MATRESS<br>File: MATRON<br>File: PREVIOUS<br>File: SACK<br>File: FIGHTER<br>File: FREEDOM<br>File: LIMIT<br>File: MATIRITY<br>File: MAU MAU<br>File: MAXIMUM<br>File: MAYBE<br>File: MAYOR<br>File: PROCEED<br>File: REACH<br>File: SWEET<br>File: TRIP<br>File: VACCINATION<br>File: VIEW<br>File: CHILD CHILD<br>File: DINNER<br>File: FISHING<br>File: FORGIVE<br>File: GRANT	File: CRITICISE<br>File: DISPOSAL<br>File: F-A-X<br>File: FAIR<br>File: FAVOURITE<br>File: FEACES<br>File: HOBBY<br>File: LIVESTOCK<br>File: P-I-Z-Z-A<br>File: PROCESS<br>File: PROMOTION<br>File: RECIEVE<br>File: REORT<br>File: RESTAURANT<br>File: SEND<br>File: V-E-T<br>File: ADMIRE<br>File: APPROACH<br>File: ATTITUDE<br>File: COLOURFUL<br>File: FEATHER<br>File: T-O-M<br>File: TRAGEDY<br>File: BANK<br>File: E-L-I-U-D<br>File: INSUARANCE<br>File: MEANINGFUL<br>File: MEANINGFUL<br>File: MEASLES<br>File: MEASURE<br>File: MECHANIC<br>File: MELON<br>File: METAL<br>File: OFFER	File: DECREASE<br>File: DEDUCT<br>File: HALL<br>File: OCEAN<br>File: PERCENTAGE<br>File: POT<br>File: ACCEPT<br>File: DEER<br>File: DEFEAT<br>File: DEFEND<br>File: DEFICIENCY<br>File: DEGRADE<br>File: RICH<br>File: V-I-T-A-M-I-N<br>File: FASHION<br>File: REWARD<br>File: START<br>File: TREND<br>File: BELT<br>File: COFFEE<br>File: CONSEQUENCE<br>File: FAITH<br>File: FASTEN<br>File: FATAL<br>File: J-U-N-K<br>File: LOW<br>File: RELIGION<br>File: STRUGGLE<br>File: SURVIVE<br>File: BLESS<br>File: LEAD<br>File: MANAGER<br>File: HEART<br>File: KNOWN

BATCH 2 -*Total Video Count: 3903*

Duplicate Video Files

File: .mp4, Count: 7<br>File: ANIMAL, Count: 4<br>File: BEST, Count: 8<br>File: BODY, Count: 6<br>File: COLD, Count: 7<br>File: FINISH, Count: 57<br>File: FUTURE, Count: 9<br>File: GET, Count: 4	File: BEAUTIFUL, Count: 8<br>File: BUILD, Count: 8<br>File: CLEAN, Count: 4<br>File: CLOSE, Count: 3<br>File: LIVE, Count: 5<br>File: MATERIAL, Count: 2<br>File: MORE, Count: 5<br>File: OLD, Count: 4	File: STEAL, Count: 4<br>File: FAR, Count: 3<br>File: FATHER , Count: 2<br>File: GERMANY, Count: 2<br>File: HAMMER, Count: 2<br>File: MOTHER, Count: 10<br>File: VEHICLE, Count: 2<br>File: CLOTHE, Count: 2

File: GIRL, Count: 22<br>File: GO, Count: 23<br>File: GOOD, Count: 32<br>File: HAVE, Count: 65<br>File: HIGH, Count: 6<br>File: HOME, Count: 15<br>File: KENYA, Count: 11<br>File: LIKE, Count: 7<br>File: MAN, Count: 18<br>File: MANY, Count: 32<br>File: ME, Count: 78<br>File: MUST, Count: 20<br>File: OUR, Count: 25<br>File: PROMISE, Count: 2<br>File: REMAIN, Count: 2<br>File: SCHOOL, Count: 11<br>File: SEE, Count: 16<br>File: SONG, Count: 2<br>File: STUDENT, Count: 15<br>File: THAT, Count: 56<br>File: THEY, Count: 19<br>File: THIS, Count: 45<br>File: TWO, Count: 10<br>File: US, Count: 7<br>File: WANT, Count: 26<br>File: WATER, Count: 8<br>File: WE, Count: 31<br>File: YOU, Count: 53<br>File: ZERO, Count: 38<br>File: ANGLE, Count: 2<br>File: ANTICLOCKWISE, Count: 2<br>File: BIRD, Count: 2<br>File: CHANGE, Count: 7<br>File: CHILDREN, Count: 8<br>File: DISCUSS, Count: 5<br>File: DOCUMENT, Count: 2<br>File: DRAW, Count: 7<br>File: FEEL, Count: 3<br>File: FEW, Count: 2<br>File: HOW, Count: 15<br>File: HUMAN, Count: 3<br>File: INTEREST, Count: 2<br>File: KNOW, Count: 11<br>File: MADE, Count: 2<br>File: MAKE, Count: 15<br>File: MANGO, Count: 2<br>File: MOVE, Count: 3<br>File: NEED, Count: 17	File: SOON, Count: 2<br>File: VISIT, Count: 6<br>File: ARM, Count: 2<br>File: DRINK, Count: 2<br>File: HIDE, Count: 2<br>File: LONG, Count: 5<br>File: SHOW, Count: 8<br>File: STORY, Count: 3<br>File: STRONG, Count: 2<br>File: ARTIFICIAL, Count: 2<br>File: BRING, Count: 4<br>File: BUY, Count: 16<br>File: COOK, Count: 8<br>File: IMPOSSIBLE, Count: 7<br>File: READ, Count: 2<br>File: SUBJECT, Count: 3<br>File: TOMORROW, Count: 6<br>File: WHERE, Count: 4<br>File: WRITE, Count: 4<br>File: BITTER, Count: 2<br>File: DOCTOR, Count: 6<br>File: HOLY, Count: 4<br>File: LEARN, Count: 5<br>File: WAIT, Count: 3<br>File: ACCIDENT, Count: 4<br>File: CAUSE, Count: 4<br>File: DESTROY, Count: 3<br>File: DIFFICULT, Count: 3<br>File: EXCERCISE, Count: 2<br>File: LIFE, Count: 4<br>File: MACHINE, Count: 2<br>File: POLICE, Count: 4<br>File: CHURCH, Count: 7<br>File: ENJOY, Count: 4<br>File: HAPPEN, Count: 2<br>File: MEET, Count: 5<br>File: MOVIE, Count: 2<br>File: OTHER, Count: 4<br>File: REAL, Count: 2<br>File: SELL, Count: 4<br>File: SISTER, Count: 9<br>File: SMALL, Count: 3<br>File: WATCH, Count: 2<br>File: YEAR, Count: 3<br>File: COLLECT, Count: 3<br>File: COMMON, Count: 3<br>File: COMMUNITY, Count: 2<br>File: CULTURE, Count: 2	File: HANG, Count: 2<br>File: HASSLE, Count: 2<br>File: PAY, Count: 3<br>File: PEACE, Count: 2<br>File: DANGEROUS, Count: 5<br>File: HAWK, Count: 2<br>File: KILL, Count: 6<br>File: SPIRIT, Count: 2<br>File: BREAK, Count: 3<br>File: CHICKEN, Count: 3<br>File: HEAD, Count: 2<br>File: HEAL, Count: 3<br>File: SICK, Count: 2<br>File: HEARTBEAT, Count: 2<br>File: CURE, Count: 2<br>File: PAINFUL, Count: 2<br>File: DAUGHTER, Count: 2<br>File: HIGHJUMP, Count: 2<br>File: NAIROBI, Count: 2<br>File: BUILDER, Count: 2<br>File: MEAN, Count: 8<br>File: SPREAD, Count: 2<br>File: PRESIDENT, Count: 2<br>File: LETTER, Count: 2<br>File: PERSON, Count: 2<br>File: DRESS, Count: 3<br>File: FAIR, Count: 2<br>File: PORRIDGE, Count: 2<br>File: SOLUTION, Count: 2<br>File: STOCK, Count: 2<br>File: TAILOR, Count: 2<br>File: VILLAGE, Count: 3<br>File: WOUND, Count: 2<br>File: HAS, Count: 7<br>File: LECTURER, Count: 3<br>File: NEIGHBOUR, Count: 2<br>File: PREVIOUS, Count: 2<br>File: STONE, Count: 3<br>File: WALL, Count: 3<br>File: CHILD, Count: 4<br>File: KISUMU, Count: 2<br>File: STAND, Count: 2<br>File: STAY, Count: 2<br>File: COMPETE, Count: 2<br>File: DIE, Count: 4<br>File: HARSH, Count: 2<br>File: S-T-R-O-L-L, Count: 2<br>File: ANGRY, Count: 2

File: NEW, Count: 14<br>File: OIL, Count: 4<br>File: PASS, Count: 4<br>File: PAST, Count: 11<br>File: PASTOR, Count: 2<br>File: PEOPLE, Count: 22<br>File: PLEASE, Count: 23<br>File: POSSIBLE, Count: 32<br>File: THOSE, Count: 8<br>File: THREE, Count: 5<br>File: TODAY, Count: 23<br>File: TOPIC, Count: 3<br>File: USE, Count: 21<br>File: WELL, Count: 7<br>File: WHEN, Count: 3<br>File: YOUR, Count: 32<br>File: FULL, Count: 5<br>File: MINE, Count: 59<br>File: MONTH, Count: 7<br>File: OCEAN, Count: 2<br>File: PLACE, Count: 11<br>File: REPORT, Count: 2<br>File: ROOM, Count: 2<br>File: TEACHER, Count: 11<br>File: TEN, Count: 7<br>File: VISITOR, Count: 2<br>File: ADMIRE, Count: 2<br>File: ALL, Count: 20<br>File: AMERICA, Count: 3<br>File: BRIGHT, Count: 3<br>File: BUILDING, Count: 3<br>File: CALL, Count: 5<br>File: CAR, Count: 8<br>File: DEAD, Count: 2<br>File: DIFFERENT, Count: 3<br>File: EXCITE, Count: 2<br>File: FACE, Count: 4<br>File: FEAR, Count: 2<br>File: FIND, Count: 2<br>File: FRIEND, Count: 6<br>File: GROUND, Count: 2<br>File: HELP, Count: 10<br>File: HIS, Count: 8<br>File: KEY, Count: 2<br>File: MAIN, Count: 3<br>File: OFTEN, Count: 3<br>File: OLYMPICS, Count: 2<br>File: ONE, Count: 5	File: EACH, Count: 3<br>File: FOLLOW, Count: 2<br>File: HARD, Count: 7<br>File: HUGE, Count: 3<br>File: IMPORTANT, Count: 6<br>File: MEETING, Count: 3<br>File: MEMBER, Count: 3<br>File: PARENT, Count: 2<br>File: PROVIDE, Count: 2<br>File: STRENGTH, Count: 2<br>File: WEEKEND, Count: 2<br>File: WHAT, Count: 13<br>File: ATHLETE, Count: 2<br>File: BEFORE, Count: 2<br>File: DRIVE, Count: 2<br>File: FAST, Count: 3<br>File: FUN, Count: 2<br>File: PRACTISE, Count: 2<br>File: RELIGION, Count: 2<br>File: REQUIRE, Count: 2<br>File: START, Count: 2<br>File: TRAIN, Count: 3<br>File: TYPE, Count: 2<br>File: ATTACK, Count: 2<br>File: BE, Count: 2<br>File: BECOME, Count: 9<br>File: BIG, Count: 11<br>File: DISEASE, Count: 4<br>File: FAT, Count: 2<br>File: HEART, Count: 4<br>File: SERIOUS, Count: 2<br>File: STRUGGLE, Count: 3<br>File: SURVIVE, Count: 2<br>File: THEM, Count: 12<br>File: TOGETHER, Count: 2<br>File: TREAT, Count: 3<br>File: WEDDING, Count: 2<br>File: WEEK, Count: 2<br>File: CLOTH, Count: 5<br>File: EDUCATION, Count: 3<br>File: EXPENSIVE, Count: 8<br>File: JESUS, Count: 4<br>File: NUMBER, Count: 2<br>File: PROJECT, Count: 2<br>File: TRAVEL, Count: 3<br>File: WHITE, Count: 4<br>File: WOMAN, Count: 3<br>File: WORD, Count: 4	File: BABY, Count: 4<br>File: BLACK, Count: 2<br>File: SUNNY, Count: 2<br>File: WEAR, Count: 4<br>File: CHEF, Count: 3<br>File: EXAM, Count: 2<br>File: HERE, Count: 2<br>File: CORRUPT, Count: 2<br>File: CRUSH, Count: 2<br>File: LIE, Count: 2<br>File: SHARE, Count: 2<br>File: TABLE, Count: 2<br>File: WHICH, Count: 2<br>File: SECRETE, Count: 2<br>File: COLLIDE, Count: 2<br>File: SHIRT, Count: 2<br>File: COMBINE, Count: 2<br>File: COMMEND, Count: 2<br>File: IN, Count: 2<br>File: COMMUNICATE, Count: 2<br>File: COMPILE, Count: 2<br>File: RESULT, Count: 2<br>File: COMPLAIN, Count: 2<br>File: COMPROMISE, Count: 2<br>File: CONFESS, Count: 2<br>File: SIN, Count: 2<br>File: CONFIRMATION, Count: 2<br>File: CONSTRUCTOR, Count: 2<br>File: BAG, Count: 2<br>File: CONTAINER, Count: 2<br>File: EARN, Count: 2<br>File: COMMUNICATION, Count: 2<br>File: MOTHER , Count: 2<br>File: WORSHIPPER, Count: 2<br>File: CONVINCE, Count: 2<br>File: COOPERATE, Count: 2<br>File: COORDINATE, Count: 2<br>File: COUNCIL, Count: 2<br>File: COVENANT, Count: 2<br>File: THINK, Count: 2<br>File: ANGER, Count: 2<br>File: DO, Count: 7<br>File: DRUG, Count: 3<br>File: EXAMPLE, Count: 2<br>File: EXCLUDE, Count: 2<br>File: HAPPY, Count: 2<br>File: LET, Count: 2

File: PHONE, Count: 3<br>File: PROBLEM, Count: 6<br>File: PUBLIC, Count: 2<br>File: SAME, Count: 18<br>File: SYSTEM, Count: 3<br>File: TAKE, Count: 7<br>File: THERE, Count: 30<br>File: TIME, Count: 31<br>File: WHY, Count: 15<br>File: BAD, Count: 7<br>File: FATHER, Count: 21<br>File: FISH, Count: 4<br>File: HOUSE, Count: 12<br>File: LOOK, Count: 2<br>File: MALE, Count: 2<br>File: PRINCIPAL, Count: 2<br>File: SELLER, Count: 2<br>File: SEND, Count: 2<br>File: SOME, Count: 12<br>File: SON, Count: 4<br>File: WORK, Count: 27<br>File: COMPANY, Count: 6<br>File: CONFIDENCE, Count: 2<br>File: COUNTRY, Count: 7<br>File: GIVE, Count: 12<br>File: JOB, Count: 5<br>File: MASTER, Count: 2<br>File: ROAD, Count: 4<br>File: SELF, Count: 2<br>File: SUCCESS, Count: 3<br>File: THEIR, Count: 5<br>File: A LOT, Count: 18<br>File: ADAM, Count: 3<br>File: AND, Count: 2<br>File: BUSINESS, Count: 2<br>File: CREATE, Count: 2<br>File: DAMAGE, Count: 2<br>File: EAT, Count: 11<br>File: GOD, Count: 10<br>File: INFORMATION, Count: 4<br>File: KEEP, Count: 5<br>File: NOTHING, Count: 17<br>File: NOW, Count: 8<br>File: PLAN, Count: 5<br>File: TELL, Count: 2<br>File: TEMPERATURE, Count: 2<br>File: TREE, Count: 3<br>File: TRUTH, Count: 3	File: WORLD, Count: 3<br>File: ATTEND, Count: 3<br>File: END, Count: 3<br>File: EVOLVE, Count: 2<br>File: FORBIDDEN, Count: 2<br>File: FRUIT, Count: 4<br>File: GOVERNMENT, Count: 3<br>File: HAIR, Count: 3<br>File: LAND, Count: 3<br>File: EYE, Count: 2<br>File: EYEBROW, Count: 2<br>File: LAW, Count: 2<br>File: LEFT, Count: 2<br>File: PAIN, Count: 2<br>File: PLANT, Count: 3<br>File: RIGHT, Count: 2<br>File: BLUE, Count: 2<br>File: COLOUR, Count: 3<br>File: COW, Count: 4<br>File: CRY, Count: 2<br>File: CUT, Count: 2<br>File: DIRTY, Count: 3<br>File: MORNING, Count: 2<br>File: RED, Count: 2<br>File: SHORT, Count: 2<br>File: ALOT, Count: 9<br>File: EASY, Count: 3<br>File: GRIND, Count: 2<br>File: GUEST, Count: 2<br>File: HONOUR, Count: 2<br>File: MAIZE, Count: 3<br>File: MEAT, Count: 4<br>File: NEXT, Count: 3<br>File: NICE, Count: 7<br>File: NURSE, Count: 3<br>File: SHE, Count: 24<br>File: TOMMOROW, Count: 10<br>File: WAY, Count: 3<br>File: CHEAP, Count: 6<br>File: DELICIOUS, Count: 2<br>File: GROUNDNUT, Count: 2<br>File: HEAR, Count: 4<br>File: MEDICINE, Count: 3<br>File: PRAY, Count: 2<br>File: SHOP, Count: 2<br>File: STORE, Count: 2<br>File: SWEET, Count: 4<br>File: GIFT, Count: 2	File: MANAGER, Count: 5<br>File: MYSELF, Count: 3<br>File: PREACH, Count: 2<br>File: RUTO, Count: 2<br>File: SHOE, Count: 3<br>File: SUGAR, Count: 2<br>File: WILD, Count: 2<br>File: BOOK, Count: 5<br>File: CHAIR, Count: 2<br>File: COMMITTEE, Count: 2<br>File: HE, Count: 24<br>File: REMOVE, Count: 3<br>File: BEE, Count: 2<br>File: SKILL, Count: 4<br>File: EXTEND, Count: 2<br>File: FENCE, Count: 2<br>File: HOLIDAY, Count: 2<br>File: TEA, Count: 3<br>File: TOWN, Count: 2<br>File: GROW, Count: 3<br>File: PARTY, Count: 3<br>File: SAY, Count: 2<br>File: SLOW, Count: 3<br>File: STRAIGHT, Count: 2<br>File: STREET, Count: 2<br>File: SUPPORT, Count: 2<br>File: TRUE, Count: 34<br>File: WALK, Count: 3<br>File: WIN, Count: 4<br>File: BEHAVIOUR, Count: 7<br>File: BUT, Count: 3<br>File: CAT, Count: 2<br>File: COME, Count: 9<br>File: HATE, Count: 10<br>File: LATE, Count: 4<br>File: LOVE, Count: 26<br>File: SLEEP, Count: 4<br>File: TEACH, Count: 6<br>File: ABOUT, Count: 5<br>File: ALWAYS, Count: 17<br>File: ARRIVE, Count: 3<br>File: BUS, Count: 3<br>File: CAN, Count: 14<br>File: COURT, Count: 3<br>File: ENOUGH, Count: 2<br>File: FLOW, Count: 2<br>File: FOOD, Count: 8<br>File: FOUR, Count: 3

File: WHO, Count: 7<br>File: WIFE, Count: 4<br>File: ALONG, Count: 2<br>File: AMAZE, Count: 2<br>File: BOY, Count: 11<br>File: CORRIDOR, Count: 2<br>File: EVE, Count: 3<br>File: IDEA, Count: 2<br>File: MONEY, Count: 2	File: GUIDE, Count: 2<br>File: GUILTY, Count: 2<br>File: HAUNT, Count: 2<br>File: THING, Count: 4<br>File: WILL, Count: 11<br>File: MY, Count: 5<br>File: NORMAL, Count: 3<br>File: YESTERDAY, Count: 9<br>File: OWN, Count: 2	File: BLOOD, Count: 2<br>File: BROTHER, Count: 6<br>File: GYMNASIUM, Count: 2<br>File: PLAY, Count: 2<br>File: SWALLOW, Count: 2<br>File: HER, Count: 8<br>File: HIM, Count: 11<br>File: LOST, Count: 2

Unique Video Files

File: AUTHORITY<br>File: CURRUPT<br>File: HORN<br>File: PARK<br>File: POEM<br>File: REHEARSE<br>File: REMIND<br>File: REVENGE<br>File: REVENUE<br>File: RHINO<br>File: THEY<br>File: AMMEND<br>File: AMMENDMENT<br>File: AMONG<br>File: ANATOMY<br>File: ANOINT<br>File: BILL<br>File: BISHOP<br>File: CLOCK<br>File: CONSTITUTION<br>File: DIVIDE<br>File: FROG<br>File: NERVOUS<br>File: PREACHER<br>File: ROTATE<br>File: SECTION<br>File: YOURSELVES<br>File: CYLINDER<br>File: FIELD<br>File: GEN-Z<br>File: LECTURE<br>File: OCCUPY<br>File: PARLIAMENT<br>File: TEN<br>File: 1936	File: SELFISH<br>File: THEMSELVES<br>File: UNIVERSITY<br>File: WINNER<br>File: 11<br>File: 50<br>File: ABLAZE<br>File: ABOVE<br>File: ACCOUNT<br>File: ACTUAL<br>File: AGE<br>File: APPLE<br>File: AVERAGE<br>File: DRAW<br>File: FACEBOOK<br>File: FLAT<br>File: FOLLOWER<br>File: GREAT<br>File: NOISE<br>File: PERCENT<br>File: PLACE<br>File: REMEMBER<br>File: SET<br>File: TWITTER<br>File: A-G-I-T-A-T-E-D<br>File: AFTER<br>File: AHEAD<br>File: ALLIANCE<br>File: ALLIANCE<br>File: ALMIGHTY<br>File: AMEN<br>File: CLASH<br>File: DAVID<br>File: DISCOVER<br>File: FLOWER	File: FAITH<br>File: LION<br>File: MATHEMATICS<br>File: RING<br>File: SALT<br>File: SMOKE<br>File: TOMOROW<br>File: ANOTHER<br>File: CARPENTER<br>File: EXAGGERATE<br>File: EXAGGETATE<br>File: EXCECUTIVE<br>File: EXCRETION<br>File: EXCUSE<br>File: EXECUTIVE<br>File: LIVER<br>File: PAPER<br>File: RESPONSIBILITY<br>File: SIZE<br>File: TIE<br>File: CHARACTER<br>File: EXCESS<br>File: EXHAUST PIPE<br>File: EXHAUST<br>File: EXHIBIT<br>File: EXPAND<br>File: EXPEL<br>File: EXPENDITURE<br>File: JUMP<br>File: KNO0WLEDGE<br>File: MOST<br>File: TRIP<br>File: ACTOR<br>File: BICYCLE<br>File: BIOLOGY

File: ADVANCE<br>File: APPROACH<br>File: ATITUDE<br>File: CHALLENGE<br>File: COLLEGE<br>File: COLORFUL<br>File: DECORATION<br>File: FEARLESS<br>File: FEATHER<br>File: FEATURE<br>File: GENTLE<br>File: GOLD<br>File: HEAD-ON<br>File: HIGHLIGHT<br>File: INSPIRE<br>File: INTRODUCE<br>File: LEADER<br>File: MEDAL<br>File: OWNER<br>File: PILLOW<br>File: SAFETY<br>File: SOFT<br>File: SOLVE<br>File: SPEAK<br>File: SPORT<br>File: TOUCH<br>File: USER<br>File: BACK<br>File: NEVER<br>File: SEAT<br>File: SEMEN<br>File: SENATE<br>File: SENATOR<br>File: BANKRUPT<br>File: BRIDE<br>File: BRIDEGROOM<br>File: EMPHASIZE<br>File: ESTEEM<br>File: HONEST<br>File: PANEL<br>File: RELIANCE<br>File: RELIANT<br>File: SELDOM<br>File: SELECT<br>File: SELF-ESTEEEM<br>File: SELF-ESTEEM<br>File: SELF-RELIANT<br>File: SUGGESTION	File: FORM<br>File: GROUP<br>File: PRAYER<br>File: RIVER<br>File: TEACHER<br>File: ALTERNATE<br>File: DOG<br>File: EQUAL<br>File: RAIN<br>File: REST<br>File: SING<br>File: TIRED<br>File: ANYTICLOCKWISE<br>File: ANYWAY<br>File: APPEAR<br>File: APPEARS<br>File: APPPEAR<br>File: APPROPXIMATE<br>File: APPROVE<br>File: APPROXIMATE<br>File: BADO<br>File: BILLION<br>File: COST<br>File: DISTANCE<br>File: EVENING<br>File: EVERY<br>File: FOCUS<br>File: LAWYER<br>File: RIVER<br>File: ROTATION<br>File: THINGS<br>File: THURSDAY<br>File: TOTAL<br>File: TV<br>File: A-P-R-O-N<br>File: A-R-C-H-I-V-E<br>File: A-R-C<br>File: APRON<br>File: ARAB<br>File: ARAB<br>File: CIRCLE<br>File: HISTORY<br>File: ARMPIT<br>File: ARMY<br>File: AROUSE<br>File: ARRANGEMENT<br>File: ARRANGMENT<br>File: ARREST	File: CARE<br>File: DRAMMER<br>File: EXPERIENCE<br>File: EXPERIMENT<br>File: EXPLAIN<br>File: EXPORT<br>File: EXPRESS<br>File: EXPRESSION<br>File: FACIAL<br>File: STREET CHILDREN<br>File: WITH<br>File: AEROPLANE<br>File: BEANS<br>File: EXTRA<br>File: EXTRACT<br>File: EXTREME<br>File: GRACE<br>File: JUICE<br>File: MIXTURE<br>File: MNE<br>File: SHAVE<br>File: TOOTH<br>File: WEATHER<br>File: EYE LASH<br>File: EYEBALL<br>File: EYEDROP<br>File: EYELASH<br>File: EYELASHES<br>File: GOAT<br>File: MASK<br>File: AGAINST<br>File: EMPLOYER<br>File: FILE<br>File: FLOUR<br>File: GRIEF<br>File: GRIEVANCE<br>File: GRILL<br>File: LUNCH<br>File: OVERCOME<br>File: PEPPER<br>File: WISH<br>File: AUNT<br>File: C-A-L-C-I-U-M<br>File: EVERYDAY<br>File: FRUSTRATION<br>File: GRANDMOTHER<br>File: GROAN<br>File: GROCERY

File: SUICIDE	File: BANK	File: GROUNDNUT
File: WASH	File: BOTH	File: GROUNDNUTS
File: DISSAPOINT	File: CURIOUS	File: GUAVA
File: GLASS	File: CUSTOMER	File: HERBAL
File: GREEN	File: FLOWER	File: LEAVE
File: K-A-U-N-D-A	File: FUNERAL	File: M-A-R-K-E-T
File: SACK	File: GREAT	File: MINUTE
File: SACKS	File: OMORROW	File: ONLY
File: SUIT	File: SMELL	File: OPEN
File: SUKUMA WIKI	File: A-R-T-I-F-I-C-I-A-L	File: PROTEIN PROTEIN
File: VEGETABLE	File: A-T-I-F-I-C-I-A-L	File: SAME
File: AUGUST	File: ARROWROOT	File: ALLOWANCE
File: MOMBASA	File: ART	File: CORRECT
File: SUN	File: ARTCLE	File: GUESS
File: SUNSHINE	File: ARTICLE	File: GUILT
File: SUPER MARKERT	File: ARTS	File: SHAME
File: SUPERMARKET	File: INTERESTING	File: SPEAKER
File: BURN	File: LIMB	File: WRONG
File: CASH	File: NEWSPAPER	File: B-E-E-R
File: FAMILY	File: PICTURE	File: CHEEWING GUM
File: INDIAN	File: SHOOT	File: CHEW
File: IT	File: SPEAR	File: GUITAR
File: NATIONAL	File: VOICE	File: GULP
File: OBSERVE	File: ARTIST	File: GUM
File: OXYGEN	File: ASCENSION	File: GUM
File: PROTECTIVE	File: ASHDAY	File: GUN
File: SCARCE	File: ASPRIN	File: GYM
File: SUCCESSFUL	File: ASSESS	File: H-O-R-R-O-R
File: SUDINE	File: CATHOLIC	File: HOSPITAL
File: SUGERY	File: DAY	File: LOUD
File: SUPERVISE	File: DRAWING	File: RENT
File: SUPERVISER	File: EARLY	File: SWALLOWED
File: SUPPER	File: EXCELLENT	File: BAND
File: SUPPLY	File: FERTILIZER	File: BEHAVOUR
File: SURE	File: INCREAMENT	File: BUILT
File: SURGEON	File: J-O-E	File: CLIP
File: SURGERY	File: PRICE	File: HABIT
File: SURPLUS	File: SINGLE	File: HABITATION
File: THIEF	File: SPECIAL	File: HAIR BAND
File: BRAKE	File: THOUSAND	File: HAIR CLIP
File: C-B-C	File: ACIDENT	File: NEAR
File: DUCK	File: ATHLETIC	File: SIGN
File: RAT	File: HOLD	File: ABUSE
File: SWIM	File: OBJECT	File: ACTIVITY
File: SWITCH	File: OBSTACLE	File: HAIR DRESSER
File: SYLLABUS	File: CIRCUMCISION	File: HALL
File: SYNAGOGUE	File: CONTRIBUTION	File: HALLUCINATE
File: TABLET	File: FAITHFUL	File: HAMBURGER

File: TAIL<br>File: TEETH<br>File: AFRICAN<br>File: COUGH<br>File: CUNNING<br>File: JOKE<br>File: STOMACH<br>File: SURPRISE<br>File: SWELL<br>File: SYRINGE<br>File: SYRUP<br>File: TALENT<br>File: UNCLE<br>File: CITIZEN<br>File: COMPOUND<br>File: CONTINENT<br>File: HEALTHY<br>File: HEAVY<br>File: J-O-N-A-H<br>File: KNEAT<br>File: NAME<br>File: NANNY<br>File: PILL<br>File: POTATO<br>File: S-H-A-R-K<br>File: S-W-E-D-E-N<br>File: S-W-E-D-EN<br>File: SWEAT<br>File: SWEATER<br>File: SWEEP<br>File: YELLOW<br>File: DEEP<br>File: DIAGNOSE<br>File: IMMUNE<br>File: PANIC<br>File: S-I-P-H-I-L-I-S<br>File: S-Y-P-H-I-L-I-S<br>File: SHALLOW<br>File: SPOON<br>File: SUPERVISION<br>File: SUPREMACY<br>File: SUPRESS<br>File: SWAMP<br>File: SYPHILIS<br>File: TAILORING<br>File: COLLAGE<br>File: COLLAPSE<br>File: COLLAR	File: H-O-S-T<br>File: K-O-R-I-R<br>File: NUTRIENT<br>File: OFFER<br>File: OFFERING<br>File: OGRE<br>File: OMENA<br>File: ORPHAN<br>File: P-A-R-A<br>File: REFUSE<br>File: UGALI<br>File: AIM<br>File: FARMWORK<br>File: FASHION<br>File: FEDERATION<br>File: FEEDBACK<br>File: FESTIVAL<br>File: FOREPLAN<br>File: GOAL<br>File: IMPROVE<br>File: JOIN<br>File: ORGANIZE<br>File: PART<br>File: POLICY<br>File: POSITIVE<br>File: PROMOTE<br>File: REALTION<br>File: RESOURCE<br>File: REWARD<br>File: TRIBE<br>File: UNIQUE<br>File: UNITY<br>File: WORKER<br>File: CLEANSE<br>File: COAT<br>File: CONNECTION<br>File: CRUCIAL<br>File: DOOR<br>File: FASTEN<br>File: FLIGHT<br>File: HIGHWAY<br>File: INTERNET<br>File: MAINTAIN<br>File: MARATHON.<br>File: OUTSIDE<br>File: RACE<br>File: ROOT<br>File: RUN	File: YOUTH<br>File: HAND BAG<br>File: HANDBAG<br>File: HANDSOME<br>File: HANDWRITING<br>File: HIMSELF<br>File: LATER<br>File: EMBRACE<br>File: HAPPINESS<br>File: HARBOR<br>File: HARBOUR<br>File: HARM<br>File: HARMONY<br>File: HARRASS<br>File: PANGA<br>File: WOKER<br>File: FIGHT<br>File: HARVEST<br>File: HAT<br>File: HUT<br>File: HEADING<br>File: HEADQUARTER<br>File: HEADSCARF<br>File: MBALE<br>File: QUARTER<br>File: SCARF<br>File: THANK<br>File: VIHIGA<br>File: BASIC<br>File: BELIEVE<br>File: EAR<br>File: HEALTHCARE<br>File: HEARING AID<br>File: HEART BEAT<br>File: CATTLE<br>File: ELEPHANT<br>File: GENE<br>File: HEN<br>File: HERD<br>File: HERDS<br>File: HEREDITARY<br>File: MAASAI<br>File: PEN<br>File: PROPERTY<br>File: ROTTEN<br>File: ALONE<br>File: HERO<br>File: HEROINE

File: COLLECTION	File: SAFETY BELT	File: HERSELF
File: DIRT	File: SECURE	File: NEWS
File: LORRY	File: SPEED	File: PARTICIPATE
File: P-E-N-D-O	File: SUNRISE	File: SECRET
File: SERVE	File: WILLPOWER	File: AGGRESSIVE
File: STUDY	File: ADVISE	File: CLIMB
File: TAX	File: ASK	File: FARM
File: BLIND	File: AVOID	File: HILL
File: BRITISH	File: BLESSING	File: HINDU
File: CLOUR	File: CONSEQUENCE	File: HIPPO
File: COLONISE	File: DRIVER	File: HIPPOPOTAMUS
File: COLONIZE	File: FATAL	File: KISII
File: COLUMN	File: FATHER-IN-LAW	File: SECONDARY
File: MEN	File: FOOTBALL	File: VERY
File: NATURE	File: FOR	File: ANNOINT
File: TANZANIA	File: FORGIVENESS	File: CONTROL
File: BLLIND	File: IF	File: DISCIPLES
File: BLURRED	File: INTAKE	File: FAVOURITE
File: CABBAGE	File: JUNK	File: GAME
File: CLEAR	File: KIND	File: HOBBY
File: CO-MANAGE	File: LEAD	File: HOCKEY
File: COLOUR	File: LOW	File: HOLDER
File: COLUOR	File: MEDIC	File: HOLYSPIRIT
File: COMB	File: MILK	File: HOMABAY
File: MANAGEMENT	File: MISTAKE	File: PLAYER
File: MANAGEMENT	File: REDUCE	File: READING
File: PAINT	File: RESPECTFUL	File: SEX
File: PENCIL	File: SERMON	File: TRANSMITT
File: CLASS	File: SERVICE	File: GUARD
File: COCOA	File: ALTAR	File: INSPECT
File: COMA	File: BIRTH	File: APOLOGY
File: COMBINATION	File: CARRY	File: CONFERANCE
File: COMFORT	File: CHRIST	File: DELEGATE
File: COMMA	File: CHTISTMAS	File: DELETE
File: DANGER	File: DEVELOP	File: DELICOUS
File: EFFECTIVE	File: EUCHARIST	File: DELIVER
File: PUT	File: EUROPE	File: DEMAND
File: COMMANDMENT	File: EVALUATE	File: EXPLANATION
File: COMMEMMORATE	File: EVANGELIZE	File: MESSAGE
File: COMMENT	File: FIRST	File: PROCESS
File: COMMISSION	File: GATHER	File: VIDEO
File: DEATH	File: PERFORMANCE	File: CHIEF
File: PRIEST	File: SUNDAY	File: CLOCKWISE
File: COMPANION	File: VALUE	File: FROM
File: COMPASS	File: ACTION	File: INSECT
File: POINT	File: BOSS	File: INVOLVE
File: COMPLETE	File: CELEBRATION	File: LAST
File: COMPLICATE	File: CHEETAH	File: LIQUID

File: COMPOSITION	File: COLOR	File: MALARIA
File: DISCUSSION	File: DEMONSTRATOR	File: MOSQUITO
File: ISSUE	File: EVENT	File: STING
File: POOR	File: EVERLASTING	File: STINGY
File: COMPRISE	File: EVICT	File: STIR
File: CONCEAL	File: EVIDENCE	File: STITCH
File: CONCEIVE	File: EVIL	File: STITCHED
File: CONCENTRATE	File: GREY	File: STITCHES
File: CONCEPT	File: HEAVEN	File: ATTITUDE
File: CONCERT	File: MONKEY	File: HURT
File: CONCIEVE	File: RUDE	File: K-A-M-B-A
File: DATA	File: THOUGHT	File: KEEPER
File: EFFORT	File: AGRICULTURE	File: KIKUYU
File: FARMER	File: EHAVIOUR	File: POSSIBLE
File: R-U-T-H	File: EXAMINATION	File: RETIRE
File: C-O-N-E-L-E-T	File: EXCHANGE	File: sSTORE
File: C-O-N-E-LE-T	File: CONTACT	File: STOCKING
File: CERTIFICATE	File: CONTAIN	File: STOMACH ACHE
File: CONCLUSION	File: CONTRACT	File: STOOL
File: CONDOM	File: AFTER	File: TRADITIONAL
File: CONDUCT	File: AT	File: U-L-C-E-R-S
File: CONDUCTOR	File: CLASSROOM	File: AWAY
File: CONLETE	File: EMPLOYEE	File: CALM
File: CONVETE	File: FILL	File: FALL
File: MOUNTAIN	File: HIRE	File: HOT
File: ON	File: JOY	File: LINE
File: PREGNANCY	File: MOMENT	File: PRODUCE
File: PREVENT	File: MONITOR	File: RULER
File: YOURSELF	File: MONTH MONTH	File: SAFE
File: CONFIDENT	File: SALARY	File: STEAM
File: CONFIDENTIAL	File: THE VOICE AND SIGNNING DIFFERS	File: STOREY
File: CONFIRM		File: STORM
File: DETAIL	File: DISAPEAR	File: STOVE
File: POSSILBLE	File: DOMITORY	File: STRANGE
File: ANY	File: EACH EACH	File: TRADITIONAL
File: COMFLICT	File: LAUGH	File: B-E-D
File: CONCIOUS	File: M-O-O-D	File: BEAT
File: CONFLICT	File: MOOD	File: DEADLINE
File: CONFUSE	File: MOON	File: H-A-S-T-E
File: CONGO	File: MOP	File: HEAD MASTER
File: CONJUCTION	File: SKY	File: KENYAN
File: CONJUNCTION	File: BATH	File: S-T-R-I-N-G
File: EXPECT	File: C-H-A-N-N-E-L	File: S-T-R-I-P
File: FACT	File: DURING	File: S-T-R-I-V-E
File: RESOLUTION	File: EDUCATE	File: SPLIT
File: RESOLVE	File: HOW MANY	File: STRESS
File: ADVICE	File: KING	File: STRIDE
File: CAUTIOUS	File: LAPTOP	File: STRIDES

File: CONSIDER<br>File: CONSTIPATION<br>File: CONSTITUENCY<br>File: CONSTRUCT<br>File: EMUHAYA<br>File: OFFICE<br>File: SENTENCE<br>File: WON<br>File: BUST<br>File: CONSULT<br>File: CONSUMER<br>File: INCONVINIENT<br>File: MARRY<br>File: BROKE<br>File: COOKER<br>File: COOL<br>File: GAS<br>File: HUSBAND<br>File: MICHINE<br>File: STICK<br>File: STICT<br>File: COOPERATION<br>File: COORDINATION<br>File: COPY<br>File: CORDINATION<br>File: FIVE<br>File: LIBRARIAN<br>File: THE<br>File: THEM<br>File: BURRTY<br>File: BURRY<br>File: COORDINATE<br>File: COPY<br>File: CORPSE<br>File: CORRESPONDENT<br>File: DOWN<br>File: HARRY<br>File: HURRY<br>File: PROFESSOR<br>File: TEAM<br>File: Themselves<br>File: THIRTY<br>File: THY<br>File: CITY<br>File: COACH<br>File: CONSTUME<br>File: CORRUPTION<br>File: COSMETIC	File: NORTH<br>File: SEASON<br>File: SOLOMON<br>File: TECHNOLOGY<br>File: WIFI<br>File: WINNOW<br>File: WINTER<br>File: WIRE<br>File: WIRELESS<br>File: WISDOM<br>File: CONTENT<br>File: INCOME<br>File: M-O-C-K<br>File: M-O-L-O<br>File: MOBILE<br>File: MOCK<br>File: MODE<br>File: MODERN<br>File: MODEST<br>File: MOI<br>File: REPUBLIC<br>File: SECOND<br>File: TRANSPORT<br>File: TRIBALIST<br>File: TUESDAY<br>File: AMBULENCE<br>File: INDUSTRY<br>File: J-AM-E-S<br>File: J-O-H-N<br>File: JACKET<br>File: L-E-A-T-H-E-R<br>File: M-O-N-O<br>File: MIX<br>File: MUCH<br>File: ORGANISATION<br>File: S-T<br>File: T-SHIRT<br>File: TEXTILE<br>File: THESE<br>File: W-O-O-L-L-E-N<br>File: WICHCRAFT<br>File: WITCH<br>File: WITCHCRAFT<br>File: WITHDRAW<br>File: WITHIN<br>File: C-O-N-V-I-N-I-E-N-T<br>File: COMPUTER<br>File: CONFERENCE	File: STROKE<br>File: THEIF<br>File: TOOK<br>File: WORKHARD<br>File: ANNOY<br>File: NECESSARY<br>File: NOTING<br>File: POWDER<br>File: STRUCTURE<br>File: STUBORN<br>File: STUFF<br>File: SUB MERGE<br>File: SUBLIME<br>File: T-I-T-A-N-I-C<br>File: TRANSFER<br>File: ARROGANT<br>File: BREATH<br>File: DARK<br>File: HELPER<br>File: IRON<br>File: MUHORONI<br>File: SUBTRACT<br>File: SUCK<br>File: SUDAN<br>File: SUDDEN<br>File: SUFFOCATE<br>File: SUFURIA<br>File: SUGAR CANE<br>File: SUGAR CANE<br>File: SUGGEST<br>File: CONFERENCE<br>File: CONIVINIENT<br>File: CONVERSATION<br>File: CONVICT<br>File: CONVINE<br>File: CONVINIENT<br>File: WONDERFUL<br>File: WONDERFULL<br>File: WORSHIPPER<br>File: ABSOLUTE<br>File: AS<br>File: BAIT<br>File: CROWD<br>File: DWARF<br>File: ELECTRIC<br>File: ENGINEERING<br>File: GARDEN<br>File: T-R-I-N-I-T-Y

File: COSTUME<br>File: COTTON<br>File: FAIL<br>File: HER<br>File: TALK<br>File: TORN<br>File: COUNCILOR<br>File: COUNSEL<br>File: COUNT<br>File: DIRECTOR<br>File: EMERGE<br>File: IGNORE<br>File: MEDIA<br>File: MUSICIAN<br>File: CROTCHET<br>File: CRUCIFY	File: COUPLE<br>File: COURAGE<br>File: COUSIN<br>File: DIVORCE<br>File: FRIDAY<br>File: PASSION<br>File: STUIDY<br>File: COVER<br>File: COWARD<br>File: CREAT<br>File: CREATIO<br>File: CREATION<br>File: CROWL<br>File: SNAKE<br>File: CRIPPLE<br>File: CROCODILE	File: THROUGH<br>File: VERB<br>File: WORKPLACE<br>File: WORM<br>File: WORRY<br>File: WORSHIP<br>File: WORSIPPER<br>File: WORST<br>File: BORE<br>File: THEORY<br>File: BUSH<br>File: C-R-I-P-P-L-E<br>File: C-R-O-O-K<br>File: C-R-O-T-C-H-E-T<br>File: CRICIFY
